# Traditional Chinese Medicine (TCM) and Herbal Hepatotoxicity: RUCAM and the Role of Novel Diagnostic Biomarkers Such as MicroRNAs

**DOI:** 10.3390/medicines3030018

**Published:** 2016-07-19

**Authors:** Rolf Teschke, Dominique Larrey, Dieter Melchart, Gaby Danan

**Affiliations:** 1 Department of Internal Medicine II, Division of Gastroenterology and Hepatology, Klinikum Hanau, D-63450 Hanau, Teaching Hospital of the Medical Faculty of the Goethe University, Frankfurt/Main D-63450, Germany; 2 Department of Liver and Transplantation—IRB-INSERM (Institut de Recherche Biologique-INstitut de la Santé Et de la Recherche Médicale) 1183, Saint Eloi Hospital, Montpellier University, 34295 Montpellier, France; dom-larrey@chu-montpellier.fr; 3 Competence Centre for Complementary Medicine and Naturopathy (CoCoNat), Klinikum rechts der Isar, Technische Universität München, Munich D-80801, Germany; ga39fos@mytum.de; 4 Institute for Complementary and Integrative Medicine, University Hospital Zurich and University of Zurich, Zurich CH-8091, Switzerland; 5 Pharmacovigilance Consultancy, Paris 75020, France; gaby.danan@gmail.com

**Keywords:** Traditional Chinese Medicine, herbal TCM hepatotoxicity, diagnostic biomarkers, RUCAM, pyrrolizidine alkaloids, HLA, microsomal epoxide hydrolase, pyrrole-protein adducts, metabolomics, microRNA

## Abstract

**Background**: Traditional Chinese Medicine (TCM) with its focus on herbal use is popular and appreciated worldwide with increased tendency, although its therapeutic efficacy is poorly established for most herbal TCM products. Treatment was perceived as fairly safe but discussions emerged more recently as to whether herb induced liver injury (HILI) from herbal TCM is a major issue; **Methods**: To analyze clinical and case characteristics of HILI caused by herbal TCM, we undertook a selective literature search in the PubMed database with the search items Traditional Chinese Medicine, TCM, alone and combined with the terms herbal hepatotoxicity or herb induced liver injury; **Results**: HILI caused by herbal TCM is rare and similarly to drugs can be caused by an unpredictable idiosyncratic or a predictable intrinsic reaction. Clinical features of liver injury from herbal TCM products are variable, and specific diagnostic biomarkers such as microsomal epoxide hydrolase, pyrrole-protein adducts, metabolomics, and microRNAs are available for only a few TCM herbs. The diagnosis is ascertained if alternative causes are validly excluded and causality levels of probable or highly probable are achieved applying the liver specific RUCAM (Roussel Uclaf Causality Assessment Method) as the most commonly used diagnostic tool worldwide. Case evaluation may be confounded by inappropriate or lacking causality assessment, poor herbal product quality, insufficiently documented cases, and failing to exclude alternative causes such as infections by hepatotropic viruses including hepatitis E virus infections; **Conclusion**: Suspected cases of liver injury from herbal TCM represent major challenges that deserve special clinical and regulatory attention to improve the quality of case evaluations and ascertain patients’ safety and benefit.

## 1. Introduction

The reputation of herbal Traditional Chinese Medicine (TCM) has substantially improved within the past few years [1,2,3]. This was achieved through efforts closing up its gaps with modern medicine [4,5], considering clinical aspects [6,7,8,9,10,11,12,13,14,15], as well as researching and developing new drugs [16,17,18]. The Nobel Prize for Medicine or Physiology 2015 will certainly further inspire modern TCM research in the near future, although the Nobel Prize committee did not specify that the prize was for TCM [19]. Instead, the 2015 Nobel Prize was awarded to Youyou Tu for her pioneering work in drug development originating from herbal TCM and her discovery of artemisinin for the treatment of malaria [19,20]. Artemisinin is derived from the herbal TCM Qing Hao (*Artemisia annua* L.), known for more than two thousand years as a Chinese herbal medicine for various ailments [19,20,21,22,23]. The initial breakthrough in the discovery of artemisinin dates back to 4 October 1991, when an extract derived from *Artemisia annua* was found to be 100% effective against parasitemia in mice infected with *Plasmodium berghe*i and in monkeys with *Plasmodium cynomolgi* [22]. After ascertaining that the extract was safe for human use, clinical efficacy was tested in the Chinese Hainan province in trials with patients infected with both *Plasmodium vivax* and *Plasmodium falciparum* [22]. The results were encouraging: patients treated with the extract experienced rapid disappearance of symptoms such as fever and decrease of parasitemia, whereas patients receiving chloroquine did not. Artemisinin and its derivatives such as artesunate and arthemeter are now successfully used to treat patients with malaria by *Plasmodium vivax* or *Plasmodium falciparum*, preferring a combination with antimalarial drugs other than artemisinin, called artemisinin-based combination therapies (ACTs), and banning artemisinin-based monotherapy that may promote the risk of antimalarial drug resistance [23]. Malaria is transmitted among humans by female mosquitoes of the Anopheles genus, which take blood meals from patients with malaria to carry out egg production; such blood meals are the link between the human and the mosquito hosts in the parasite life circle and enable the transfer of malaria through infected mosquitos by biting humans [24]. Based on treatment with ACTs together with other approaches such as use of insecticides and insecticide-treated mosquito nets, the WHO stated that the number of patients with malaria and malaria-related mortality has been drastically reduced within recent years [23], and there is no question that most of this improvement is attributable the work of Youyou Tu with *Artemisia annua*.

From discovering the TCM herb *Artemisia annua* and the active molecule to developing the beneficial drug was a long but successful process in drug research and development [22]. This is not in line with mainstream philosophy of herbal TCM that prefers the use of a bundle of herbs rather than to focus on a single herb or even a single molecule [25,26], as opposed to what Youyou Tu did [22]. In 1996, she started her project against malaria by investigating more than 2000 Chinese herbal preparations and identified 640 hits that had possible antimalarial properties. More than 380 extracts obtained from around 200 herbs were evaluated, and a high antimalarial activity was found in an extract derived from *Artemisia annua* as prepared according to “A Handbook of Prescriptions for Emergencies” by Ge Hong (from 248 to 346 AD). Originating from artemisinin with a molecular weight of 282 Da and a molecular formula of C_15_H_22_O_5_, dihydroartemisinin was found to be more stable and 10 times more effective than artemisinin, and adding a hydroxyl group to the molecule provided more opportunities for developing new artemisinin derivatives through esterification [22]. The fascinating route from a TCM herb via a molecule to a drug is finally highlighted again at the molecular level, now in the human erythrocytes infected with plasmodia [20]. The molecular basis of treatment efficacy includes among several mechanisms the cleavage of artemisinin’s endoperoxide bridge by a Fe(II) Fenton, which is facilitated by heme–iron originating from hemoglobin degradation in the parasites’ food vacuoles [20,27]. Autodigestion of plasmodium food vacuoles is achieved following their damage by hydroxyl radicals and superoxide anions [20]. Consequently, artemisinin with its derivatives is a drug with a specific molecular target in a specific parasitic disease [20,27]; toxicity has not been found in clinical studies with normal doses [27]. Expectations are high that more hidden drug champions can be derived from herbal TCM with high efficacy and little harm, providing a favorable benefit-risk profile.

As opposed to artemisinin and its derivatives, which lack proven hepatotoxic adverse reactions, there is major clinical concern that the use of various TCM herbs or herbal products may be associated with the risk of toxic liver disease [7,28,29]. Herb induced liver injury (HILI) from herbal TCM may be severe and rarely leads to acute liver failure (ALF), which is often life-threatening unless liver transplantation is performed. These serious clinical courses are a particular challenge for clinicians caring for these patients with suspected HILI, considering that the indication of therapy is often vague.

In this review article, we focus on potential hepatotoxic TCM herbs and will discuss typical clinical features of HILI cases, considering also specific TCM herbs with specific types of liver injury. We will analyze the issue of specific diagnostic biomarkers and valid causality assessment, providing evidence that is best achieved by the use of the liver specific RUCAM (Roussel Uclaf Causality Assessment Method) or positive results from unintentional reexposure. Finally, confounding variables will be discussed as well as clinical and regulatory strategies to minimize the risk of hepatotoxicity in patients treated with TCM herbs.

## 2. Data Sources and Searches

### 2.1. Search Terms

We searched the PubMed database to identify publications such as case reports, case series, and review articles for the following terms: Herbal TCM or herbal Traditional Chinese Medicine; both terms were combined with hepatotoxicity or liver injury. This allowed the identification of publications of hepatotoxicity combined with herbal TCM or herbal Traditional Chinese Medicine, which provided hits of around 27,100 or 98,000; for liver injury combined with herbal TCM or Traditional Chinese Medicine, 1,320,000 or 1,970,000 hits were presented. These figures indicate a high internet presence for the chosen search terms. The first 100 hits of publications in each category were usually considered. In addition, we used our large and actualized personal scientific archives, which contain original full-length publications relating to the search terms, covering the years from 1983 to early 2016.

### 2.2. Data Extraction

Prior to our analysis, the publications were assessed regarding their scientific and clinical quality, appropriateness and relevance for the topic of this article. Publications of good quality were preferred and considered for evaluation. The focus of our search was on publications in the English language, but few reports in other languages were also considered if they were of significant clinical importance and added to present knowledge. The literature search was limited since many reports of HILI from herbal TCM were in the Chinese language without an English abstract. Consequently, these Chinese publications were outside the focus of our present review article. Publications were also manually searched for additional publications not yet identified. The literature search ended on 15 April 2016. Given the large number of publications of liver injury by TCM herbs, not all reports could be selected. Attempts were made to search for the most relevant publications for analysis and inclusion in the reference list of this review.

## 3. Herbal Traditional Chinese Medicine, Its History, Philosophy and Challenges

### 3.1. History

TCM herbs and Indian herbal Ayurvedic medicine are among the few ancient medicines that have survived many other medicine cultures around the world. Since two thousand years, numerous herbal formulas were widely used in China for the treatment of various minor ailments and diseases. The TCM philosophy and its associated herbal TCM created curiosity and skepticism in Western countries, since transparency is only partially provided [6,10]. Ancient medical books are historical examples for this broad empirical knowledge. Among these are formulas for 52 diseases (Wu Shi er Bing Fang, second century BC), Yellow Emperor’s Canon of Internal Medicine (Huang Di Nei Jing, Later Han from 25 to 220 AD), and Prescription from the Golden Chamber (Shang Han Lun and Jin Gui Yao Lue, from 200 BC to 200 AD) [30,31,32]. For some years, Chinese intellectuals, scientists, physicians, and politicians have shown a clear preference towards the rational Western science and medicine [33].

However, only in 2007, the Chinese Government represented by the Chinese Ministry of Science and Technology invited politicians and experts from 50 countries to draft the Beijing Declaration on Traditional Chinese Medicine (TCM) and classified TCM as part of biomedicine [33]. The future of TCM was seen in the unification of historical Chinese and modern Western medicine, based on molecular biological legitimation [33]. Part of the Chinese government would obviously favor a rigorous modernization of herbal TCM that can be used to effectively treat human diseases.

### 3.2. Philosophical Background

To understand the principles of herbal TCM therapy, some information of the philosophical background may be helpful for those not familiar with these interesting but challenging TCM concepts [6,10]. In short, core elements of the TCM philosophy are ideas primarily related to “yin and yang”, two opposing yet complementary forces; disease results from an imbalance of these forces [6]. Philosophical support is provided by the “Five Phases” as the interaction among the five elements: wood, earth, water, fire and metal [6]. To meet the requirements of herbal therapy according to TCM philosophy, herbal TCM products are commonly blends of four to six different herbs [28]. This approach allows for disease treatment via different molecular disease targets.

As opposed to modern Western medicine with clear diagnostic criteria as a prerequisite for treatment indication, such conditions are mostly vague or even missing in the context of herbal TCM diagnosis and treatment [9,10]. As an example, treatment protocol of herbal TCM is established based on a syndrome differentiation. Clinical signs and symptoms are clustered around uniquely ‘Chinese’ nosological categories and diagnostic terms, such as stagnation of meridians, liver-Qi stagnation, blood stasis, dampness, and others [34]. The doctor as specialist for TCM takes a detailed history of physical and vegetative symptoms, augmented by information gained through somatic signs. Based on this “phenomenological evaluation”, he arrives at a so-called “energetic diagnosis” that is based on the conceptual framework of TCM. For instance, rheumatoid arthritis shows so-called syndrome patterns of “wind-cold-damp”, “heat-toxicity”, or “wind-damp-hot” [35,36,37]. For conditions such as these, specific herbal TCM products are available [38,39,40].

### 3.3. Herbal TCM Use and Current Issues

Herbal TCM presents potentially great perspectives with special challenges related to its expanding worldwide use, its integration into modern Western medicine, and the approaches it offers in the research and development of new drugs [1,2,3,4,5,6,7,8,9,10,11,12]. A variety of reports and analyses focused on important issues related to the use of herbal TCM [8,9,10,41,42,43,44,45,46,47,48,49,50,51,52,53]. In particular, its integration into modern Western medicine will not easily be accomplished due to differences in philosophy and approaches of diagnoses and therapy. In addition, TCM philosophy requires that a TCM physician is involved in the treatment, who will not always be available for consumers who order TCM herbals via the internet.

Apart from these uncertainties, however, impressive data are reported for China, where the use of herbal TCM represents around 40% of all health care services [7]. In addition, the worldwide use of herbal TCM is well recognized, but robust quantitative results are not available. This lack is due to insufficient definitions of consumer cohorts or the heterogeneity of both herbal product groups and treatment modalities. For instance, in many reports on traditional medicine, traditional herbal medicine, natural medicine, and complementary and alternative medicine (CAM), there is a mix of various treatments including herbal TCM which is often not separately mentioned and discussed. A similar problem relates to reports on general TCM, which may include practices unrelated to herbal TCM such as acupuncture, moxibustion (burning a herb above the skin to apply heat to acupuncture points), Tui Na (Chinese therapeutic massage), dietary therapy, or Tai Chi (shadow boxing) and Qi Gong (practices that combine specific movements or postures, coordinated breathing, and mental focus) [6].

At least according to publications on clinical trials performed in mainland China, the focus of TCM is here clearly on herbal remedies (90.3%), followed by acupuncture (4.4%), massage (3.8%), moxibustion (1.2%), Qi Gong (0.1%), and other therapies (0.2%) [42]. Despite these accurate figures in China, a realistic impression on the quantitative use of herbal TCM in other countries requires a more sophisticated analytical approach, also to quantify the worldwide consumption of herbal TCM products. Of note, in other Asian countries, TCM became popular and is called for instance “Traditional Asian Medicine” (TAM) or “Traditional Occidental Medicine” (TOM), and in Japan also Kampo medicine [53]. The basic principles of TCM are identical or vary only a little between countries; hence, TCM as the overarching term is warranted and used in this review article in order to facilitate the discussions on the major TCM related issues.

Current issues of concern are listed with proposals as to how these problems can be solved to provide effective and safe herbal TCM medicine also in Western countries to those individuals who prefer this specific therapy instead of conventional medicines (Table 1).

### 3.4. Integration of Herbal TCM into Western Medicine

The integration of herbal TCM into modern medicine has been the subject of ongoing international discussions in the last few years [1,2,4,5,33,49,54]. Ancient herbal TCM and modern medicine have evolved under different empirical, theoretical, philosophical, and cultural conditions, each attempting to establish cornerstones of valid diagnostic and therapeutic principles and to provide efficient healthcare. However, the opinion prevailed that the situation of ancient herbal TCM is partially disappointing [10,55], requiring substantial improvements with the tentative aim to develop a pragmatic modern herbal TCM that meets the needs of modern medicine and possibly combines both medicinal cultures by bridging the gap between the herbal TCM and Western medicine [10].

### 3.5. New TCM Drugs and Approaches of Research and Development

Health care systems around the world are increasingly facing the problems of chronic illnesses and their associated cost, which may be reduced by the use of herbal TCM products. Cancer treatment is one of the most promising areas where there would be a considerable potential for the development of new drugs derived from TCM plants [8,20,49,56,57,58]. Drugs such as topotecan, irinotecan and camptothecin derivatives used for cancer therapy are good examples of drug development based on herbal TCM [57]. In the future, several herbal TCM products [57,58] including artemisinin [57] will have a high potential to advance drug discovery and development in a major world market, which stands at about $83 billion, while Europe accounts for over 50% of the total [49]. Perhaps with few exceptions [8,58], the lack of robust evidence from randomized controlled trials (RCTs) is hindering acceptance of herbal TCM by the mainstream Western healthcare [9,10,42].

### 3.6. Therapeutic Efficacy

Therapeutic efficacy is a crucial criterion for the use of herbal TCM in the face of rare serious adverse reactions such as severe liver injury or ALF [6,7]. Through randomized controlled trials (RCTs), multiple studies attempted to validly establish efficacy of treatments by herbal TCM [9,10,42,43]. In fact, publications of TCM trials are abundant [42,43], considering publication figures of around 10,000 [43] or 26,263 [42], but their scientific quality is limited [9,10,42,43]. One of these studies identified 37,252 Chinese language articles in TCM journals published in mainland China, with clinical trials recognized in 26,263 out of 37,252 articles, corresponding to 70.5% [42]. Out of these 26,263 clinical trials, 7422 were initially identified as RCTs, equivalent to 28.3%, but among these 7422 trials, only 1329 (17.9%) were truly randomized [42].

In more detail, some important methodological components of the RCTs were missing, such as sample size calculation (reported in 1.1% of RCTs), randomization sequence (7.8%), allocation concealment (0.3%), implementation of the random allocation sequence (0%), and intention to treat analysis (0%) [42]. All reports were searched according to guidelines of the Cochrane Centre, and a comprehensive quality assessment of each RCT was completed using a modified version of the CONSORT checklist [42]. The poor quality of many TCM RCTs [42] was continuously discussed in various reports during the last decades, as summarized recently [10].

Regrettably, most Cochrane systematic reviews of TCM are inconclusive, due specifically to the poor methodology and heterogeneity of the studies [9]. It is well recognized that planning and performing RCTs, data analysis and compilation are cumbersome, time consuming, and expensive [9], with additional efforts having to be put into editorial and reviewing work. Unless strict criteria are applied for clinical trials of alternative medicinal systems including herbal TCM, these studies will not be regarded as valid. For most analyses such as those evaluated in this review, major quality criteria are violated, including primary research hypothesis formulation, clinical inclusion criteria and outcome parameters, and appropriate statistical analysis [10]. RCTs require clearly defined drug and usage characteristics, which cannot be provided for herbal TCM formulae that are prescribed individually and not uniformly for each patient.

**Figure 1:**
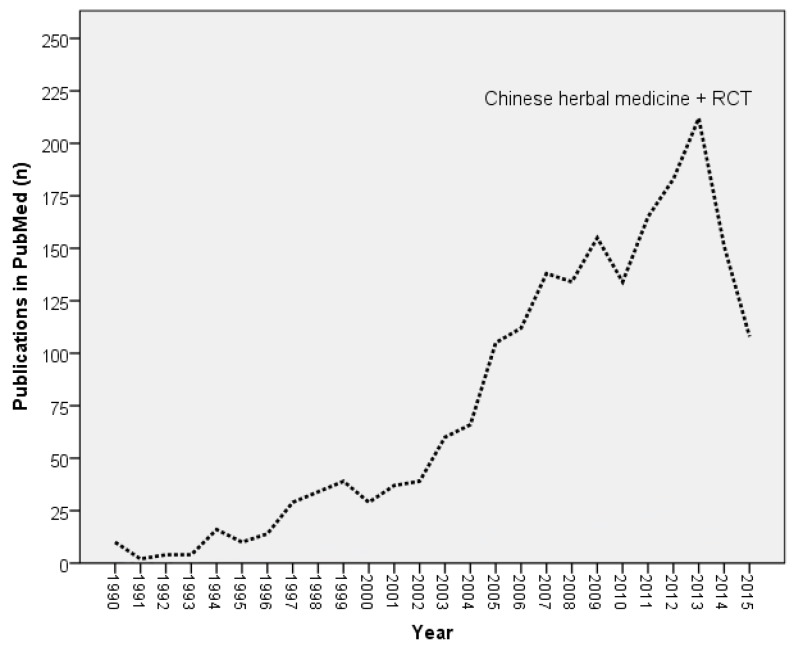
Publications in PubMed reporting RCT (randomized controlled trial) studies of herbal TCM. Reports from 1990 to 2015 (search: “TCM herbs AND randomized controlled trial”, accessed on 26 April 2016).

RCT reports of TCM herbs have been increasingly published since 1990, but the reason for a sharp drop of reports within the last two years is unclear (Figure 1).

### 3.7. Safety

In analogy to other herbs [59], TCM herbs may be classified as good ones, bad ones, and ugly ones [25]. For some herbal medicines including herbal TCM products, safety concerns have been expressed regarding rare adverse reactions that were not always transient but occasionally resulted in a severe clinical course and fatality [2,7,12,28,29]; attempts to ignore severe adverse reactions should be resisted [55]. Under these conditions, manufacturers and regulatory agencies are obliged to provide good product quality of efficient herbal products including herbal TCM, ascertaining consumer safety [12,50,60,61]. Certainly, the majority of TCM plants are harmless and well tolerated by most consumers [3,13]. As with any conventional treatment, the use of herbal TCM products may be associated with only mild and transient adverse effects that are without clinical relevance (Table 2) [3,13].

Adverse reactions are presented for 994 patients treated with herbal TCM as decoction in the TCM-hospital Bad Kötzting, 2013 (Table 2) [13]. Liver adaptation refers to values of ALT (Alanine aminotransferase) <5 *N* (upper limit of normal), while liver injury is based on ALT ≥5 *N* [51]. In the three cases of liver injury, RUCAM based causality was only “possible”.

Because safety aspects are an outstanding problem (Table 1) [2,28,29,60] and due to variable and disputable product quality [13,62,63,64,65], herbal TCM products require a more strict regulatory surveillance in China and elsewhere [8,12,50]. Some efforts appear promising [12], provided the principles of current Good Agricultural Practices (cGAP) and current Good Manufacturing Practices (cGMP) are fulfilled [8,12,50,60]. In essence, herbal TCM products intended for medicinal purposes and fulfilling regulatory requirements of efficacy and safety, should all be classified as regulated herbal drugs [50]. Such strict regulatory surveillance should be expanded to dietary supplements (DS); most of these include herbal ingredients [61]. The abundance of DS is frightening, as for instance more than 50,000 DS were marketed in the United States between 1995 and 2015, a real regulatory challenge [61]. A more stringent regulatory approach would reduce the number of products and allow for more transparency [50].

**Figure 2:**
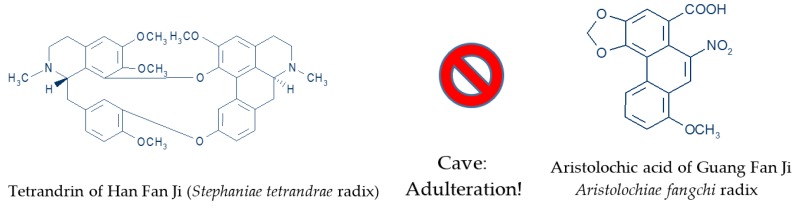
Adulteration of Han Fan Ji for Guang Fang Ji in Belgium [45,46,47].

Eighteen years ago, inconsistent botanical nomenclature of TCM herbs with similar Chinese names provoked shocking publicity of a case of falsification, when the root *of Stephania tetrandra* (Han Fang Ji) was confused with the root of *Aristolochia fangchi* (Guang Fang Ji) (Figure 2) [45,46,47]. The mix up herb intended for weight loss was consumed by women as tea medication that produced severe nephrotoxic adverse effects. The TCM *Aristolochia* herbal product contains the carcinogenic aristolochic acid [45]. Meanwhile, special quality proof methods like TLC (Thin-Layer Chromatography) and HPLC-fingerprint technique (High Performance Liquid Chromatography) of Chinese drugs was able to detect minimal concentrations of these toxic acids in single herb products or herbal drug mixtures in order to avoid cases of poisoning [48]. In the near future, the barcode fingerprint DNA analysis will also be available, in order to supplement the chromatographic analysis [48]. All these methods should help to fulfill the basic requirements for quality proof of Chinese herbs including botanical authenticity, species diversity, safety check for adulterations, processing of herbal drugs on the bench, optimal extraction methods, and standardization of herbal extract combinations [48,66].

### 3.8. Nomenclature

Nomenclature variability of individual TCM herbs and herbal TCM products such as herbal mixtures consisting of multiple TCM herbs are considered as a specific problem [52,55], especially when scientific reports have to be interpreted in relation to a TCM herb [52]. Unlike Latin scientific names, CMM names, be they Chinese, pin yin, English, or according to pharmacopoeia, have not been standardized and their use, spelling and occasionally even the plant species to which they refer vary from publication to publication [52]. The monographs in the Chinese Pharmacopoeia also vary in their species delimitation from one edition to the next, and Latin names should never be derived from Chinese Materia Medica (CMM) names alone. Available as proprietary medicines or crude herbal mixtures, herbal TCM formulae may also differ in composition depending on country or origin, manufacturer or even individual practitioner [51]. These difficulties are amplified by the large number of TCM plants in China [10].

Currently, around 13,000 herbal preparations are listed in the CMM, and are available in China [67,68], being officially recognized and described in detail by the Chinese Pharmacopoeia [67,69], including herbs commonly used, regional variations and folk medicine variants. The CMM [69] is a reference book that also describes thousands of plant preparations [6], including some nonbotanical elements (animal parts and minerals) [6,53,55,67] incorrectly classified as herbal medicines [34]. Outside of China, only around 500 Chinese herbs are commonly used [67].

## 4. TCM Herbs with Published Claims of Hepatotoxic Potential

### 4.1. Compilation of TCM Herbal Products with Reported Claims of Liver Injury

It is particularly challenging to compile all potentially hepatotoxic TCM herbs and herbals products, but an attempt is warranted to provide at least first data on this issue. Several limitations of this approach have to be acknowledged. Completeness of the listing is hardly achievable since systematic compilations of TCM HILI cases are not available. There is also the problem of how to define criteria of the cases that should be included.

In all suspected cases of HILI caused by TCM as published in scientific journals, the authors claim causality for the reported herbal TCM product; otherwise the submitted paper would have not been accepted for publication. Assumed causality was mostly based on clinical judgment, rarely on the results of liver specific causality assessment methods [70,71,72,73,74,75,76,77,78,79,80,81,82,83,84,85,86,87,88,89,90,91,92,93,94,95,96,97,98,99,100,101,102,103,104,105,106,107,108,109,110,111,112,113,114,115,116,117,118,119,120,121,122,123,124,125,126,127,128,129,130,131,132,133,134,135,136,137,138,139,140,141,142,143,144,145,146,147,148,149,150,151,152,153,154,155,156,157,158,159,160,161,162,163,164,165,166,167,168,169,170,171,172,173,174,175,176,177,178,179] such as RUCAM [51,180]. Among the published reports, a large number of assessments provide only a possible causality level, which is fair enough but in essence of little clinical or regulatory relevance. Published reports are therefore hardly assessable by others due to incomplete data presentation and confounding variables. These include for instance lacking a temporal relationship between product use and the occurrence of liver injury itself poorly defined, unclear product description and quality, insufficient exclusion of alternative causes including viral and namely HEV infections, and no data on preexisting liver diseases. Despite these limitations, stakeholders including regulatory agencies, physicians, and patients taking these herbal TCM products are informed that such hepatic reactions could occur (Table 3).

### 4.2. Worldwide Reports

Considering the worldwide publications preferentially in the English language, there were two reports on TCM HILI during the period from 1984 to 1993 [180]. This was followed by 20 publications from 1994 to 2003 and 55 reports from 2004 to 2013. With 28 publications for the period from 2004 to 2008 and with 27 reports from 2009 to 2013, the publication frequency was stable in recent years [180]. The variability of countries from which the reports originated is high [53]. Of note, the use of herbal TCM is popular in Germany, but liver injury appears not to be a problem [13,14,183], at least in one study, considering that RUCAM based causality in the three cases of liver injury was only possible (Table 2) [13].

Cases of suspected TCM HILI occurred in various countries and were published with details such as case series and case reports, mostly with highly informative narratives [70,71,72,73,74,75,76,77,78,79,80,81,82,83,84,85,86,87,88,89,90,91,92,93,94,95,96,97,98,99,100,101,102,103,104,105,106,107,108,109,110,111,112,113,114,115,116,117,118,119,120,121,122,123,124,125,126,127,128,129,130,131,132,133,134,135,136,137,138,139,140,141,142,143,144,145,146,147,148,149,150,151,152,153,154,155,156,157,158,159,160,161,162,163,164,165,166,167,168,169,170,171,172,173,174,175,176,177,178,179]. Additional data may be derived from review articles and other publications, which partially covered also large cohorts [181,182,184,185,186,187,188,189,190].

Clearly, many Chinese individuals consume herbal TCM products, and the majority of TCM HILI cases are obviously published in scientific journals in the Chinese language, thereby not easily assessable by Western scientists. Therefore, whatever is published in the English language in Western journals on HILI by TCM, this may just be the tip of an iceberg. The total number of regulatory HILI cases due to herbal TCM products is also unknown, as submitted to and archived in the regulatory agency, the China Food and Drug Administration (CFDA) in Beijing, also known as Center for Drug Reevaluation. Subsequently, any compilation of suspected HILI involving TCM will be necessarily incomplete [184]. For reasons of transparency and scientific evaluation, spontaneous reports of cases of HILI by TCM submitted to CFDA should be published with a narrative, a list of excluded alternative causes, and a RUCAM based causality assessment [51]. This approach would facilitate considerations on how safety of these products can be improved. The top ranking of reporting countries focused on Hong Kong, Korea, Japan, and the United States, whereas reports originating from European countries were unexpectedly scarce, as evidenced by evaluation of selected reports (Table 4).

## 5. Narratives of TCM HILI Cases

Most appreciated for case analyses are detailed descriptions of product ingredients, product use, and clinical characteristics, but many reports of TCM HILI lack such informative narratives and may easily be disqualified due to incomplete data. Rather than publishing many cases of poor quality and low causality levels, the preferred approach would be to publish fewer cases of suspected TCM HILI with RUCAM based causality gradings of “probable” or “highly probable” to ensure correctness of the TCM HILI diagnosis. Such qualified cases can then be used to characterize clinical features of patients with liver injury by a single TCM herb or herbal mixture.

### 5.1. An Shu Ling

A 42-year old woman of the United States took three different herbal medicines for insomnia [70]. The products were An Shu Ling (syn. Jin Bu Huan) as TCM, “Ignatia Amara” and “Relaxed Wanderer”. Following this treatment for 10 weeks, she experienced acute liver injury and jaundice. Poison specialists at the California Poison Control System in San Francisco searched the Poisindex (Micromedex, Englewood, CO, USA) and found the synonym Jin Bu Huan listed for An Shu Ling, its chemical analysis identified l-tetrahydropalmitine, the hepatotoxic ingredient, present also in Jin Bu Huan that also contains *Stephania sinica* among other herbs. The two other herbal products were analyzed and contained no known hepatotoxins. The product lot of An Shu Ling was confiscated from the importer-seller, and a public health warning was issued. No additional cases associated with the use of this particular product were reported. Although Jin Bu Huan itself was banned from importation into the United States due to the risk of HILI, this shipment of An Shu Ling reportedly cleared US customs because the shipping invoice mentioned only the Chinese botanical name [70]. See also Jin Bu Huan.

### 5.2. Ba Jiao Lian

After drinking infusions of the TCM Ba Jiao Lian (*Dysosma pleianthum*) taken at recommended doses, five patients in Taiwan experienced abnormal liver tests, nausea, vomiting, diarrhoea, abdominal pain, thrombocytopenia, leucopenia, sensory ataxia, altered consciousness and persistent peripheral tingling or numbness [71]. In a recent report from Taiwan, 17 cases associated with poisoning by Ba Jiao Lian were reported [72]. Podophyllotoxin is one of the main ingredients of the Ba Jiao Lian root and considered to be the toxic agent [71,72,73]. However, the increase of the aminotransferases was mild, with predominance of aspartate aminotransferase (AST) over alanine aminotransferase (ALT) [71,72,73]. The increase in AST [71] could also reflect either an isolated damage of liver mitochondria or muscular damage [71,73] because of the associated increase of creatine phosphokinase [71]. These uncertainties do not allow for the classification of Ba Jiao Lian as a hepatotoxic TCM herb, and therefore it will not further considered within this review.

### 5.3. Bai Fang

A 54-year old male patient in the United States developed subtotal liver necrosis and survived following LTX [74]. He used Bai Fang as a herbal TCM for an unknown period of time and had an acute HBV infection as concomitant disease. Bai Fang is a herbal mixture that includes *Angelica sinensis*, *Cyperus rotundus,* Ginseng, *Ligusticum wallichii*, *Paeonia alba*, and *Rehmannia glutinosa.* The possible hepatotoxic herb and its suspected ingredient are unknown. The causal role of Bai Fang may be questioned in this case report because of the concomitant acute HBV infection. No other cases were reported involving Bai Fang.

### 5.4. Bai Xian Pi

In four Korean patients, the use of the herbal TCM Bai Xian Pi (*Dictamnus dasycarpus*) was hepatotoxic, when applied as a single herb [75], and in three other patients from the United Kingdom, when it was coadministered with other herbs [76,77,78]. Fourteen patients from Korea developed ALF due to *Dictamnus dasycarpus* [79], and two other Korean patients experienced ALF that needed a liver transplant [80]. See also Chinese herbal mixtures.

### 5.5. Ban Tu Wan

A middle-aged Asian female patient living in the United States experienced ALF secondary to the use of the TCM Ban Tu Wan [81]. The components of this herbal mixture are *Angelica sinensis*, *Chaenomeles*, *Codonopsis pilosula*, *Notopterygium*, *Polygonum multiflorum*, *Rehmannia*, and *Schisandra*. The patient was evaluated for a liver transplantation but died from septicemia before this procedure [81]. No other cases caused by Ban Tu Wan were published. See also Ho Shou Wu, *Polygonum multiflorum*, Shen Min, and Shou Wu Pian.

### 5.6. Bo He

A 45-year and 46-year old men from Hong Kong with chronic HBV infection took the TCM Bo He (*Mentha haplocalyx*) in herbal mixtures that contained 11 other ingredients. Bo He is known to be hepatotoxic in the Chinese literature [82].

### 5.7. Bofu Tsu Sho San

A 37-year old Japanese woman used the herbal TCM Bofu Tsu Sho San, a Japanese kampo medicine also called Bofu Tsusho San and experienced liver injury [83]. Bofu Tsu Sho San contains 16 herbs, *Angelica*, *Atractylis*, *Cnidium*, *Gardenia*, *Ephedra*, *Forsythia*, *Glycyrrhhiza*, *Gypsum fibrosum*, *Ledebouriella*, *Mentha*, *Paeonia*, *Platycodon*, *Rheum*, *Schizonepeta*, *Scutellaria*, and *Zingiber*, as well as Kadinum (talcum powder) and sodium sulfuricum [84]. Several herbs are candidates for liver injury, including *Ephedra* providing the hepatotoxic ephedrine [85]. For *Ephedra* see also Ma Huang.

### 5.8. Boh Gol Zhee

Acute liver injury was described after the use of the TCM Boh Gol Zhee (syn. Bol Gol Zhee, Bu Gu Zhi, Bu Ku Zi, Sheng Bu Gu Zhi, Sheng Po Gu Zhi) in two patients in Korea [86,87] and in three patients in Hong Kong [88]. Boh Gol Zhee is not a herbal mixture but contains only seeds of *Psoralea corylifolia* with psoralens as possible hepatotoxic ingredients, especially when used in large amounts [86,87,88]. Psoralens are also hepatotoxic candidates in another patient, who experienced severe liver injury after the use of Indian Ayurvedic herbal products [89]. These included Bakuchi tablets with extracts from *Psoralea corylifolia* leaves with psoralens to treat her vitiligo.

### 5.9. Bupleurum 

See Chai Hu.

### 5.10. Camellia Sinensis

See Lu Cha.

### 5.11. Chai Hu

The risk of liver injury might be increased in Taiwan patients with chronic HBV infections treated with TCM products containing Chai Hu (*Bupleurum falcatum*) [90]. In particular, two products were involved: Xiao Chai Hu Tang and Long Dan Xie Gan Tang. In other patients without chronic HBV infection, herbal TCM products containing *Bupleurum* might be hepatotoxic, see for instance Da Chai Hu Tang [91], Kamishoyosan [92], and a report referring to a herbal TCM mixture [77].

### 5.12. Chaso

Six Japanese patients developed hepatic injury due to the use of the herbal weight loss aid Chaso of TCM [93]. This product contained *Camellia sinensis* (Green tea, syn. Lu Cha as TCM), the hepatotoxic *Cassia tora* (Senna), *Crataegus*, *Chrysanthenum morifolium* Ramat, *Lotus*, and *Lycium barbarum*. *Camellia sinensis* extract is a known weight loss aid, most likely present as extract in the Chaso formula to facilitate weight loss. It was not considered as the offending agent [93], since the hepatotoxicity of *Camellia sinensis* extract was unknown in 2003 when Chaso hepatotoxicity was described. Outcome was favorable in all patients including one patient treated with liver transplantation. Chemical analysis of the Chase product showed lack of fenfluramine and heavy metals such as copper, lead, bismuth, cadmium, stibium, stanum, mercury, and chromium, but *N*-nitroso-fenfluramine was found. *N*-nitroso-fenfluramine was considered as possible but not yet proven to be a hepatotoxin [93], similar to other cases of liver injury due to various other slimming aids in the United Kingdom [94], Hong Kong [95], and Japan [96], including the TCM herbal product of Onshido [93]. An additional 21 Chaso-induced cases of liver injury were reported to the regulatory agency in Japan and not further analyzed [93]. A cautionary statement of the authors asked for further toxicological analysis to demonstrate a possible hepatotoxicity of *N*-nitroso-fenfluramine, which was not established toxicologically or clinically before 2003 [93]. Toxicological evidence of its potential hepatotoxicity has not been brought since 2003, and clinical evidence will be difficult to report due to the removal of this ingredient from the market in 1997. The popular and widely used slimming aid fenfluramine was withdrawn from the market mainly due to cardiac and pulmonary adverse reactions rather than hepatic adverse reactions, which were not reported [93]. For more details and discussions, see Lu Cha (*Camellia sinensis*) and Onshido.

### 5.13. Chi R Yun

Twenty-one Taiwanese patients used the TCM Chi R Yun (*Breynia officinalis*) and experienced liver injury [97,98,99]. There was intentional and unintentional Chi R Yum overdose in two patients [97] and acute poisonings in 19 patients, because *Breynia officinalis* was confused with a similar plant, *Securinega suffruticos*a, which is not known as hepatotoxin [98,99].

### 5.14. Chinese Herbal Mixtures

Patients can use an unnamed, unclassified herbal mixture of TCM. In addition, in some of these cases, only few herbs had been identified by their names. The lack of a single name of the herbal mixture, which covers all herbs present as ingredients, did not allow ascribing one of these cases to an existing named herbal product group.

Herbal hepatotoxicity was published for a heterogeneous group of herbal mixtures of TCM [76,77,78,100,101,102], with the United Kingdom as the most frequent country [76,77,78,100,101]. In three cases, hepatotoxicity was described following herbal TCM use, but details concerning the administered herbs were missing [76,100,101]. More information was provided in other reports [76,77,78]. One treatment consisted of *Dictamnus dasycarpus* (syn. Bai Xian Pi), *Gentiana scabra, Hedyotis diffusa*, *Paeonia suffructicosa*, *Paris polyphylla*, *Rehmannia glutinosa*, *Smilax glabra*, and *Sophora subprostrata* [77]. Another patient used *Angelica sinensis*, *Bupleurum chinese*, *Dictamnus dasycarpus*, *Paeonia suffructiosa*, *Philodendron chinese*, *Saposhnikovia divaricata*, *Shisandra chinesis*, *Shizonepeta tenuifolia*, and *Tribulus terrestris* [77]. The third patient with a fatal outcome used a mixture consisting of *Cocculus trilobus*, *Dictamnus dasycarpus*, *Eurysolen gracilis*, *Glycyrrhiza*, *Lophatherum*, *Paeonia*, *Potentilla*, and *Rehmannia glutinosa* [76]. Considering these three cases in two reports [76,77], there is some evidence that either *Dictamnus dasycarpus* or *Paeonia* species could be the toxic agent [77]. Analysis of a herbal remedy taken by another patient with ALF and unsuccessfully treated by LTX confirmed the presence of *Dictamnus dasycarpus* and supports its causal role [78]. Supportive evidence was also provided by four Korean patients with acute liver injury by *Dictamnus dasycarpus* used as a single herb [75].

The herbal mixture of TCM used by a patient in Canada led to ALF and sucessful LTX in the United States [102]. This mixture consisted of 12 herbs and included *Alisma plantago aquatica*, *Artemisia capillaris*, *Bupleurum*, *Chrysanthemum morifolium*, *Circuma*, *Gardenia jasminoidis*, *Gentiana scabra*, *Glycyrrhiza*, *Magnolia*, *Paeonia*, *Plantago asiatica*, and *Saussurea lappa*. The offending ingredient is unknown.

### 5.15. Chinese Skullcap

See Huang Qin.

### 5.16. Chuan Lian Zi

A 45-year old male patient with chronic hepatitis B virus (HBV) infection from Hong Kong was treated with the TCM Chuan Lian Zi (*Melia toosendan*) and experienced herbal liver injury [82].

### 5.17. Ci Wu Jia

The two Korean patients took the herbal TCM Ci Wu Jia (*Acanthopanax senticosus*) and developed acute liver injury with ALF requiring LTX [80].

### 5.18. Da Chai Hu Tang

In a Japanese patient, autoimmune hepatitis was triggered by the use of the TCM product Da Chai Hu Tang (syn. Dai Saiko To, TJ-8), a mixture of aqueous extracts from seven plants, *Bupleurum falcatum*, *Ginseng*, *Glycyrrhiza glabra*, *Pinellia*, *Scutellaria*, *Zingiber officinale*, and *Zizyphus jujuba* [91]. The offending agent is unknown. Da Chai Hu Tang contains the same components as the potentially hepatotoxic TCM Xiao Chai Hu Tang (syn. Sho Saiko To Syo Saiko To, Syo Xiao Hu Tang, TJ-9) but in different proportions [91,103]. See also *Bupleurum* and Xiao Chai Hu Tang.

### 5.19. Da Huang

A 45-year old man of Hong Kong with chronic HBV infection used the herbal TCM product Da Huang (Rhubarbe, *Rheum palmatum*) and died from ALF and acute multiorgan failure as a consequence of herbal hepatotoxicity [82].

### 5.20. Dai Saiko To

See Dai Chai Hu Tang.

### 5.21. Dan Zhi Xiao Yao San

See Kamishoyosan.

### 5.22. Dictamnus Dasycarpus

See Bai Xian Pi.

### 5.23. Gan Cao

A 46-year old man from Hong Kong with a chronic HBV infection took the TCM product Gan Cao (syn. *Glycyrrhiza uralensis*, Liquorice, Gan Cao Zhi, Shen Nong Ben Cao Jing, Zhi Gan Cao) in formulas, which contained eleven elements including Gan Cao as the likely toxic agent for the observed liver injury [82]. Recovery was complete after discontinuation of Gan Cao.

### 5.24. Ge Gen

Two 57- and 58-year old women from Korea ingested juice of the herbal TCM product Ge Gen (*Pueraria lobata, s*yn. Arrowroot) and developed symptomatic liver injury [104]. Clinical symptoms and laboratory findings rapidly improved following cessation of this herb and supportive care.

### 5.25. Glycyrrhiza Uralensis

See Gan Cao.

### 5.26. Ho Shou Wu

A 54-year old Korean woman took *Polygonum multiflorum* as the TCM product Ho Shou Wu (syn. He Shou Wu, Shou Wu Wan, Fo Ti) and experienced acute liver injury [105]. Hepatotoxicity of Ho Shou Wu was also assumed in a 33-year old woman in Hong Kong with a chronic HBV infection, but comedication also consisted of the hepatotoxic Jue Ming Zi (*Cassia obtusifolia tora*, Senna) and 10 additional and not further identified herbal products [82].

### 5.27. Huang Qin

Nineteen Japanese patients developed liver injury after the use of the herbal TCM Huang Qin (*Scutellaria baicalensis*, syn. Chinese skullcap), called Ogon in Kampo medicine of Japan and included in a herbal mixture [106]. Liver injury was described in four patients of the United States who used Huang Qin in a dietary supplement, which also contained black catechin (*Acacia catechin*), glucosamine, chondroitin, and hyaluronic acid [107,108,109]. *Acacia catechu* was used as one of several Indian Ayurvedic herbs in a patient with severe liver injury and is thereby a possible offending agent [89], although the herbal extract of Chinese skullcap is the more likely cause of the reported liver injuries [107,108,109]. Another 54-year old woman in the United States experienced liver injury following use of a herbal TCM mixture containing Chinese skullcap and black catechu for two to four weeks prior to admission [110].

### 5.28. Hwang Geun Cho

A 37-year old Korean male patient consumed the herbal TCM product Hwang Geun Cho (*Corydalis speciosa*) and experienced acute liver injury [111]. Symptoms disappeared and laboratory values decreased gradually to near normal values following cessation of this herbal TCM and supportive care.

### 5.29. Ji Gu Cao

A 38-year old man of Hong Kong with chronic HBV infection took the herbal TCM product Ji Guo Cao (syn. *Abrus cantoniensis*, Ji Gu Cao Wan) and developed an acute liver injury, possibly also caused by contaminating hepatotoxic seeds [82].

### 5.30. Ji Xue Cao

Three women aged 61, 52, and 49 years from Argentina ingested the herbal TCM product Ji Xue Cao (*Centella asiatica*, syn. Gotu Kola) and developed liver injury due to *Centella asiatica* [112]. Outcome was favorable after discontinuation of the herbal TCM and ursodeoxycholic acid therapy.

### 5.31. Jia Wei Xiao Yao San

See Kamishoyosan.

### 5.32. Jiguja

A 3 and a half year old boy in Korea consumed tea prepared from the herbal TCM Jiguja (*Hovenia dulcis*), resulting in the development of acute liver injury [113]. Due to a risk of ALF, the child was transferred to another hospital for further evaluation and liver transplantation. References are presented for two other cases in Korea with acute liver injury in adult patients after ingestion of *Hovenia dulcis* [79,80,113], one of the patients required a LTX [80].

### 5.33. Jin Bu Huan

Hepatotoxicity associated with the herbal TCM product Jin Bu Huan, syn. An Shu Ling [70], was reported in 11 patients in the United States [70,114,115,116] in one patient originating from Canada [115], and in one patient in Italy [117]. L-tetrahydropalmitate is the active ingredient of Jin Bu Huan and the suspected causative agent for liver injury. The herbal medication usually contains only *Lycopodium serratum* and rarely several unrelated herbal species including *Corydalis species*, *Panax ginseng*, Pseudo ginseng or two species of *Stephania* [70,114,115,116,117,118].

### 5.34. Jing Tian San Qi

Up until 2008, 41 cases in China with hepatic sinusoidal obstruction syndrome (HSOS), formerly called hepatic veno-occlusive disease (HVOD), were reported and attributed to the herbal TCM product Jing Tian San Qi (*Sedum aizoon*, syn. Stonecrop) [119]. However, the initially suspected causal role of *Sedum aizoon* was obviously incorrect. *Sedum aizoon* lacks pyrrolizidine alkaloids (PAs), and when applied to animals, HSOS did not occur [120]. This suggests that another herb containing PAs was likely responsible for the reported cases [119]. In line with this is another case in Hong Kong with HSOS that initially was ascribed also to *Sedum aizoon*, but it turned out to have been caused by the herbal TCM Shan Chi (*Gynura segetum*) [120]. *Sedum aizoon* looks like *Gynura segetum*, but differentiation is possible for botanical experts [120]. Comparative studies with both herbs provided clear supportive evidence for *Gynura segetum* as the offending herb of cases of HSOS as compared to *Sedum aizoon*. Studies showed that in mice *Gynura segetum* as the PAs containing herb but not *Sedum aizoon* lacking PAs induces HSOS as confirmed by liver histology examination [120]. In an earlier experimental study, a model of the hepatic veno-occlusive disease was established by pyrrolizidine alkaloids derived from a herb described erroneously as *Sedum aizoon* [121], which again does not contain PAs [120,122,123] This suggests that the described experimental model [121] was due to the action of a herb containing PAs, most likely *Gynura segetum* [120,122,123], rather than to *Sedum aizoon*. Based on these well founded conclusions, evidence for a hepatotoxic potential of Jing Tian San Qi is lacking. This herbal TCM *Sedum aizoon* should therefore not further be considered as hepatotoxic herbal TCM.

### 5.35. Ju San Qi

In two Chinese women, HSOS was induced by pyrrolizidine alkaloids of the herbal TCM *Gynura segetum* (syn. Ju Shan Qi, Ju Ye San Qi, Shan Chi, San Qi Cao, Shan Chi, Shan Chi) [124]. Additional six cases were earlier suspected [125,126], in at least four cases the culprit was the PAs containing herb *Heliotropium lasiocarpum* rather than *Gynura segetum* [127]. Two cases of HSOS were reported in China [128,129] and another one in Hong Kong [120].

### 5.36. Jue Ming Zi

In a 33-year old woman of Hong Kong with a chronic HBV infection was treated with the herbal TCM product Jue Ming Zi (syn. *Cassia obtusifolia*, *Senna obtusifolia*, Cao Jue Ming) and experienced liver injury [82].

### 5.37. Kamishoyosan

In one single Japanese woman, liver injury was reported following the use of Kamishoyosan, a traditional Japanese herbal drug (Kampo medicine) and synonym to the TCM product Jia Wei Xiao Yao San, Dan Zhi Xiao Yao San or TJ-24 [92]. Kamishoyosan is a herbal mixture and contains several components like *Angelica sinensis*, *Atractylodes racea*, *Bupleurum falcatum*, *Gardenia*, *Glycyrrhiza glabra*, *Mentha haplocalyx*, *Moutan*, *Paeonia alba*, *Sclerotium Poriae Cocos* and *Zingiber officinale*, as described in the case report [92], or assessed by an additional internet search for a refined botanical description of the herbal components. The identification of the causative agents was difficult, but *Scutellaria* was definitively excluded as it was not an ingredient of this herbal product [105,130]. Some uncertainty exists around the *Mentha species*, declared as *Mentha* herb in the case report [92] and as mentha (pennyroyal) subsequently [130]. Based on an internet search to further specify the *Mentha* species commonly used in Kamishoyosan, *Mentha haplocalyx* Briq or *Mentha arvensis* var. *piperascens* Malinvaud (Japanese field mint) was most probably the main component.

### 5.38. Kudzu

Six patients in Korea consumed the herbal TCM product Kudzu (*Pueraria thunbergiana*) and developed acute liver injury [79].

### 5.39. Liquorice

See Gan Cao.

### 5.40. Long Dan Xie Gan Tang

A total of 14 Taiwan patients with acute and subacute HBV infection were allegedly reported at high risk of hepatotoxicity when treated with the TCM Long Dan Xie Gan Tang (syn. Long Dan Xie Gan Wan) [90]. This herbal mixture contains *Acebia*, *Alisma*, *Angelica sinensis*, *Bupleurum*, *Gardenia*, *Gentiana*, *Glycyrrhiza*, *Plantago*, *Rehmannia* and *Scutellaria*. A similar increased risk of hepatotoxicity was observed in a group of patients treated with the TCM Xiao Chai Hu Tang, which also contains *Bupleurum* among other herbs. See also *Bupleurum* and Xiao Chai Hu Tang.

### 5.41. Long Dan Xie Gan Wan

See Long Dan Xie Gan Tang.

### 5.42. Lu Cha

Lu Cha (*Camellia sinensis*, green tea) is a plant of the TCM and one of several herbal ingredients of the two herbal mixtures Chaso and Onshido, which were marketed as weight loss aids by Chinese pharmaceutical companies and considered hepatotoxic as described in 2003 [93]. *N*-nitroso-fenfluramine but not green tea was discussed as the possible but unproven hepatotoxic ingredient for these herbal mixtures, explaining that this case series cannot prove that *N*-nitroso-fenfluramine is the toxic agent [93]. Whether *Camellia sinesis* might have contributed to the observed liver injury is unclear, however. Information on the amounts of green tea in these two products was not provided. At least as extract, *Camellia sinensis* is a potent weight loss aid with potentially hepatotoxic effects, as thoroughly discussed first in 2004 [131,132,133] and in subsequent years as shown for many cases [134,135,136,137,138,139,140,141,142,143,144]. Therefore, hepatotoxicity of green tea as extracts was not yet clearly established in 2003 when the case reports were published [93].

Green tea is one of the most popular beverages, as are black tea and coffee. There is no question that the conventional use of these beverages including green tea does not harm the liver. In the past, however, weight loss aids were supplemented by green tea concentrated as extracts and carried the risk of liver injury [60,134,135,136,137,138,139,140,141,142,143,144,180]. According to the manufacturers, the weight loss aids Chaso and Onshido contained green tea and other herbs [93]. Presumably, green tea was included as extracts in these two products to enhance weight loss, although the extract form was not specifically mentioned by the manufacturers. The producers failed to mention the synthetic adulterant *N*-nitroso-fenfluramine as an ingredient of the two products to promote weight loss. This adulterant was assessed later on by respective chemical analyses, but evidence for its hepatotoxic property was not presented and asked for by future studies [93]. The most likely candidate for the liver injury is green tea, if supplied as the hepatotoxic extract. For details, see also Chaso and Onshido.

Liver injury due to green tea extracts (GTE) has been reported as single cases [131,132,133,134,135,142], case series [136,137,138,139,141,144], and discussed in review articles [60,139,140,141,180]. Overall, more than 100 suspected cases of liver injury by GTE have been published [60,180], while details of other cases are provided by the NIH liver Tox [47].

### 5.43. Ma Huang

Six patients in the United States experienced acute liver injury associated with the use of the herbal TCM product Ma Huang and its ingredients *Ephedra* [74,85,145,146,147,148]. Out of these six cases, one patient received a coadministration of kava and another one of disulfiram, whereas a third one had chronic HBV infection [74]. Two patients developed ALF [74,146], and one of these required a liver transplant [74]. Another patient with ALF requiring a LTX was reported in the United Kingdom [149]. Ma Huang is also one of the ingredients of Pro-Lean^®^, a potentially hepatotoxic herbal mixture [150]. For *Ephedra*, see also Bofu Tsu Sho San.

### 5.44. Mao Guo Tian Jie Cai

Four patients from Hong Kong developed HSOS following the use of the herbal TCM product Mao Guo Tian Jie Cai (*Heliotropium lasiocarpum*) [127] that contained PAs, initially mistaken as PAs containing herbal TCM *Gynura segetum* [124,125,126]. Outcome was fatal for one patient [125].

### 5.45. Onshido

Six Japanese patients presented with hepatic injury due to the use of the weight loss aid Onshido, a TCM herbal mixture [93]. This herbal product contained *Aloe*, *Camellia sinensis*, *Crataegus*, *Gynostemma pentaphyllum makino* and *Raphanus*. Outcome was favorable in all patients except for one patient with a fatal clinical course. Chemical product analysis of Onshido showed lack of fenfluramine and heavy metals such as copper, lead, bismuth, cadmium, stibium, stanum, mercury, and chromium, but presence of *N*-nitroso-fenfluramine as a possible but overall unproven hepatotoxic agent. *N*-nitroso-fenfluramine was also considered as a possible culprit in additional cases of liver injuries due to various other slimming aids in the United Kingdom [94], Hong Kong [95], and Japan concerning the TCM product Chaso [93]. An additional 135 Onshido-induced cases of hepatotoxicity were reported to the regulatory agency in Japan but not further analyzed [93]. A possible role of *Camellia sinensis* for the observed hepatotoxicity of Onshido was not discussed in 2003 [93], because hepatotoxic properties of *Camellia sinensis* extracts became only known in 2004 [132,133,134]. For additional details and discussions, see also Chaso and Lu Cha (*Camellia sinensis*).

### 5.46. Phyllanthus Urinaria

See Zhen Chu Cao.

### 5.47. Polygonum Multiflorum

*Polygonum multiflorum* is a member of the family Polygonaceae, genus *Fallopia*. Either alone or combined with other herbs and vitamins, it is a component of various potentially hepatotoxic herbal TCM products [80,81,82,105,150,151,152,153,154]. Among these are Ban Tu Wan [81], Ho Wu Shou [82,105], Shen Min [151] and Shou Wu Pian [152,153,154,155,156,157,158,159]. Occasionally, *Polygonum multiflorum* containing herbal TCM products such as Ho Shou Wu, Shen Min, Shou Wu Pian and Zhi Shou Wu are declared interchangeable regarding their terms [150]. However, ingredients may vary from product to product, requiring specific and qualifying product names. The mechanism of hepatotoxicity of *Polygonum multiflorum* is still disputed [150,151,152,155,156,157]; the injury was sometimes attributed to the anthraquinones (such as chrysophanol, emodin and rhein) which are the major ingredients of *Polygonum multiflorum* [150]. In a single report, however, the major compound identified in the recovered tablets was a stilbene glycoside, tetrahydroxystilbene-glucopyranoside [150,152]. LTX was necessary in three patients in Korea after using *Polygonum multiflorum* [80]. A 33-year old woman from Slovakia experienced acute liver injury after use of *Polygonum multiflorum* for two months [160]. Several review articles and analyzing reports of case series of suspected liver injury by *Polygonum multiflorum* were published [161,162,163,164]. For instance, a CFDA report analyzed retrospectively 24 literature cases of HILI by *Polygonum multiflorum* and published its results in the Chinese language without proving causality by a liver specific causality assessment method [161] such as RUCAM [51]. Instead, another Chinese study described valid clinical characteristics of HILI by *Polygonum multiflorum* in 18 cases with “highly probable” or “probable” causal role of this TCM herb using RUCAM criteria, all alternative infections including hepatitis E virus (HEV) were excluded; this is really a breakthrough study that is highly appreciated and should be used as an example for future studies on suspected HILI by TCM [162]. Similarly, other studies on liver injury cases confirmed causality for *Polygonum multiflorum,* based on high RUCAM gradings and results derived from positive reexposure tests [60,180], using published test criteria [51]. In another systematic review of case reports and case series, 450 cases of liver injury due to *Polygonum multiflorum* were analyzed regarding symptoms, outcome, and risk factors, and other criteria; however, reported results were not convincing as causality was not ascertained by any liver specific method [163] such as RUCAM [51]. Finally, causality in 187 cases of suspected liver injury by *Polygonum multiflorum* was confirmed following assessment using RUCAM [164]. For details see also Ban Tu Wan, Chinese herbal mixtures, Ho Wu Shou, Shen Min and Shou Wu Pian.

### 5.48. Qibao Meiran Wan

A 26-year-old Chinese man experienced weakness, fatigue, poor appetite, dark urine, and jaundice after using for one month the TCM product Qibao Meiran Wan A for greying of hair [165]. The product label listed components that included *Polygonum multiflorum*, *Angelica sinensis*, *fructus psoraleae* (Yan Shuizhi), wolfberry fruit, dodder, *poria cocos*, and *achyranthes bidentata*.

### 5.49. Rhen Shen

Six patients originating from Korea used the herbal TCM Rhen Shen (*Panax ginseng*, Ren Seng) and developed acute liver injury [79].

### 5.50. Sairei To

Two Japanese men consumed the Kampo medicine Sarie To (syn. Chai Ling Tang), which is included in a blend of two TCM products, Xiao Chai Hu Tang and Wu Ling San Wan, and experienced liver injury [166,167]. Among the Sairei To ingredients are *Alisma*, *Atractylis*, *Bupleurum*, *Cinnamomum*, *Ginseng*, *Glycyrrhiza*, *Pinellia*, *Polyporus*, *Poria*, *Scutellaria*, *Zingiber* and *Zizyphus*. Several possible offending agents are considered, including *Pinellia ternate* [166] and other components of Sairei To [167].

### 5.51. Shan Chi

In two 51-year and 39-year-old Chinese women, HSOS was reported as induced by PAs of the herbal TCM Shan Chi *Gynura segetum* (syn. Ju San Qi, Ju Shan Qi, Ju Ye San Qi, Shan Chi, San Qi Cao, Shan Chi), Tu San Qi) [124]. One of these patients required a liver transplant. In a 54-year-old woman originating from Hong Kong, HSOS was finally attributed to PAs derived from *Gynura segetum* rather than to the herbal TCM Jing Tian San Qi (*Sedum aizoon*) devoid of PAs [120]. An additional four patients experienced HSOS after *Gynura segetum* consumption, one with fatal outcome [122]. In total, at least 51 HSOS cases have been reported until 2011 [120], and 116 cases until 2012 [122].

The clinical features have been clearly described, establishing *Gynura segetum* but not Jing Tian San Qi (*Sedum aizoon*) as the cause for HSOS [120,122,123]. The diagnosis was ascertained in the 54-year-old female patient with HSOS by thorough investigations of the patient and in animal studies [120]. The clinical diagnosis of HSOS was firmly established by meeting the modified Seattle criteria, characterized by hyperbilirubinaemia, hepatomegaly, and weight gain due to fluid accumulation. Liver histology indicated that the diagnosis was HSOS, and pyrrole-protein adducts as well as pyrrole-GSH conjugates were found in the blood and established the diagnosis. Since the ingested herb was unknown, the cultivated herb from the patient’s home was collected and authenticated as the TCM herb *Gynura segetum*. Together with the authenticated TCM herb *Sedum aizoon* cultivated and collected from another Chinese area, various comparative studies in animals have been done. All these studies confirmed that the observed HSOS arose from the consumption of the PA-containing *Gynura segetum*, an erroneous substitute of the Sedum aizoon, which does not contain PAs and has a similar name [120].

### 5.52. Shen Min

A 28-year-old woman originating from the United States developed in Columbia acute liver injury following the use of the herbal TCM product Shen Min [151]. The product label for this Shen Min product listed components including plants and vitamins. *Polygonum multiflorum* is one of the main components of Shen Min, the content is described as Shen Min 12:1 standardized extract (*Polygonum multiflorum*) 450 mg per serving and as He Shou Wu powder 870 mg per serving, although *Polygonum multiflorum* was not specified. Other declared components of the used Shen Min product were Vitamin A, Vitamin B_6_, biotin, niacin, pantothenic acid, soy isoflavones, black cohosh, horse chestnut, hydrolyzed collagen, silica from plant sources, *ginkgo biloba*, *uva ursi*, Burdock, Cayenne pepper and *Piper nigrum*. See also Ho Shou Wu, *Polygonum multiflorum* and Shou Wu Pian.

### 5.53. Shi Can

Seven patients in France experienced acute liver injury due to use of Shi Can, in Western countries better known as germander (*Teucrium chamaedrys, Teucrium polium*), for three to 18 weeks [168]. After cessation of the herb, recovery was complete in all patients. In three of these, unintentional reexposure led to prompt recurrence of the liver injury. Germander was proposed for weight control in the late 1980s in France, leading to a spectacular outbreak of acute liver injury within a year. This initial report [168] triggered a national inquiry in France leading to the collection of 26 well-documented cases of acute liver injury due to Germander [169]. Liver injury was mainly mild to moderate, occurring about two months after starting the treatment [168,169].

Subsequently, additional cases of liver injury due to germander were reported including two French patients with a fulminant clinical course [170,171], one with a lethal outcome [170]. In a few patients, liver damage occurred more insidiously and was detected at the stage of chronic hepatitis and even cirrhosis [172,173], due to long use of six to seven months [171] or large daily amounts of several liters of herbal tea for two months [172]. In 12 patients accidentally reexposed to germander, liver injury relapsed within a delay shorter than for the first episode of hepatitis [169]. None of the cases had been submitted at that time to causality assessment using RUCAM or criteria of positive reexposure tests, as both were not available when the reports were published [168,169,170,171,172,173]. There are additional case reports that provide informative narratives [174]. For *Teucrium polium*, another herbal medicine closely related to *Teucrium chamaedrys*, rare cases of acute liver injury have been reported including acute liver failure (ALF) requiring liver transplantation (LTX) [175,176]. As a consequence, Germander has been withdrawn from the market of herbal medicine in France. However, since this outbreak, it is still used in some other countries and new cases have been observed in Canada, Belgium and Spain [174].

### 5.54. Shou Wu Pian

As described already in 1996, a 31-year-old pregnant Chinese woman from Hong Kong developed acute liver injury after consumption of the TCM product Shou Wu Pian, with *Polygonum multiflorum* as the main component [155]. Subsequently, similar cases related to Shou Wu Pian were reported in Australia [156], Canada [116], Italy [154,157], and the Netherlands in a 5-year-old girl [152], the United States [158], Japan [159], and Korea [153]. Shou Wu Pian is a herbal mixture with a wide variety of ingredients, but details are rarely mentioned [150,153,159]. For example, according to the information via the internet, one of the numerous possible compositions could be: *Achyranthes bidentata*, *Cuscuta chinensis*, *Eclipta prostrata*, *Ligustrum lucidum*, *Lonicera japonica*, *Morus alba*, *Polygonum multiflorum*, *Psoralea corylifolia*, *Rehmannia glutinosa*, *Rosa laevigata*, *Sesemum indicum,* and *Siegesbeckia orientalis*. This composition varies substantially from the one of Shen Min [151], which also contains *Polygonum multiflorum* and is discussed above. See also Ho Shou Wu, *Polygonum multiflorum* and Shen Min.

### 5.55. Syo Saiko To

See Xiao Chai Hu Tang.

### 5.56. TJ-8

See Da Chai Hu Tang.

### 5.57. TJ-9

See Xiao Chai Hu Tang.

### 5.58. TJ-24

See Kamishoyosan.

### 5.59. White Flood

A 23-year-old man from the United States experienced acute liver injury following use of the commercial product White flood, which contains among others the herbal TCM Wu Zhu Yu (*Evodia rutaecarpa*, club moss) and the herbal TCM Qian Ceng Ta (*Huperzia serrata*) [177]. Other ingredients included acesulfame potassium, beet root, caffein, calcium silicate, carnitine tartrate, Carno-Syn^®^ beta-alanine, citrulline, cocoa bean, cryptoxanthin, folic acid, gamma-aminobutyric acid (GABA), glucuronolactone, selenium, l-norvaline, l-tyrosine, lutein, malic acid, ornithine, potassium gluconate, sucralose, sugar cane, vinpocetine (from *Vinca* plant), water melon flavor, and zeaxanthin. After discontinuation of the product use, the outcome was favorable.

### 5.60. Xiao Chai Hu Tang

The treatment with the herbal TCM Xiao Chai Hu Tang (syn. Sho Saiko To, Syo Saiko To, Syo Xiao Hu Tang, TJ-9) resulted in liver injury in four Japanese patients with LT abnormalities before treatment [178]. For 19 Taiwan patients with HBV infection, treatment with Xiao Chai Hu Tang was associated with an increased risk of liver injury [90], as was for one additional patient of Taiwan following cholecystectomy [179]. Xiao Chai Hu Tang is a mixture of several herbs, *Bupleurum falcatum, Ginseng*, *Glycyrrhiza glabra*, *Pinellia tuber, Scutellaria baicalensis, Zingiber officinale and Zizyphus jujuba*. It therefore contains the same components as the TCM Da Chai Hu Tang (syn. Dai Saiko To, TJ-8), but in different proportions [91,103]. See also *Bupleurum*, Da Chai Hu Tang and Long Dan Xie Gan Tang.

### 5.61. Yin Chen Hao

In seven patients from Korea, the use of the herbal TCM Yin Chen Hao (*Artemisia capillaris*) resulted in the development of acute liver injury [79], and one additional Korean patient required a LTX [80].

### 5.62. Zexie

A 59-year old man from Hong Kong with cirrhosis and HBeAg-positive chronic HBV infection was treated for cramps with a herbal TCM formula of 11 different herbal elements including the hepatotoxic Zexie (*Alisma orientalis*) [82]. He died from complications of severe course of herbal liver injury.

### 5.63. Zhen Chu Cao

A 37-year-old man from Hong Kong with a chronic HBV infection experienced acute liver injury due to the TCM product Zhen Chu Cao experienced herbal hepatotoxicity due to Zhen Chu Cao (syn. *Phyllanthus urinaria*) [82]. After cessation of this herbal product, there was a complete recovery.

### 5.64. Zhi Gan Cao

See Gan Cao.

## 6. Pathogenetic Aspects of Liver Injury from Herbal TCM

The pathogenetic events whereby TCM herbs cause liver injury are mostly unknown, as herbal liver injury is primarily a human and not an animal disease; experimental models to study in detail the mechanisms leading to injury are therefore rarely available. Due to their complexity and as expected, valid considerations on pathogenetic sequelae of liver injury due to herbal TCM were provided only for selected herbs, both in reports with a focus on narratives of case reports or small case series [70,71,72,73,74,75,76,77,78,79,80,81,82,83,84,85,86,87,88,89,90,91,92,93,94,95,96,97,98,99,100,101,102,103,104,105,106,107,108,109,110,111,112,113,114,115,116,117,118,119,120,121,122,123,124,125,126,127,128,129,130,131,132,133,134,135,136,137,138,139,140,141,142,143,144,145,146,147,148,149,150,151,152,153,154,155,156,157,158,159,160,161,162,163,164,165,166,167,168,169,170,171,172,173,174,175,176,177,178,179] or in articles covering or reviewing larger case series [181,182,184,185,186,187,188,189,190].

Most processes will occur at the molecular level, considering the molecules of the phytochemicals and the molecules of enzymes responsible for metabolic alterations of these phytochemicals in the liver.

### 6.1. Abundance of Herbs Used as TCM

Herbs grow at various extents in most countries of the world and provide the basis of local traditional herbal medicine in their respective cultures [191], including China [12,69], a country rich in plants [6,12,28,41,55,57,67,68,69], where around 13,000 herbal preparations are used as TCM [67,68,69].

### 6.2. TCM Herbs and Their Molecules

In analogy with the other herbs, TCM herbs contain multiple molecules, which require elimination in order to circumvent hazardous accumulation [25,59,140]. Therefore, these phytochemicals must undergo enzymatic degradation and disposition following intestinal resorption by processes similar to those known for synthetic drugs, involving bioactivation pathways via the cytochrome P450 (CYP) systems (Phase I), conjugation to other molecules (Phase II), and excretion and transport (Phase III) [140,192,193,194,195,196,197,198]. Examples for such metabolic steps are provided for many TCM herbs [194,195] such as *Polygonum multiflorum* [196], Lu Cha (green tea) [140], Shi Can (germander) [199,200,201], and PAs containing herbs [28,120,122,201]. Genetic variation may cause polymorphisms of CYP [192,193], which has recently been reported also in Chinese patients with acute liver injury by *Polygonum multiflorum* [196], who showed a 46.5% higher frequency of CYP1A2*1C allele as compared to 27.9% in the healthy control group. Thus, patients with this mutation may have less CYP1A2 protein, thereby inhibiting degradation of *Polygonum multiflorum* ingredients and causing accumulation of toxic substances [196].

Other molecular risk factors of liver injury relate to the extraction process used in herbal TCM manufacturing, which is conventionally based on decoction and alcohol sedimentation techniques [194]. Besides the water soluble components, many liposoluble constituents are included in the final herbal TCM preparations and require biotransformation. Lipophilicity of synthetic drugs is under discussion as a risk factor of DILI [192,198,202,203] and may have to be evaluated for liver injury by TCM herbs as well. Results of other DILI studies related to extensive metabolism and high daily doses as risk factors of DILI [192,204,205,206] could also be of relevance for TCM herbs. For instance, drugs intensively metabolized by the liver have a higher likelihood causing DILI [192,205,206], and drugs metabolized by CYP enzymes were four times as likely to cause DILI [192,203]. In more detail, drugs metabolized by CYP1A2, CYP2C8/CYP2C9, and CYP3A5 were closely associated with an increased risk of DILI [203]. Drugs given in a high daily dose of >100 mg were nearly five times as likely to cause DILI a lower daily doses [192,203]. CYPs usually detoxify various chemicals from TCM herbs but some are activated to toxic compounds which may initiate liver injury [59].

### 6.3. Idiosyncratic and Intrinsic Liver Injury from Herbal TCM

Liver injury caused from herbal TCM is rare and in most cases unpredictable; defined as the idiosyncratic type of HILI. Instead, few TCM herbs cause predictable liver injury; defined as the intrinsic type of HILI [50,60]. Manufacturing and selling of TCM herbs with the potential for intrinsic liver injury is unethical. In countries with strict regulatory surveillance, herbal TCM products that cause such type of liver injury should be withdrawn from the market. Several criteria allow differentiation of the idiosyncratic from the intrinsic liver injury by TCM [50,60].

#### 6.3.1. Idiosyncratic Type of Liver Injury

**Figure 3:**
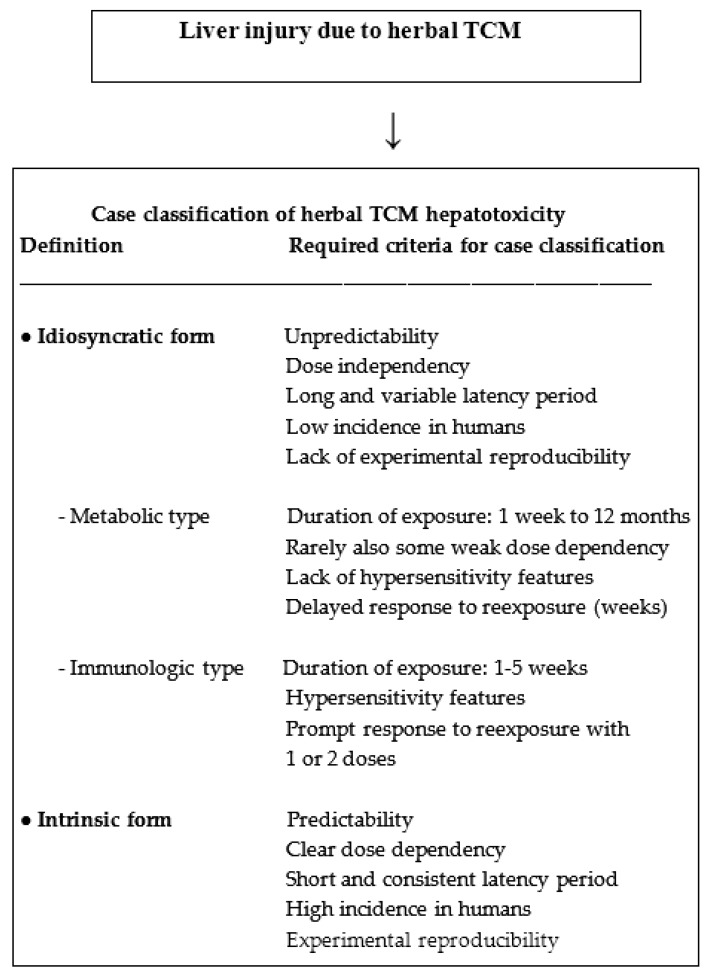
Case classification of liver injury due to herbal TCM, adapted from previous reports [50,60].

Idiosyncratic liver injuries due to TCM herbs are not preventable as they manifest in a few susceptible individuals with a likely genetic basis, which is commonly not known prior to the use of TCM herbs [50,60]. Idiosyncrasy explains also many liver injury cases such as by synthetic drugs [51,192,197,198,207] and non TCM herbs [50,60,208,209], including kava [210,211,212,213], *Chelidonium majus* [214,215,216], and Indian Ayurvedic herbs [89]. There is little if any information of the various steps which finally lead to idiosyncratic liver injury in the human liver cell. Case classification of idiosyncratic liver injury due to herbal TCM requires the fulfillment of several criteria [50,51,61]. In addition to unpredictability, dose independency is another major criterion for idiosyncratic liver injury that is further differentiated by its two subtypes, a metabolic type and an immunologic type, all with specific criteria (Figure 3).

Most problems of evolving liver injury will occur more likely during the metabolic degradation of the phytochemicals due to the genetic variability of the affected individuals rather than during the excretion of the chemical compounds. It is unknown to what extent interactions among the multiple herbal chemicals derived from herbal TCM mixtures are causative for the liver injury. In most TCM herbs, the chemicals which initiate liver injury are not identified. In accordance with other idiosyncratic liver injury cases, idiosyncratic HILI due to TCM in humans develops at recommended doses and is not reproducible in experimental animals, which do not exhibit the genetic variability or the environment of patients who experience idiosyncratic liver injury. Consequently, the pathophysiology of idiosyncratic liver injury from herbal TCM in humans remains undisclosed. 

#### 6.3.2. Intrinsic Type of Liver Injury

Intrinsic liver injury due to herbal TCM is predictable and hence preventable, shows a clear dose dependency with increased risks at high doses, and is characterized by a high incidence in individuals who consume these risky herbs (Figure 3) [50,60]. Intrinsic liver injury is also known for synthetic drugs if used in overdose such as acetaminophen [192]. Cases of intrinsic liver injury caused by TCM herbs are well studied due to reproducibility in laboratory animals (Figure 3).

Among the first TCM herbs causing intrinsic liver injury was the TCM Shi Can, which consists of *Teucrium chamaedrys* or other *Teucrium* species and is better known in Western countries as Germander [201]. Germander hepatotoxicity fulfills all criteria of intrinsic liver injury (Figure 3), is dose dependent, well described, and reproducible in mice [199,201]. Due to its experimental reproducibility in laboratory animals, the molecular pathogenesis of experimental Germander (*Teucrium chamaedrys*) hepatotoxicity was studied, and these results are well transferrable to human Germander hepatotoxicity [199,201]. Neoclerodane diterpenoids are ingredients of Germander and converted via microsomal CYP3A to reactive metabolites [199]. These deplete hepatic stores of glutathione and cytoskeleton associated protein thiols, form plasma membrane blebs, and cause apoptosis of liver cells [129,201], contributing to liver cell death [201]. Finally, reactive metabolites can trigger liver injury through an immunoallergic reaction.

There is a wide range of other TCM herbs that may cause intrinsic liver injury. Among these are herbal TCM products such as Cai Hu, Da Chai Hu Tang, Kamishoyosan, Long Dan Xie Gan Tang, Sairei To, or Xiao Chai Hu Tang that all contain *Bupleurum falcatum,* mostly prepared from its radix [90,217]; using more than 19 g as cumulative dose of *Radix bupleuri* may increase the risk of liver injury [90]. This dose dependency was confirmed in laboratory animals and provided insights into some pathogenetic processes [217].

*Polygonum multiflorum* is another example of a typical TCM with potential liver injury [218,219], as shown in many case reports and case series, where this herb was used either alone or as one among other ingredients in herbal mixtures (Table 4) [80,81,82,105,150,151,152,153,154,155,156,157,158,159,160,161,162,163,164]. These include Bai Shi Wan, Bi Ma Zi, He Shou Wu, Ho Shou Wu, Qibao Meiran Wan, or Shen Min. Human liver injury by *Polygonum multiflorum* is reproducible in laboratory animals and suggests an intrinsic type of injury [163]. After analysis of the relevant publications, the impression prevails that most cases of human *Polygonum multiflorum* hepatotoxicity seem to represent the intrinsic liver injury [80,81,82,105,150,151,152,153,154,155,156,157,158,159,160,161,162,163,164]. However, one study favored an idiosyncratic type of injury in patients who used the herb alone and not as part of a herbal mixture, but patients who took a herbal mixture containing *Polygonum multiflorum* were exluded and not evaluated [162]. Assessing the type of liver injury (Figure 3) is impeded by confounding variables [163]. Among these are incorrect reporting [163], comedication [162], preexisting infections by viral hepatitis [162], water or acetone based products [162,163,219], and the use of raw or processed *Polygonum multiflorum*, whereby processing is achieved by boiling of the reddish roots with black beans [162,163,218]. Consensus exists that both raw and processed *Polygonum multiflorum* can cause liver injury in humans [165,166]. For raw *Polygonum multiflorum* applied to mice, the liver injury of water decocta was considerably more pronounced than that of the acetone extract; meanwhile, the hepatotoxicity of the acetone extract of raw *Polygonum multiflorum* was much higher as compared to the acetone extract of the processed *Polygonum multiflorum* [218].

Therefore, processing reduced experimental liver injury. Accordingly, the CMM recommended a daily dose of 3–6 g for the raw *Polygonum multiflorum* and 6–12 g for the processed product [163,219]. With a median time of 30 days, liver injury occurs when the maximum daily dose exceeds 12 g, with an increased cumulative dose as an additional risk factor [163].

The herbal TCM Lu Cha (Chinese green tea) as extract may cause intrinsic liver injury as it occurs dose dependently with high amounts of catechins provided by the extracts, as shown in various reports [132,133,134,135,136,137,138,139,140,141,142,143,144], but human liver injury was not corroborated in a recent study [220]. Results of experimental hepatotoxicity in animals were equivocal [221,222,223]. Chinese green tea is a popular beverage prepared from the leaves of the plant *Camellia sinensis*, which has been cultivated in China and other Southeast Asian countries since thousands of years [224]. Contradictory results of liver injury obtained in humans and laboratory animals may be explained by the variability of oral bioavailability of GT catechins. Consumed with a meal, systemic catechin levels in humans are much lower than the effective concentrations determined in in vitro systems; they are enhanced substantially by >3.5-fold when green tea is consumed after an overnight fast [225]. Therefore, the fasting/fed status is an additional confounding variable to be considered in bioavailability and liver injury evaluation studies of catechins.

Despite few side effects, the consumption of green tea has been considered relatively safe for most individuals when used in normal amounts [140,144,226]. On a quantitative basis, single doses of up to 1.6 grams of green tea extract are well tolerated [140,144]. The maximum tolerated dose in humans is reported to be 9.9 grams per day, a dose equivalent to 24 cups of green tea. The safety and tolerability of long term use of green tea extracts has not been well defined [144]. Considering the results of the hepatotoxicity cases [144,227,228], use of green tea or its extracts commonly ranged from four to 260 weeks [139]. However, data of cumulative doses of catechins are not available which would allow calculating respective threshold values.

TCM herbs containing unsaturated PAs cause intrinsic liver injury [201,229,230], as evidenced by dose dependency [28,119,120,121,122,123,124,125,126,127,128] and reproducibility in animals [121]. Among these herbs are *Crotalaria species* (Bush tea, Rattlebox), Gynura segetum, *Ilex paraguarensis* (Mate tea), *Symphytum* species (Comfrey), *Senecio* species (Groundsel), *Heliotropium* species, and *Compositae* species (Indian herbs) (Table 4), causing HSOS as a specific form of liver damage, injuring preferentially the hepatic sinusoidal cells rather than the liver cells. The pathogenesis of liver injury by TCM herbs containing PAs has been studied in detail [25,28,120,122,201,229,230], suggesting the involvement of hepatic microsomal CYP3A and 2B in *C*-oxidation and *N*-oxidation of the necine base to form the reactive pyrrolic ester metabolites and pyrrolizidine alkaloid *N*-oxides, respectively [25]. These metabolites damage preferentially the sinusoidal endothelial cells of the liver and reduce thereby the sinusoidal blood flow [28]. This explains the typical clinical features of HSOS, caused for instance by *Gynura segetum* [122]. However, a conceptional gap is evident due to the incompatibility of the locations between the cells where toxic PA metabolites are generated, namely in the liver parenchymal cell, and the offending target cell, namely the sinusoidal endothelial cells, which became injured. Indeed, little clinical or experimental evidence exists that hepatocytes are more affected than sinusoidal endothelial cells. HSOS due to unsaturated PAs is clearly dose dependent, thereby predictable, and hence preventable. Consequently, every consumer of these herbs containing PAs is at a dose dependent risk of HSOS. Plants containing PAs are among the most abundant poisonous plants affecting not only humans but also livestock and wildlife, with more than 6000 plant species containing PAs and about 3% of the world’s flowering plants containing PAs [229]. Human embryotoxicity by PAs resulting in fetal HSOS has been described in a newborn whose mother drank one cup of a tea containing PAs per day throughout pregnancy [229,231]. In the past, the CMM listed for human use many TCM herbs with toxic PAs [230]. All herbal TCM preparations, other herbal products as well as herbal drugs destined for human use and to ensure consumer safety must fulfill regulatory limits of PA content, which may be different from one country to the other [232]. Efficient herbal drugs can be freed from PAs through CO_2_ extraction [233,234,235].

#### 6.3.3. Tentative Toxic Components or Intermediates

Many reports suggested chemical ingredients as causes for various cases of liver injury due to TCM, but these suggestions often remain speculative for human idiosyncratic liver injury due to herbal TCM. For some TCM herbs known to cause liver injury, toxic substances have been proposed and are listed as examples (Table 5).

Few TCM herbs merit further consideration. Among these is Shi Can (germander) with its complex chemical composition [199,200]. It mainly contains neoclerodane diterpenoids, with a chemical structure similar to that of other furan compounds known to be hepatotoxic. Germander components have been shown to be mainly oxidized by CYP3A into reactive metabolites able to deplete glutathione and cytoskeleton-associated protein thiols and cause plasma membrane blebs. Another example is the liver injury caused by *Polygonum multiflorum* that is not dependent on the content of anthranoid derivatives as previously suggested but may be correlated with the content of tetrahydroxystilbene glucosides [218], in support of other studies [150,152]. Finally, Lu Cha (green tea) extracts cause liver injury, but it remains unclear which of their polyphenolic catechins is the causative agent [141]. Candidates are epigallocatechin-3-gallate (EGCG), epicatechin-3-gallate (ECG), epicatechin (EC), epigallocatechin (EGC), and epigallocatechin-3-catechins (EGCG) [140,141]. Of these catechins, particularly EGCG was found to be cytotoxic in isolated hepatocytes by mitochondrial membrane potential collapse and reactive oxygen species’ formation [141,222]. EGCG is also the most abundant catechin in the green tea extracts [140,141]. The earliest Chinese medical classic “The Inner Canon of Huangdi”classifies TCM herbs as having high, moderate, or low toxicity, or as being nontoxic; in addition, recent toxicological studies found that some chemical ingredients of the TCM herbs classified as nontoxic can cause liver injury [181]. Likely in order to reduce toxicity of some TCM herbs, TCM philosophy requires the use of numerous herbal TCM products as mixtures consisting of different herbs, commonly with up to six herbs [28,185] or even more (Table 4) [185]. Typically, there is a primary herb, referred to as the “King” [28] or “Monarch” [67] herb as the key ingredient. The other constituents, called also “Minister”, “Assistant”, or “Envoy” [67], are believed to function as modifiers of toxicity, which is well recognized but not detailed and not described in terms of specific molecular toxins [28,67]; they are also considered to synergistically increase the King herb effects [185], to improve the immune function [28], or strengthen certain aspects of not further described actions [28].

## 7. Key Clinical Features of Liver Injury Due to TCM Herbs

Clinical features are well described in many liver injury cases, but not all reports provide sufficient evidence of a causal relationship to the suspected TCM herb [70,71,72,73,74,75,76,77,78,79,80,81,82,83,84,85,86,87,88,89,90,91,92,93,94,95,96,97,98,99,100,101,102,103,104,105,106,107,108,109,110,111,112,113,114,115,116,117,118,119,120,121,122,123,124,125,126,127,128,129,130,131,132,133,134,135,136,137,138,139,140,141,142,143,144,145,146,147,148,149,150,151,152,153,154,155,156,157,158,159,160,161,162,163,164,165,166,167,168,169,170,171,172,173,174,175,176,177,178,179,181,182,184,185,186,187,188,189,190], lacking a rigorous a method causality assessment, using a method such as RUCAM [51]. Due to these uncertainties, clinical features presented below as examples and derived preferentially from case series remain in part tentative.

For clinical features of liver injury caused from herbal TCM, differences are obvious between unsaturated PA containing herbs causing HSOS and those lacking these hepatotoxins and causing liver injury with other lesions. Therefore, results of the two groups will be presented separately, for the HSOS group and the non HSOS group.

### 7.1. Duration of Herbal Use and Time to Onset

For patients with HSOS caused by *Gynura segetum*, the TCM also known as Shan Chi, Tu San Qi, or under various other names, the duration of herb intake was 4–730 days with a mean of 70.2 days and a median of 26.0 days; the latency period between start of herb use and symptoms was 5–730 days, with a mean of 88.8 days and a median of 30.5 days [236]. These data suggest that herb use was stopped some days before symptoms emerged, but causes of herb discontinuation use were not communicated. This early stop is quite unusual as patients commonly stop herbal use after and not before appearance of symptoms [236].

It was also mentioned that liver injury due to herbal TCM develops slowly between one week and one month [181]. Indeed, patients of the non HSOS group with liver injury by *Polygonum multiflorum* showed a median time of symptom onset of 27 days with a range of 1–120 days in one study [162] or of 30 days with a range of 1–240 days [163], while latency periods were not assessed in another study which reported instead a mean duration of intake of 169 days with a range from 15 to 730 days [164]. A short time to onset of one day or few days puts causality in question, as it seems that the patient started the treatment at a time when a liver disease unrelated to any herb or drug emerged, or even worse, the treatment was considered for symptoms that were liver related but not recognized as such. Within the non HSOS group, patients with liver injury caused by the TCM Lu Cha (green tea), who had used this herb as extracts from two days up to more than one year and showed a time to onset in a range from 14 days to more than one year [141]. In another study of Lu Cha, the liver injury occurred between four days and four years; however, the time to onset was ≤4 weeks in 25% and ≤3 months in 70% [138].

### 7.2. Symptoms and Clinical Features

Whereas fluid accumulation including ascites is common in HSOS due to TCM herbs containing PAs [122], these clinical signs are not observed in patients with liver injury caused by TCM herbs lacking PAs. Instead, these patients experienced a variety of symptoms such as anorexia (58.0%), fatigue (67.3%), jaundice (60.3%), nausea (35.9%), and fever (35.9%), but signs such as rash, pruritus, and pale colored stools have also been reported [181], in support of many case reports and case series [70,71,72,73,74,75,76,77,78,79,80,81,82,83,84,85,86,87,88,89,90,91,92,93,94,95,96,97,98,99,100,101,102,103,104,105,106,107,108,109,110,111,112,113,114,115,116,117,118,119,120,121,122,123,124,125,126,127,128,129,130,131,132,133,134,135,136,137,138,139,140,141,142,143,144,145,146,147,148,149,150,151,152,153,154,155,156,157,158,159,160,161,162,163,164,165,166,167,168,169,170,171,172,173,174,175,176,177,178,179,181,182,184,185,186,187,188,189,190]. Similar symptoms were described in patients with liver injury due to non TCM herbs [50,60,89,210,211,212,213,214,215,216]. In more detail, liver injury by La Chu (green tea) extracts is characterized in 19 patients by the following symptoms: Jaundice (10 cases; 52.6%), nausea (6; 31.5), fever (6; 31.5%), fatigue (5; 26.3%), discoloration of stool and/or urine (4; 21.1%), vomiting (4; 21.1%), malaise (4; 21.1%), pruritus, arthromyalgia, abdominal pain, and epigastric pain, each in two cases (10.5%), as well as low back pain, hypogastric pain, right upper quadrant pain, diarrhea, asthenia, and weight loss, each in one case (5.3%), whereby each patient had at least one symptom and most multiple symptoms [141]. However, no information is provided for these 19 patients as to the sequence in which the symptoms emerged [141], as opposed for instance in a patient with liver injury due to Indian Ayurvedic herbs, who provided the following symptoms in sequence: starting with pruritus, followed by loss of appetite, fatigue, nausea, vomiting, dark urine, light stool, and finally jaundice [50,89]. With *Polygonum multiflorum* as another example of liver injury not related to PAs, most of the patients were admitted for jaundice, fatigue, anorexia, and discolored urine, shown in a report of 18 patients [162], with similar results provided in another publication [163]. Jaundice was the leading symptom in 20/25 patients (80%) with liver injury due to *Polygonum multiflorum*, rarely associated with extrahepatic manifestations such as arthralgia, fever, thrombocytopenia, pancytopenia, eosinophilia, and skin rash [153]. It appears from these studies that patients with liver injury caused by green tea extracts or *Polygonum multiflorum* were rarely monosymptomatic but mostly polysymptomatic. Only few patients with liver injury were described as being asymptomatic [153].

Patients with HSOS present typical symptoms of abdominal distension and pain, ascites, malaise, jaundice, hepatomegaly, and body weight increase due to ascites and edema caused by fluid accumulation [122]. Use of TCM herbs containing PAs may result in splenic measurement at the upper limit of normal as revealed by ultrasonography, but splenomegaly was not observed likely due to short term use of the herbs [125]. Diagnosis of HSOS was established using the modified Seattle criteria [122,237], and causality was proven by RUCAM. Most importantly, the leading symptom of HSOS caused by *Gynura segetum* symptom is ascites, which is otherwise rarely found in patients with liver injury by TCM herbs lacking PAs, except perhaps in end stage conditions of liver cirrhosis. Therefore, ascites must alert the physician considering HSOS as a diagnostic option (Table 6). Ignoring this key findings led to erroneous diagnoses and further harm to the patients, as detailed and discussed in many reports [119,120,121,122,123,124,125,126].

### 7.3. Laboratory Results

Not all patients with liver injury by TCM herbs show LT abnormalitiess. For instance, a few patients with HSOS had normal LT (Table 6) [120,122], low AST values of 54 U/L [129] and 79 U/L [120], or low ALT values of 42 U/L [120]. In a larger group of patients with HSOS, ALT was 243 ± 60 U/L and AST 259 ± 63 U/L [236]. These conditions are challenging and require clinical experience to make an early diagnosis. Such problems are not known for the non HSOS cases of liver injury from most other TCM herbs, because often high LT values are more frequent. For instance, increase in ALT between 195 and 3851 U/L may be caused by green tea extracts [141] or between 601 and 3120 U/L by *Polygonum multiflorum* [164] and within a similar range in another cohort [153].

Of particular interest is an analysis that describes significantly higher mean ALT values for liver injury caused by a single TCM herb as compared to mixtures of multiple herbs (1082.9 ± 503.1 vs. 643.3 ± 530.4 U/L; *p* < 0.05) [238].

### 7.4. Severity of Liver Disease and Hy’s Law

#### 7.4.1. Liver Adaptation

Under a treatment with TCM herbs, mild LT increases such as ALT < 5 *N* may be observed in 0.3% of the patients, as shown in a large cohort of 994 patients (Table 2) [13]. These mild elevations can be either new or pre-existing, for instance caused by a non-alcoholic or alcoholic fatty liver disease. If the ALT elevations occurred newly and are not accompanied by increased bilirubin values or jaundice, they are of no clinical relevance and should be interpreted as liver adaptation to the biotransformation response of the phytochemicals. However, patients with liver adaptation progress rarely to severe liver injury with ALT ≥ 5 *N*, increases of bilirubin or jaundice, requiring LTs monitoring during the treatment. To be cautious, cessation of herbal TCM use should be mandatory for the patient safety, especially if the indication is unclear and treatment was ineffective.

#### 7.4.2. Severe Liver Injury and Acute Liver Failure

The underlying risk factors leading to ALF are unknown [80]. Jaundice is one of the cardinal symptoms in liver injury caused by herbal TCM [153], raising the question as to whether high bilirubin values are risk factors of poor outcome [238] as in DILI cases [198,239]. ALF is more prevalent in patients with HSOS caused by TCM herbs which contain PAs (Table 6) [122], but it can also occur in connection with the use of TCM herbs not containing PAs (Table 7), as shown also in 87 Korean patients with three fatalities and one liver transplantation [238].

In clinical practice and for risk management of these conditions, helpful recommendations derived from Hy’s law should be followed. Indeed, the late Hyman Zimmerman stated that mortality risk of DILI would be around 10% if jaundice appears in the course of hepatocellular injury. This statement has been translated by the US FDA into the following three criteria: (1) serum ALT or AST > 3 *N*; (2) serum total bilirubin elevated to >2 N; and (3) no other reason can be found for the combination of increased aminotransferases and bilirubin [239]. Patients with signs of ALF (e.g., coagulopathy and encephalopathy) should be transferred to a specialized hepatology unit. Up to now, prognostic markers to predict the outcome and to indicate that a patient may require liver transplantation are not evaluated in liver injury caused by TCM herbs.

#### 7.4.3. Natural Course and Outcome

Following cessation of the herbal use, most patients with liver injury caused by herbal TCM recovered quickly, more so when the causative herbs were free of PAs [141] as compared to herbs containing PAs (Table 6) [122]. However, few patients continue the consumption of TCM herbs despite symptoms, but it is not clear whether this is risky for these patients [141]. As expected, there are no prospective studies that would evaluate whether continued use of TCM herbs despite symptoms modifies the outcome. Continued use despite symptoms was a significant problem in patients using isoniazid and experiencing liver injury, as the delayed cessation of the antituberculous drug was associated with a high risk of fatality. Among 13 patients with continued INH use for more than seven days, seven patients required a liver transplantation or died [50,240]. Conversely, continued use of Greater Celandine in patients with symptomatic liver injury was not associated with an increased risk. Indeed, five patients with overt symptoms, who continued treatment over a period of up to seven months, had a favorable outcome that was contrary to clinical expectations [50,215]. Despite these differences of outcome depending on the causative product, intake of the herbal TCM products must be stopped when symptoms of liver injury occur, considered this as a precautionary measure [50].

## 8. Biomarkers: Microsomal Epoxide Hydrolase, Pyrrole-Protein Adducts, HLA, MicroRNA, and Metabolomics

Herbal TCM may cause a large spectrum of liver injury, affecting all cells present in the liver and biliary tree, and ranging from mild asymptomatic liver test (LT) elevation to acute or chronic liver injury, cirrhosis, ALF, acute and chronic cholangitis, macro- and micro-vesicular steatosis, and vascular lesions, as provided in many reports [70,71,72,73,74,75,76,77,78,79,80,81,82,83,84,85,86,87,88,89,90,91,92,93,94,95,96,97,98,99,100,101,102,103,104,105,106,107,108,109,110,111,112,113,114,115,116,117,118,119,120,121,122,123,124,125,126,127,128,129,130,131,132,133,134,135,136,137,138,139,140,141,142,143,144,145,146,147,148,149,150,151,152,153,154,155,156,157,158,159,160,161,162,163,164,165,166,167,168,169,170,171,172,173,174,175,176,177,178,179]. However, reviewing these publications, the clinical diagnosis of liver injury from herbal TCM in these cases remains often unclear. In fact, all authors claimed having validly established the diagnosis and verified the causality, although in some cases only a possible causality for the used herbal TCM product was suggested or data quality was marginal at best. In their recent article on drug-induced liver injury and the highlights from a review of the 2015 literature, Sarges et al. [192] stated that in suspected cases of liver injury, the ability to confidently assign causality to a particular drug, chemical, or herbal agent alone has become increasingly complex. They recommend an exhaustive causality assessment and propose a model on which cases of possible liver injury by drugs and herbs should be based [192], referring to published work [241,242].

### 8.1. Diagnostic Biomarkers

There are very few instances in which liver injury from herbal TCM is associated with specific, mechanism-based biomarkers, which can be measured in the blood. Such mechanistic biomarkers are presently well documented for three TCM herbs which cause intrinsic liver injury, namely the TCM herb Shi Can (*Teucrium chamaedrys*, Germander) [168,169,170,171,172,173,174,175,176,199,200,201], the TCM herb San Chi (*Gynura segetum*) [120,121,122,123,124], and the TCM *Polygonum multiflorum* [164]. Diagnostic biomarkers follow specific analytical methods and can be determined in fluids such as the blood of the patients, who experience liver injury by TCM herbs.

For Germander, specific autoantibodies are the diagnostic tools [201], and for PA containing herbs, protein adducts and conjugates are of diagnostic value [120,122,123,129,201,236,243]. Conversely, for *Polygonum multiflorum*, chemical ingredients of the plant and their metabolites provide diagnostic information, whereby only the metabolites are classified as mechanistic biomarkers [164].

#### 8.1.1. Established Diagnostic Biomarkers

##### 8.1.1.1. Microsomal Epoxide Hydrolase

With anti-microsomal epoxide hydrolase autoantibodies, a most interesting and one of the first examples of a diagnostic, mechanism-based biomarker of liver injury induced by a TCM herb was established [201]. Primarily located at the surface of the hepatocytes, these autoantibodies were found in the sera of patients who experienced liver injury after consumption of teas prepared from the TCM Shi Can (Germander) for a long period of time [201]. These autoantibodies are the diagnostic biomarkers, easily assessable in the serum of patients with such liver injury. The specificity of these biomarkers allows attributing cases of liver injury to the ingestion of Germander, which has been responsible for the liver injury of numerous patients [168,169,170,171,172,173,174,175,176,199,200,201]. As Germander hepatotoxicity is reproducible in mice, the various steps leading to these antibodies have successfully been explored [199,200,201]. It was early recognized that the chemical composition of Germander comprises furan-containing neoclerodane diterpenoids. These Germander ingredients are oxidized by CYP3A to reactive metabolites in the microsomal fraction of the hepatocytes, which corresponds to the endoplasmatic reticulum upon electron microscopy assessment. Reactive metabolites likely interact with the microsomal epoxide hydrolase and trigger the formation of the respective autoantibodies, which escape from the liver into the blood. These antibodies recognize teucrin A-alkylated epoxide hydrolase.

##### 8.1.1.2. Pyrrole-Protein Adducts

The second example of a diagnostic, mechanism-based biomarker of intrinsic liver injury caused by TCM herbs relates to PA poisoning, for which a new analytical method was established [120,122,123,129,201,236]. Using ultra performance liquid chromatography-mass spectrometry (UPLC-MS) analysis, quantitative determination of pyrrole-protein adducts (PPAs) in the blood of affected patients became feasible. This represents a diagnostic breakthrough and is encouraging, as it facilitates the clinical assessment of patients with liver injury, who may or may not have used TCM herbs containing PAs. Blood PPA concentrations were positive in all 23 patients with HSOS caused by PA-containing TCM herbs [236]. The sensitivity of the test was 100% and the specificity was 95.8%, while the negative predictive value was 100% and the positive likelihood ratio was 23.81. The blood PPA levels tend to decrease quickly within 40 days and then decreased more slowly until it was undetectable within 300 days, indicating that PA metabolites can remain in the blood for a relatively long period of time [236].

Liver injury by PAs is a major health issue as exemplified by two TCM herbs, San Chi (*Gynura segetum*), which contains PAs, and Jing Tian San (*Sedum aizoon*) lacking PAs, whereby San Chi was misused in medicinal preparations instead of the nontoxic Jing Tian San [119,120,121,122,123,124,125,126,127,128,129,201,229,230,231,232,233,234,235,236]. Similar botanical names as well as lack of proper plant authentication and diagnostic tools created this confusion. These problems stimulated investigations on a diagnostic biomarker of liver injury caused by PAs and led to the development of the PAA biomarker, which is mechanism-based on the knowledge of the reactive metabolites PAAs generated from unsaturated PAs [120,122,123,129,201,236,243]. PAAs bind covalently to DNA, albumin and other proteins, forming pyrrole-protein adducts which enter the circulation. It seems that PAAs are generated by microsomal CYPs in the liver parenchymal cells [120,201,229,230], but it remains unclear why toxicity occurs preferentially at another site, in the hepatic sinusoidal endothelial cells rather than in the hepatocytes [243,244].

Unclear is also whether the low glutathione (GSH) content in the sinusoidal endothelial cells may render these cells more susceptible to injury [243]. The functions of hepatic sinusoidal endothelial cells have well been described [245,246,247,248], but further studies in laboratory animals may be needed in order to clarify how PAs or PAA intermediates are transferred from the hepatocytes to the sinusoidal endothelial cells. In animals treated with PAs, electron microscopy studies showed disruption of the hepatocyte plasma membrane, associated with an extensive bleb formation at the sinusoidal membrane of the hepatocytes [246]. This occurs nearby the sinusoidal endothelial cells with their well strong endocytic activity [247]. There is also evidence that PAs undergo CYP-dependent metabolic activation in the liver sinusoidal endothelial cells [248], suggesting that the intermediates may be generated in these cells. Whether all these processes occur simultaneously or one by one remains to be established.

##### 8.1.1.3. Metabolomics

For liver injury induced by *Polygonum multiflorum*, other diagnostic approaches were used, classified as metabolomic biomarkers [164]. These analyze in the blood of patients with liver injury likely caused by *Polygonum multiflorum* for the chemicals lysophosphatidylcholines, phosphatidylcholines, prostagladins, fatty acids, 2,3,5,4′-tetrahydroxy *trans*-stilbene-2-*O*-β-d-glucoside (TSG), and many others. This clarifies whether a patient used this herb or a fraudulent substitute. Analyses were done with a liquid chromatograph coupled with a high-resolution hybrid quadrupole time-of-flight mass spectrometer (LC-QToF-MS) [164].

### 8.2. Progress in Developing Valid Diagnostic Biomarkers

The above discussed diagnostic biomarkers for the three TCM herbs are encouraging and may certainly be extended to additional TCM herbs that are responsible for intrinsic liver injury. Unquestionably, most promising are mechanistic biomarkers in order to establish the diagnosis of liver injury related to TCM herbs, a view supported also with respect to DILI by another report [249]. However, approaches may not be extended to idiosyncratic liver injury since its causative intermediates are generated in small amounts in the liver cells and not prone to substantial leakage into the circulation. In addition to the diagnostic biomarkers, there is much interest in other biomarkers not necessarily aiming to confirm the diagnosis of advanced injury but with focus on improving early recognition of liver injury [192,250,251,252,253,254], or perhaps assessing individual susceptibility [192,255,256]. Many studies of these new biomarkers for DILI were published and discussed, but the question was raised as to whether they really are better and what do they diagnose [249]. Critical evaluations of these studies are needed [192,249], also in view of the flood of recent publications, shown by more than 620,000 PubMed quotations and over 40,000 more per year in the last two years [249].

#### 8.2.1. Human Leucocyte Antigen (HLA)

Many studies relating to liver injury elucidate the role human leucocyte antigen (HLA) genotype testing for predicting liver injury in susceptible individuals. However, it remains to be established whether this testing is applicable to liver injury due to herbal TCM. The actual importance of such testing in the general population remains unclear due to currently low positive and negative predictive values of the tests [192]. With respect to DILI, characterization of genetic susceptibility by demonstration of a strong association between a specific HLA haplotype and the liver injury has been recently made with many drugs, including lumiracoxib, abacavir, lapatinib, antitubercular drugs, and flucloxacillin [192,249,255].

For instance, an association was found and described in detail for lumiracoxib and HLA DQA1*0102 [249] as well as for flucoxacillin and HLA-B*5701 [255]. Whether HLA genotypes can be used in the future as diagnostic biomarkers, as a parameter for risk factors, or as pathogenetic hallmarks requires further studies.

#### 8.2.2. MicroRNA

Microarray RNA (microRNA) are short (18–25 nucleotides) noncoding RNA molecules and function to repress specific target messenger RNAs and thereby regulate specific cellular proteins and the phenotype of the cell [257]. There is substantial interest in the potential role of these microRNAs for the early recognition of liver injury caused by drugs and TCM herbs in place of aminotransferases which are considered by some as insufficiently sensitive in clinical trials of drug development and insufficiently organ specific [192,249,250,251,252,253,254,257,258,259,260]. MicroRNA can be measured in the blood of patients with liver injury caused by drugs such as acetaminophen (AAP) and may have a positive predictive value in determining the outcome of AAP hepatotoxicity [192]. Such biomarkers can also be used in determining the need for liver transplantation in AAP intoxication and were found to have a higher sensitivity and specificity in patients with liver injury, who used AAP and received drug treatment for HIV and tuberculosis [192,259]. Despite encouraging progress of micoRNA evaluation in liver injury due to drugs, final recommendations for pre-clinical trials and clinical use were considered premature [192,249]. Specific aspects of circulating microRNA molecules as biomarkers in liver disease were also critically addressed in a recent thorough clinical review [253] and analyses of experimental liver injury due to TCM Chuan Lian Zi (*Melia Toosendan*) [252] or the TCM Huan Yao Zi (*Dioscorea bulbifera*) and AAP [260].

Similar restrictions as those for drugs may apply to herb induced liver injury caused by TCM [192,252,260]. Under these conditions, some details on this subject warrant further considerations. Basically, microRNA circulating in the blood may be found with low levels in healthy individuals and with higher levels in patients with various diseases such as cancer, cell and organ transplantation, coronary heart disease, stroke, sepsis, burns, or confined to specific organs [253]. Blood microRNAs can be differentiated regarding their organ specificity, thereby allowing for instance quantitative analysis for liver specific subtypes of microRNAs [192,249,253,257]. They originate from the injured liver, whereby multiple mechanisms are involved in the release of microRNA from the hepatocyte such as passive processes; e.g, simple leakage [253]. Following cell death, microRNA can reach the extracellular environment bound and protected by subcellular structures. Among active processes, microRNA release via apoptotic bodies and microvesicles are known. During apoptosis, cellular microRNA is packed into granules and subsequently into apoptotic bodies [253]. Presently, most microRNA studies dealing with liver injury focused on drugs in both patients and animals, whereas only few reports evaluated liver injury by TCM herbs, as assessed in animals but not in patients.

For experimental liver injury from herbal TCM, microRNAs were studied in mice using the TCM Chuan Lian Zi (*Melia Toosendan*) [252]. This TCM herb is a known hepatotoxic in humans but poorly described in the English scientific literature [82,181,252] and should be classified as a herb causing intrinsic liver injury due to its reproducibility in animals [252]. These animal studies showed that the TCM Chuan Lian Zi caused changes of eight microRNAs and 1723 messenger RNAs. For the eight differentially expressed microRNAs, their predictive target genes were collected, and further analyses revealed that several cellular functions were affected, such as cellular growth and proliferation, gene expression, and cellular development.

All these studies show that the use of microRNA may uncover toxicological mechanisms leading to liver injury by this TCM herb. It remains to be established whether similar findings of microRNA are found in the blood of patients with liver injury caused by this type of herbal TCM.

In the other liver injury study that used both the TCM Huan Yao Zi (*Dioscorea bulbifera*) and AAP for comparison, the microRNA profiling was different [260]. It was not studied whether this difference applies also to other drugs or herbs. If it is confirmed, microRNA measurements could be used in patients with liver injury to analyze the cause, synthetic drug versus herb.

Although some work including careful standardization of microRNA assays is still necessary, serum microRNAs are promising biomarkers for assessing cases of liver injury, bearing in mind the following points: (1) microRNAs could be a more sensitive and specific diagnostic marker of incipient liver injury assessable prior to conventional markers such as transaminases; (2) its determination could especially be useful in future trials with drugs and herbs including TCM herbs but studies are still warranted to assess both idiosyncratic and intrinsic liver injury; (3) if confirmed, microRNAs’ assessment in clinical trials could assist or replace the present use of eDISH (evaluation of Drug Induced Serious Hepatotoxicity), a software program which facilitates recognition of a liver safety signal in clinical trials [261,262,263,264]; (4) microRNAs fingerprinting might be able to identify in a patient with liver injury whether a specific drug or herb was responsible; and finally (5) microRNA subtypes could elucidate possible mechanism leading to liver injury. Future studies will have to clarify these microRNA issues, as drawing from published data any definite conclusion on the clinical use of microRNAs would be premature.

## 9. Diagnostic Challenges

### 9.1. Minimum Quality Requirements of Herbal TCM Products

Herbal products are manufactured for human use and require quality and safety standards; lacking these is a major issue and often communicated in several critical reports within the last years [3,28,29,50,60,122,209,265,266,267,268,269,270,271,272]. Similar problems are evident for most herbs used in the context of TCM [8,12,15,17,62,63,64,65,66,67,119,120,121,122,123,124,181,185,229,230,231,232,233], which were discussed by the Chinese FDA, presenting details of the current status and future perspectives of the pharmacovigilance practice and risk control of TCM drugs in China [12].

### 9.2. Variability of Herbal TCM Product Names, Quality, Misidentifications, Adulterants, and Impurities

The Chinese FDA provides transparency on the risks and issues of the herbal TCM, considering many details were poorly discussed in the past [12]. The vast and diverse land area of China is a large reservoir of TCM herbs, which contributes to the complexity of the herb species, their origins and names, since often the same name is used for different TCM herbs or different names for the same herb. In addition, similar names or abbreviations for different species lead to confusion. This problem is also documented in case narratives presented above.

Problems with the nomenclature were summarized in another report [52]. TCM clinical practice also considers the place of origin of the TCM herbs as an important factor; some places are thought to be superior but qualifying criteria were not provided [12].

It is recognized that differences in the growing conditions and geographical origin may influence the chemical profiles of the TCM herbs, which may influence therapeutic efficacy and toxicity [12]. Herbal TCM product quality may be influenced by adulterations with synthetic drugs and pollution due to contamination with pesticides, heavy metals, or radioactivity, inadequate processing procedures, storage, solvents, solubilizers, and incorrect preparation of the TCM decoctions that relies on “the rules of the thumb” as advised by the prescribers [12].

It is unclear, however, to what extent these quality deficits contribute to liver injury [50]. It seems that most TCM herbal products meet quality standards. Notably, in one study in Germany, analyses of herbal TCM products showed no quality problems [13] and in a French report of liver injury induced by Shi Can (germander), analyses showed lack of product contamination by insecticides or microorganisms [273].

Several other reports focused specifically on topics such as safety [3,7,8,11,13,14,15,25,29,59,67,164], herbal TCM product quality [7,8,13,17,25,26,50,60,164]. TCM herb authentication by microscopy and especially DNA barcoding [164], microbial or mycotoxin contamination [13,65,164], herbal misidentification [50,60,122,123,124,164], adulterants [50,60,62,63], heavy metals [63,64,65,164], pesticides [164], and other impurities [50,60]. It must be guaranteed that all TCM herbs meet product quality standards, considering the rules of current Good Agricultural Practices (cGAPs) and current Good Manufacturing Practices (cGMPs) (Table 8).

### 9.3. Case Data Quality

Reviewing the case reports and case series of suspected liver injury induced by TCM herbs, it is clear that the data quality of the cases is often poor [70,71,72,73,74,75,76,77,78,79,80,81,82,83,84,85,86,87,88,89,90,91,92,93,94,95,96,97,98,99,100,101,102,103,104,105,106,107,108,109,110,111,112,113,114,115,116,117,118,119,120,121,122,123,124,125,126,127,128,129,130,131,132,133,134,135,136,137,138,139,140,141,142,143,144,145,146,147,148,149,150,151,152,153,154,155,156,157,158,159,160,161,162,163,164,165,166,167,168,169,170,171,172,173,174,175,176,177,178,179,181,182,184,185,186,187,188,189,190]. There are, however, suggestions on how data should be captured, presentation including narratives, and evaluation of causality assessment can be improved [274] and which approaches should be undertaken [192]. Most promising is the prospective data acquisition at a time when liver injury is suspected, using the items of the RUCAM and a checklist of differential diagnoses that should be excluded (Table 9) [51].

This tabular listing is derived from a previous publication [51]. Although not comprehensive, it is to be used as a guide and in connection with RUCAM [51]. AAA, Anti-actin antibodies; AMA, Antimitochondrial antibodies; ANA, Antinuclear antibodies; ASGPR, Asialo-glycoprotein-receptor; BMI, Body mass index; CT, Computed tomography; CYP, Cytochrome P450; DPH, Pyruvate dehydrogenase; HAV, Hepatitis A virus; HBc, Hepatitis B core; HBV, Hepatitis B virus; HCV, Hepatitis C virus; HEV, Hepatitis E virus; HILI, Herb induced liver injury; HIV; human immunodeficiency virus; LKM, Liver kidney microsomes; LP, Liver-pancreas antigen; LSP, Liver specific protein; MRC, Magnetic resonance cholangiography; MRT, Magnetic resonance tomography; p-ANCA, Perinuclear antineutrophil cytoplasmatic antibodies; PCR, Polymerase chain reaction; RUCAM, Roussel Uclaf Causality Assessment Method; SLA, Soluble liver antigen; SMA, Smooth muscle antibodies; TSH, Thyroid stimulating hormone; TTG, Tissue transglutaminase.

## 10. Hepatotoxicity Criteria and Liver Injury Pattern

**Figure 4:**
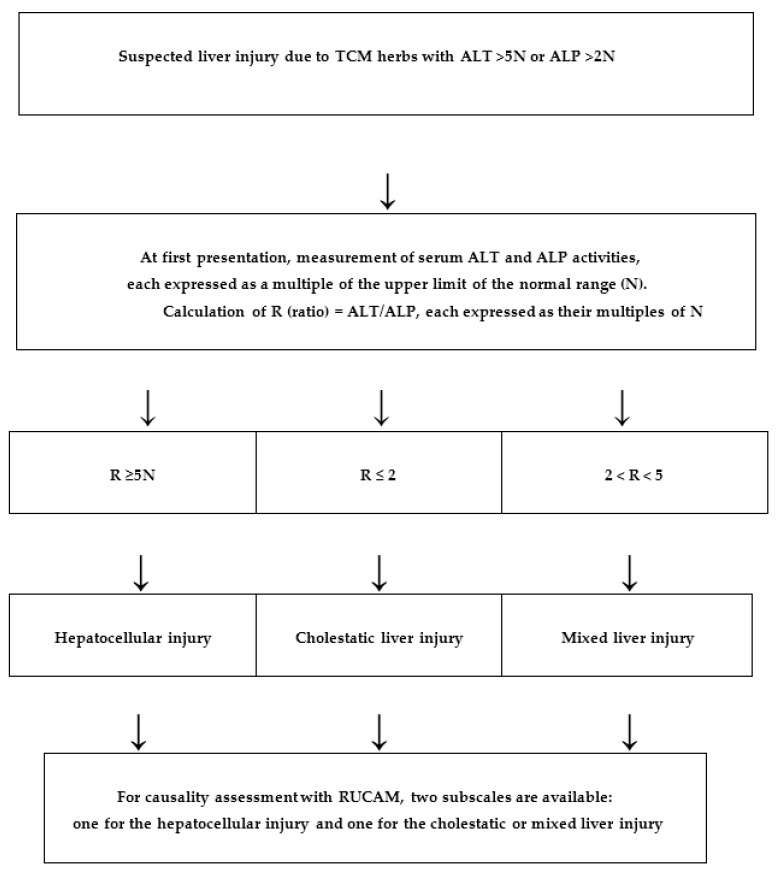
Classification of liver injury and its pattern. Adapted from a previous report, which provides additional information [51]. ALP: from hepatic origin. ALP, alkaline phosphatase; ALT, Alanine aminotransferase; N, Upper limit of normal; R, Ratio; RUCAM, Roussel Uclaf Causality Assessment Method.

Criteria of liver injury and liver injury pattern are essential for case assessment of suspected liver injury caused by TCM herbs (Figure 4).

The overall accepted definitions of liver injury and the liver injury pattern (Figure 4) goes back to when RUCAM was developed in 1993 [275,276] and has remained unchanged except for one criterion which was changed to ALT > 5 *N* [51]. These definitions can be applied to suspected liver injury caused not only by TCM herbs, but also by other herbs and drugs (Figure 4). Hepatocellular injury prevails in most TCM related case series [164,181,238].

## 11. RUCAM as the Preferred Method to Assess Causality in Liver Injury Caused by TCM Herbs

Causality for TCM herbs in patients with suspected liver injury is best assessed with RUCAM in the version updated in 2016 [15] and based on the original version [275,276]. Assessment requires to determine the type of injury: hepatocellular type or cholestatic or mixed type (Figure 3) [51]. RUCAM represents a structured, standardized, validated, and hepatotoxicity specific diagnostic approach, which attributes scores to key items that reflect the natural course of the liver injury, ALT dechallenge features, and search for alternative causes [51].

RUCAM pays special attention to all core elements that are relevant to liver injury caused by TCM herbs, as described in detail [15]. In short, the key items are assessed in seven domains: (1) the time to onset from the beginning of the herb intake, with specific criteria that are well scored and clearly defined with a time frame between beginning of the herb use with day 0 as the first day of intake and the onset of increased LTs or symptoms; (2) precise dechallenge criteria with scores reflecting the natural course of LTs after cessation of the suspected herb are cornerstones of RUCAM, while treatment during the dechallenge phase with drugs such as steroids or ursodesoxycholic acid may mask the natural course and allows only a score of 0, reflecting no information; (3) risk factors such as current alcohol use and age are to be considered; (4) concomitant use of drugs and herbs is a crucial item, requiring details of a temporal association and potential hepatotoxic features of the used product and a separate evaluation; (5) the search for alternative causes considers the clinically most relevant causes such as infections by HAV, HBV, HCV, HEV, CMV, EBV, HSV, and VZV, to be assessed by parameters such as PCR and IgM/IgG antibody titers also upon repetitive analyses, as well as complications of underlying disease(s); (6) previous hepatotoxicity listed in the product information sheet (e.g., Summary of product characteristics in the EU or product information in the US) must be checked; and finally (7) the response to any unintentional reexposure has to be evaluated, as to whether specific criteria of a positive test result are fulfilled. Items of these seven domains provide individual scores for each suspected TCM herb in a case report, giving a final quantitative grading of causality: ≤0, excluded; 1–2, unlikely; 3–5, possible; 6–8, probable; ≥9, highly probable [51]. However, RUCAM based assessment should not be limited to providing the final score. Instead, for reasons of transparency and reassessment, each item with its score for any TCM herb and concomitant product must be presented [274], not only to regulatory agencies and the manufacturer but also and more importantly to editors if a publication as a case report or case series is considered. Lacking presentation of individual RUCAM based data would invalidate such publications.

This scoring system of RUCAM is unique and meets the requirements of clinicians and practitioners in care for their patients with suspected liver injury caused by TCM herbs, assisting to establish the timely diagnosis with a high degree of certainty. RUCAM is user-friendly as there is no need of discussions among experts, who are not always available when their opinion is requested.

RUCAM is the most commonly used causality assessment method for injury cases worldwide and is also intensively used in the Asian region [51] including China where it is applied and recommended [51,277]. RUCAM has many advantages compared to other methods attempting to assess such causality (Table 10) [51].

In many countries and for more than two decades, physicians, experts in clinical hepatotoxicity, international registries, regulatory agencies, published case reports, and pharmaceutical companies successfully applied RUCAM for suspected liver injury, with increasing tendency [51]. RUCAM was also often applied in cases of liver injury caused by TCM herbs (Table 11) [80,82,104,105,110,111,112,113,120,122,134,138,151,162,164,165,186,221,278,279,280].

RUCAM is rarely used in the United States (Table 11 and Table 12), where the DILIN method is preferred, which circumvents scoring of individual items and obviates transparency (Table 10) [51] as shown also in a recent report of seven cases with suspected liver injury [281]. In this study, RUCAM causality levels were low with unlikely in one case, possible in four cases, and probable in two cases only; conversely, the DILIN method attributed a probable or higher causality to six cases and a possible causality to one case. Case evaluations were confounded by an undefined reexposure test, several comedications, as 5/7 patients (71.4%) used up to two other prescription medications or herbal dietary supplements, and preexisting chronic HBV infection, which caused liver cirrhosis, requiring a liver transplant. The suggestion that DILI may have superimposed on chronic HBV infection [281] appears unlikely in view of clinical experience and a previous analysis [198].

Also, it appears that only part of the requested alternative virus infections such as HSV and VZV was excluded, which creates problems as any diagnosis of organ injury is a diagnosis of exclusion, not fulfilled in this report [281]. Even more problematic was the approach to exclude HEV, as assessment was confounded by positive anti-HEV IgG titers, incomplete analysis of essential HEV parameters, and not prospective evaluations. HEV-RNA was not at all determined in any of the cases for unknown reasons, and the used HEV antibody assays were not specified [281], disregarding the well-known problem of lacking FDA approval [241,242,282]. For none of these seven patients, product use or purchase was confirmed, nor was a product analysis done. Suggestions how to provide good evaluations of case data were made recently by scientists from the USA [192]. Their recommendations should be followed in the future.

Several international registries and regulatory agencies applied RUCAM for causality assessment in liver injury caused by TCM herbs (Table 12) [136,153,283,284,285,286].

## 12. Reexposure

One way to firmly establish the diagnosis of an initially suspected liver injury by TCM herbs is by using positive test results if these are available [51]. Intentional reexposure tests are obsolete due to the high health risk for the patient. Case reports and case series of suspected liver injury caused by various TCM herbs, other herbs and herbal dietary supplements may contain the information that in one or the other patient a positive reexposure test was found. In most reports, however, details of the results were not provided and did not allow a valid conclusion. For instance, among 53 cases with liver injury, a positive reexposure test was reported in eight patients, but the reanalysis of these published reexposure tests with claimed positive results was disappointing, since in only one case out of these eight cases a positive test result could be verified; in another case, the test result was clearly negative, while in the remaining six cases, data were considered as uninterpretable due to missing information to comply adequately with specific criteria [287]. In these eight cases, causality reassessment with RUCAM revealed causality levels of probable in one case and of unlikely and excluded in four and three cases, respectively [287]. Therefore, both RUCAM and unintentional reexposure tests are valuable tools to assess causality [287], while similar and confirmatory results were subsequently published with RUCAM [288].

Similarly, in 25 cases of suspected liver injury caused by TCM herbs, the authors claimed a positive reexposure test result; however, this result was confirmed upon reassessment in only 14 cases using a criteria based approach, while the reevaluation in the remaining nine patients provided either negative or uninterpretable test results [180]. For future cases of reported positive reexposure tests, special attention should be paid, as to whether specific criteria were such as those published recently in connection with details of RUCAM [51]. To circumvent inappropriate claims that a positive reexposure test could have established causality in liver injury cases, it is of importance to present details of such reexposure. For a positive reexposure test result, several criteria must be fulfilled (Table 13) [51].

## 13. TCM Herbs with Established, Questionable, or Lacking Liver Injury and Listing Compilation

Many publications reporting cases of liver injury caused by TCM herbs used RUCAM to assess causality (Table 11 and Table 12) and provided RUCAM based causality levels, which varied from highly probable to unlikely or excluded [80,82,104,105,110,111,112,113,120,122,134,136,138,151,153,162,164,165,186,221,278,279,280,283,284,285,286]. Often, only final RUCAM scores were presented and no case details that precluded subsequent reassessment. Nevertheless, these publications are important as they may give some impression of the tentative number of patients involved and the causality grading. The difficulties to arrive at a valid causality determination in liver injury cases may be illustrated with the example of two TCM herbs, *Polygonum multiflorum* and Lu Cha (green tea).

With respect to *Polygonum multiflorum*, substantial progress has been made to clarify to what extent liver injury is caused by this TCM herb. This topic was mostly approached in case series of suspected liver injury from Polygonum multiflorum, but specific causality assessment was either lacking or fragmentary [150,153,161,162,163,164]. The first review of a case series goes back to 2009, when Zhang et al. [161] reported in a Chinese medical journal on 24 liver injury cases from *Polygonum multiflorum*, which were retrieved from the scientific literature published between 1978 and 2008 but were not submitted to a regular causality assessment method. In 2015 and again without any use of a causality assessment method, data on 450 patients with suspected liver injury caused by *Polygonum multiflorum* were reported by Lei et al. [163]. In these two studies [161,163], clinical features of suspected liver injury from *Polygonum multiflorum* were carefully analyzed and presented, but in face of a lacking formal causality assessment the obtained data must be considered as vague, unless causality will now be done as expected from the scientific community.

The breakthrough came in 2011, when the RUCAM based report by Jung et al. [153] expanded our knowledge on this topic and provided details of 25 cases with a probable causality in 15 patients and a highly probable causality in 10 patients. RUCAM was also used in two other studies that were published in 2014 [162,164]. Dong et al. [162] reported the results of 18 patients with liver injury following ingestion of *Polygonum multiflorum*. Upon RUCAM based causality assessment, 14/18 patients (77.8%) reached a highly probable causality, with four patients (22.2%) who achieved a probable causality level. Such high causality levels are likely due to the strict inclusion criteria which led to exclusion of all patients who were comedicated with any other herb or drug. Primarily excluded were also patients with any virus infection.

In addition, Wang et al. [67] reassessed published cases of liver injury from *Polygonum multiflorum* and found in only 45/147 cases (30.6%) a highly probable causality by RUCAM criteria, whereas the assessment of 40 own hospitalized cases revealed a figure of 22.5% (9/40 cases). For the hospitalized group, proposals involving pharmacognosy, phytochemistry, and metabolomics tests were made to improve causality levels, but these techniques are not routinely available at other centers. Zhu et al. [187] also used RUCAM in another study of liver injury caused by *Polygonum multiflorum* and found that of 158 liver injury patients, who used *Polygonum multiflorum* preparations, 92 (58.2%) combined with Western medicine or Chinese herbal preparations without *Polygonum multiflorum*.

Lu Cha (*Camellia sinensis,* green tea) or more specifically green tea extracts (GTE) and their potential hepatotoxicity was for the US Pharmacopeia (USP) an early regulatory matter [137], which would have been best solved with the use of a liver specific causality assessment method such as RUCAM. Instead and in line with the disputed approach for another herb namely black cohosh [265,289,290], USP again used for the suspected cases of liver injury from GTE the Naranjo method [137] with its known problematic lack of liver specific characteristics [265,290,291], as opposed to the liver specific RUCAM (Table 10). The USP approach led to uniformly possible causality levels for all evaluated cases [137]. Such uniformity is unusual and signifies a low discriminating power of the used method. Indeed, the Naranjo scale was considered by Liss and Lewis [291] as too sensitive, allowing a possible causality even in the absence of essential data or by virtue of the patient simply having taken the suspected agent, and concern has been expressed that a more sophisticated judgment would have been expected from the USP. In a clinical context, such low causality levels are usually of little relevance.

Assessing the GTE cases, USP correctly acknowledged the limitations of dietary supplement adverse event reports, most of which contain incomplete information or confounding variables [137]. However, recognizing the need for consistent evaluation using reliable causality scales, USP adopted as a means of achieving consistency in evaluations and minimizing biases through the use of a validated causality scale. Notably, the Naranjo scale has never been validated for liver injury cases, which should have excluded the use of the Naranjo scale for the USP assessment. Another statement of the USP is not founded, that the facility of the Naranjo scale to accommodate the limitations of missing data was considered a further bonus [137]. The limitations of the Naranjo scale were summarized recently, which was designed for evaluation of toxic drug reactions and contains questions regarding drug concentrations and monitoring, dose relationship, including decreasing dose, placebo response, cross-reactivity, and confirmation of the adverse drug reaction using unidentified objective evidence [290].

Attempts of the USP Dietary Supplements Information Expert Committee (DSI EC) are underway to more critically reevaluate the GTE cases. It is hoped that RUCAM will then be used and the reexposure test criteria are carefully applied by the experts in order to overcome prior shortcomings. Subsequent RUCAM based assessments showed that causality could be validly established for only part of the GTE cases [138,140,141].

Of note, a placebo-controlled parallel study to evaluate the chronic effects of GTE given before meals on LTs showed no negative effects on LTs when used for three weeks [220]. This time frame may be too short to allow a final conclusion since GTE was used partially much longer by patients who experienced liver injury [141]. As fasting increases the intestinal absorption of GTE catechins [140], this condition may have prevailed in the clinical cases and could have not been considered appropriately in the GTE study [220].

Going back to the majority of TCM herbs and considering published cases of suspected liver injury that were evaluated for causality by RUCAM, positive reexposure tests meeting specific criteria, or both, it appears that many TCM herbs may be potentially hepatotoxic, sometimes confined to a very small group of cases (Table 14) [50,138,180,188,221,292].

For few TCM herbs, initial claims of their hepatotoxic potency have to be questioned. For instance, it is now clear that the TCM Sedum aizoon lacks hepatotoxicity, as hepatotoxic PAs are not ingredients [120]. Similarly, there is also lack of convincing evidence that the TCM Ba Jiao Lian (*Dysosma pleianthum*) is hepatotoxic, as assumed previously [53,71,72,73] and outlined for this TCM under Narratives above. 

## 14. Chronic Courses and Injury Risk from TCM Herbs for Preexisting Liver Disease

As for DILI, some authors suggested that liver injury from TCM herbs may cause chronic sequalae, but this is not yet sufficiently established, especially in countries with a high prevalence of chronic HBV and HCV infections; these infections must be considered as confounders, unless they were excluded with a high grade of certainty. Conditions are clearly different in cases of HSOS from PAs that damage sinusoidal endothelial cells without chances of complete restoration (Table 6) [122]. 

The relationship between TCM herbs and pre-existing liver disease is complex particularly when increased LTs or new symptoms emerge in patients with pre-existing liver disease during herbal TCM use. This requires two strategies to assess whether these changes are due to liver injury from the TCM herb as a new event or to flares of the underlying liver disease. Lacking a valid diagnostic biomarker, liver injury from TCM herbs is a diagnosis of exclusion and requires causality assessment by RUCAM. Flares of pre-existing liver disease can reliably be assessed in some hepatotropic virus infections by polymerase chain reaction (PCR) and antibody titers at the beginning and in the clinical course to ascertain flares during the natural course of the disease. Unfortunately, flares cannot be verified in many other liver diseases such as alcoholic liver disease, since specific tests are unavailable. However, such a diagnostic approach using RUCAM and biological markers of pre-existing liver diseases and would determine whether TCM herbs or underlying liver diseases caused the LT abnormalities or the new symptoms. More importantly, a clear diagnosis is essential to ensure effective disease management by cessation of the suspected herb. Similar considerations apply to DILI [198].

## 15. Comparison with Drug Induced Liver Injury

A recent study evaluated characteristics of liver injuries from TCM herbs as compared to those from Western drugs in overall 1985 cases following causality assessment by RUCAM [188]. In this analysis, 870 patients (43.8%) used Western medicine, 563 patients (28.4%) took TCM herbs, while 552 patients (27.8%) coadministered Western medicine and TCM herbs. Peak ALT values as expressed as means were similar in the Western medicine group as compared to the TCM herb group and did not allow an ALT based differentiation. Prognosis with good outcome was similar in the two groups, with a marginally higher fatality rate in the TCM herb group and a marginally higher transplantation rate in the Western medicine group. The most commonly implicated TCM herbs were *Polygonum multiflorum*, *Psoralea corylifolia*, *Corydalis yanhusuo*, *Rheum officinale*, *Cassia obtusifolia*, and *Aconitum carmichaeli* [188], all of which had a high casualty level as established by RUCAM and were listed under the TCM herbs with confirmed causality (Table 4). In another article based on data of patients with ALF or acute liver injury in 32 academic centers in the US, the comparison of the severity between DILI and HILI including complementary medicines and dietary supplements showed that the HILI group had a higher rate of transplantation and lower transplant-free survival in those who progressed to ALF [293].

## 16. Outbreaks of Liver Injury from TCM Herbs

Several publications report liver injury outbreaks related to consumption of food contaminated by plants, which contain unsaturated PAs and are also in use as TCM herbs in mainland China [294,295,296,297]. For example, following a two-year period of severe drought, a very large number of patients with massive ascites and emaciation were observed in northwestern Afghanistan [294]. Clinico-pathological studies showed that these were typical cases of HSOS. The outbreak was caused by consumption of bread made from wheat contaminated with seeds of *Heliotropium* plants, which were shown to contain PAs. Examination of 7200 inhabitants from the affected villages showed evidence of liver disease in 22.8%. Clinical improvement was observed in thirteen cases after three to nine months, and in three cases liver biopsies showed almost complete disappearance of initial abnormalities [294]. A more recent outbreak of HSOS was reported from Western Afghanistan, associated with exposure to wheat flour contaminated with PAs, but the incriminating plant remained unclear [295].

Two other outbreaks of HSOS were reported from India [296,297]. One of these was probably caused by consumption of cereals mixed with seeds of a plant (*Crotalaria* sp.) containing PAs, which occurred in the Sarguja district of India end of 1976; of the 67 recorded cases, 42% died [296]. The HSOS outbreaks were also highlighted in a recent systematic review article [298].

## 17. Conclusions

TCM herbs are highly appreciated around the world and easily accessible via the internet and thus can escape regulatory surveillance which is often not well established in many countries for this herbal TCM. Concomitantly, liver injury from TCM herbs emerges as a clinical problem and can evolve into acute liver failure in rare cases. Clinical signs are often non-specific, and there is no diagnostic feature in most cases that delineates liver injury due to herbal TCM from alternative causes of liver disease. Therefore, the diagnosis of liver injury induced by TCM herbs is a diagnosis of exclusion, for which RUCAM can aid the clinician to establish causality. Liver injury from herbal TCM is rare and similarly to drugs can be caused by an unpredictable idiosyncratic or a predictable intrinsic toxicity. Clinical features of liver injury from herbal TCM are variable. Specific diagnostic biomarkers such as microsomal epoxide hydrolase, pyrrole-protein adducts, metabolomics, and microRNA are available for only a few hepatotoxic TCM herbs. The diagnosis is ascertained if alternative causes are validly excluded and causality levels of probable or highly probable are achieved applying the liver specific RUCAM as the most commonly used diagnostic tool worldwide. Case evaluation may be confounded by inappropriate or lacking causality assessments, poor herbal product quality, insufficiently documented cases, and neglecting exclusion of alternative causes such as infections by hepatotropic viruses including hepatitis E virus. Herbal TCM used as medicine should be under stricter regulatory surveillance, considering these products as herbal drugs. Finally, clinical studies to evaluate the benefit/risk balance of herbal drugs should be encouraged.

## Figures and Tables

**Table 1:** Herbal TCM and related issues of concern.

Issues	Details of Issues and Proposed Solutions	First Author
Efficacy	Therapeutic efficacy is rarely provided for most TCM herbs. Randomized controlled trials (RCTs) are urgently needed, based on criteria of evidence based medicine (EBM).	Manheimer, 2009 [9]
		Teschke, 2015 [10]
		Ernst, 2006 [41]
		Wang, 2007 [42]
		Tang, 1999, 2008 [43,44]
Safety	Most TCM herbs are well tolerated by the majority of users, but few are not. RCTs should evaluate the individual benefit-risk profile for each herbal TCM product. It is important to define product quality specifications and adherence to the principles of cGAPs and cGMPs.	Ekor, 2014 [3]
		Liu, 2015 [8]
		Melchart, 2016 [13]
		Northier, 2000, 2002 [45,46]
		Vanherweghem, 1998 [47]
		Wagner, 2013 [48]
		WHO, 2016 [49]
		Frenzel, 2016 [50]
Hepatotoxicity	Causality assessment is often missing, must be verified by RUCAM for each spontaneous and clinical trial report submitted to regulatory agencies and for each case report submitted to a scientific journal for consideration of publication.	Frenzel, 2016 [50]
		Danan, 2016 [51]
Regulatory requirements	Less strict regulatory surveillance is risky for consumers, calling for substantial improvement. Regulatory definitions of indications and use are mandatory. Herbal TCM products for medicinal use should be approved by regulatory agencies and registered as herbal drugs, provided efficacy is validly established and their benefit-risk balance is favorable.	Liu, 2015 [8]
		Zhang, 2012 [12]
		Frenzel, 2016 [50]
Nomenclature	Variable nomenclature of herbal TCM products causes confusion, requiring CMM international name standards. Unlike Latin scientific names, CMM names, be they Chinese, pin yin, English, or according to pharmacopoeia, have not been standardized and their use, spelling and occasionally even the plant species to which they refer vary from publication to publication.	Chan, 2012 [52]

cGAPs, current Good Agricultural Practices; cGMPs, current Good Manufacturing Practices; CMM, Chinese Material Medical; RUCAM, Roussel Uclaf Causality Assessment Method.

**Table 2:** Adverse reactions of patients treated with herbal TCM.

Reported Side Effects	Patients (*n*)	Patients (%)
Liver adaptation	3	0.3
Liver injury	3	0.3
Diarrhea	46	4.6
Nausea	11	1.1
Vomiting	4	0.4
Other gastrointestinal symptoms	103	13.4
Neuro-vegetative symptoms	61	6.1
Aggravation of pain	36	3.6

**Table 3:** Compilation of reported herbal TCM products with suspected hepatotoxicity.

Chinese Name	Botanical Names, Main Ingredients
**Ai Ye**	*Artemisia argyi.*
**An Shu Ling**	*Lycopodium serratum* or rarely, *Corydalis* specie*s*, *Panax ginseng*, Pseudo ginseng, or two species of *Stephania.*
**Bai Fang**	*Angelica sinensis*, *Cyperus rotundus,* Ginseng, *Ligusticumwallichii*, *Paeonia alba*, *Rehmannia glutinosa.*
**Bai Shi Wan**	*Atractylis*, *Carthamus tinctorius*, *Dalbergia odorifera*, *Dioscorea bulbifera*, *Glycyrrhiza*, *Lithospermum erythrorhizon*, *Paeonia suffruticosa*, *Polygonum multiflorum*, *Psoralea corylifolia*, *Salvia miltiorrhiza*; *Endoconcha sepiae*, *Ganoderma lucidum* (mushroom).
**Bi Ma Zi**	*Rhicinus communis, Chaenomeles*, *Codonopsis pilosula*, *Notopterygium*, *Polygonum multiflorum*, *Rehmannia*, *Schisandra.*
**Bo He**	*Mentha haplocalyx.*
**Bo Ye Qing Niu Dan**	*Tinospora crispa.*
**Bofu Tsu Sho San**	*Angelica*, *Atractylis*, *Cnidium*, *Gardenia*, *Ephedra*, *Forsythia*, *Glycyrrhhiza*, *Gypsum fibrosum*, *Ledebouriella*, *Mentha*, *Paeonia*, *Platycodon*, *Rheum*, *Schizonepeta*, *Scutellaria*, *Zingiber ;* Kadinum (talcum powder), sodium sulfuricum.
**Boh Gol Zhee**	*Psoralea corylifolia.*
**Cang Er Zi**	*Xanthium sibiricum.*
**Chang Shan**	*Dichora febrifuga Lour.*
**Chai Hu**	*Bupleurum falcatum.*
**Chaso**	*Camellia sinensis*, *Cassia tora* (syn. *Senna*), *Crataegus*, N-nitroso-fenfluramine.
**Chi R Yun**	*Breynia officinalis.*
**Chinese herbal mixtures (various)**	*Dictamnus dasycarpus*, *Gentiana scabra, Hedyotis diffusa*, *Paeonia suffructicosa*, *Paris polyphylla*, *Rehmannia glutinosa*, S*milax glabra*, *Sophora subprostrata*; *Angelica sinensis*, *Bupleurum chinese*, *Dictamnus dasycarpus*, *Paeonia suffructiosa*, *Philodendron chinese*, *Saposhnikovia divaricata*, *Shisandra chinesis*, *Shizonepeta tenuifolia*, *Tribulus terrestris*; *Cocculus trilobus*, *Dictamnus dasycarpus*, *Eurysolen gracilis*, *Glycyrrhiza*, *Lophatherum*, *Paeonia*, *Potentilla*, *Rehmannia glutinosa*; *Alisma plantago aquatica*, *Artemisia capillaris*, *Gardenia jasminoidis*, *Gentiana scabra*, *Glycyrrhiza*, *Magnolia*, *Paeonia*, *Plantago asiatica*, *Saussurea Lappa.*
**Chuan Lian Zi**	*Melia toosendan.*
**Ci Wu Ji**	*Acanthopanax senticosus.*
**Da Chai Hu Tang**	*Bupleurum falcatum*, *Ginseng*, *Glycyrrhiza glabra*, *Pinellia*, *Scutellaria*, *Zingiber officinale*, *Zizyphus jujuba.*
**Da Huang**	*Rheum palmatum.*
**Du Huo**	*Angelica archangelica.*
**Fu Fang Qing Dai Wan**	*Angelica dahurica*, *Isatis indigotica* (Indigo naturalis), *Massa medicata fermentata* (yeast), *Salvia milthiorrhiza*, *Smilax glabra.*
**Gan Cao**	*Glycyrrhiza uralensis*, syn. Liquorice.
**Ge Gen**	*Pueraria lobata*, syn. Arrowroot.
**He Huan Pi**	*Albizia julibrissin.*
**Ho Shou Wu**	*Polygonum multiflorum*, syn. He Shou Wu.
**Hu Bohe You**	*Mentha pulegium*, syn. Pennyroyal oil.
**Hu Zhang**	*Polygonum cuspidatum.*
**Huang Qin**	*Scutellaria baicalensis.*
**Huang Yao Zi**	*Dioscorea bulbifera.*
**Hwang Geun Cho**	*Corydalis speciosa.*
**Ji Gu Cao**	*Abrus cantoniensis.*
**Ji Ji**	*Chloranthus serratus.*
**Ji Xue Cao**	*Centella asiatica*, syn. Gotu Kola.
**Jiguja**	*Hovenia dulcis.*
**Jin Bu Huan**	*Lycopodium serratum* or rarely, *Corydalis species*, *Panax ginseng*, Pseudo ginseng, or two species of *Stephania.*
**Jue Ming Zi**	*Cassia obtusifolia*, syn. *Senna.*
**Kamishoyosan**	*Angelica sinensis*, *Atractylodes racea*, *Bupleurum falcatum*, *Gardenia*, *Glycyrrhiza glabra*, *Mentha haplocalyx*, *Moutan*, *Paeonia alba*, *Sclerotium Poriae Cocos*, *Zingiber officinale.*
**Kudzu**	*Pueraria thunbergiana.*
**Ku Lian Zi**	*Melia azedarach.*
**Lei Gong Teng**	*Tripterygium wilfordii* Hook.
**Lui Hui**	*Aloe vera.*
**Long Dan Xie Gan Tang**	*Acebia*, *Alisma*, *Angelica sinensis*, *Bupleurum*, *Gardenia*, *Gentiana*, *Glycyrrhiza*, *Plantago*, *Rehmannia*, *Scutellaria.*
**Lu Cha**	*Camellia sinensis*, syn. Chinese green tea.
**Ma Huang**	*Ephedra sinica.*
**Mao Guo Tian Jie Cai**	*Heliotropium lasiocarpum.*
**Onshido**	*Aloe*, *Camellia sinensis*, *Crataegus*, G*ynostemma pentaphyllum makino*, *Raphanus*; N-nitroso-fenfluramine.
**Qian Li Guang**	*Senecio scandens.*
**Qibao Meiran Wan**	*Polygonum multiflorum*, *Angelica sinensis*, *fructus psoraleae* (Yan Shuizhi), wolfberry fruit, *Cuscuta* species, *poria cocos*, *achyranthes bidentata.*
**Ren Shen**	*Panax ginseng.*
**Sairei To**	*Alisma*, *Atractylis*, *Bupleurum*, *Cinnamomum*, *Ginseng*, *Glycyrrhiza*, *Pinellia*, *Polyporus*, *Poria*, *Scutellaria*, *Zingiber*, *Zizyphus.*
**Shan Chi**	*Gynura segetum.*
**Shang Lu**	*Phytolacca acinosa.*
**Shen Min**	Black cohosh, Burdock, Cayenne pepper, *Ginkgo biloba*, Horse chestnut, *Piper nigrum, Polygonum multiflorum*, *uva ursi*; biotin, collagen (hydrolyzed), niacin, pantothenic acid, silica (from plant sources), soy isoflavones, vitamin A, vitamin B_6_.
**Shi Can**	*Teucrium chamaedrys,* syn. Germander.
**Shi Liu Pi**	*Pericarpium granati.*
**ShouWu Pian**	*Achyranthes bidentata*, *Cuscuta chinensis*, *Eclipta prostrata*, *Ligustrum lucidum*, *Lonicera japonica*, *Morus alba*, *Polygonum multiflorum*, *Psoralea corylifolia*, *Rehmannia glutinosa*, *Rosa aevigat, Sesemum indicum*, *Siegesbeckia orientalis.*
**Tian Hua Fen**	*Trichosanthes kirilowii.*
**White flood**	Qian Ceng Ta (*Huperzia serrata*), Wu Zhu Yu E*vodia rutaecarpa*); beet root, caffein, cocoa bean, vinpocetine (from *Vinca* plant); acesulfame potassium, calcium silicate, carnitine tartrate, Carno-Syn® beta-alanine, citrulline, cryptoxanthin, folic acid, gamma-aminobutyric acid (GABA), glucuronolactone, selenium, L-norvaline, L-tyrosine, lutein, malic acid, ornithine, potassium gluconate, sucralose, sugar cane, water melon flavor, zeaxanthin.
**Wu Bei Zi**	*Galla chinensis.*
**Xi Shu**	*Camptotheca acuminate.*
**Xian Si Zi**	*Abrus Precatorius.*
**Xiao Chai Hu Tang**	*Bupleurum falcatum, Ginseng*, *Glycyrrhiza glabra*, *Pinellia tuber, Scutellaria baicalensis, Zingiber officinale*, *Zizyphus jujuba.*
**Yin Chen Hao**	*Artemisia capillaris.*
**Zexie**	*Alisma orientalis.*
**Zhen Chu Cao**	*Phyllanthus urinaria.*

Data are compiled from various reports, including references published previously [7,50,53,70,143,181,182]. In few cases, causality for specific TCM herbs or herbal mixtures was established by using RUCAM. For other cases, information was scarce and did not necessarily allow for causality assessment.

**Table 4:** Country/Area listing of selected reports of suspected HILI caused by TCM.

Country/Area	Cases (*n*)	References with First Author
Argentina	3	Jorge, 2005 [112]
Australia	1	Park, 2001 [156]
Canada	1	Divinsky, 2002 [116]
Columbia	1	Cárdenas, 2006 [151]
France	7	Larrey, 1992 [168]
	26	Castot, 1992 [169]
	1	Mostefa-Kara, 1992 [170]
	1	Diaz, 1992 [171]
	1	Ben Yahia, 1993 [172]
	1	Dao, 1993 [173]
	1	Mattéi, 1999 [175]
	1	Peyrin-Biroulet, 2004 [133]
Greece	1	Starakis, 2006 [176]
China	2	Dai, 2006 [124]
	1	Chen, 2007 [128]
	41	Wu, 2008 [119]
	1	Li, 2010 [129]
	116	Gao, 2012 [122]
	146	Zhou, 2013 [184]
	1	Li, 2015 [165]
	40	Wang, 2015 [164]
	15	Zhu, 2015 [189]
	563	Zhu, 2016 [188]
Hong Kong	4	Kumana, 1983, 1985 [125,126]
	1	But, 1996 [155]
	7	Yuen, 2006 [82]
	3	Cheung, 2009 [88]
	52	Lin, 2011 [120]
Italy	1	Picciotti, 1998 [117]
	1	Mazzanti, 2004 [157]
	36	Mazzanti, 2009 [138]
	1	Valente, 2010 [154]
	19	Mazzanti, 2015 [141]
Japan	4	Itoh, 1995 [178]
	1	Kamiyama, 1997 [91]
	12	Adachi, 2003 [93]
	1	Aiba, 2007 [166]
	1	Motoyama, 2008 [83]
	21	Gono, 2010 [106]
	1	Furukawa, 2010 [159]
	1	Tsuda, 2010 [167]
	1	Inoue, 2011 [92]
Korea	1	Hwang, 2001 [86]
	1	Nam, 2005 [87]
	4	Jang, 2008 [75]
	120	Kang, 2008 [79]
	24	Sohn, 2008 [80]
	1	Kang, 2009 [111]
	2	Kim, 2009 [104]
	1	Bae, 2010 [105]
	25	Jung, 2011 [153]
	1	Yang, 2012 [108]
	3	Kim, 2012 [113]
Macedonia	1	Huseini, 2016 [142]
Netherlands	1	Panis, 2005 [152]
Slovakia	1	Banarova, 2012 [160]
Spain	1	Garcia-Moran, 2004 [132]
	1	Jimenez-Saenz, 2006 [134]
	8	Garcia-Cortés, 2008 [136]
Taiwan	61	Lee, 2011 [90]
	2	Lin, 2002 [97]
	19	Lin, 2003 [98]
	1	Hsu, 2006 [179]
United Kingdom	1	Davies, 1990 [100]
	1	Graham-Brown, 1992 [76]
	1	Sanders, 1995 [101]
	3	Kane, 1995 [77]
	1	Vautier, 1995 [78]
	1	Skoulidis, 2005 [148]
United States	7	Woolf, 1994 [114]
	1	Nadir, 1996 [145]
	6	Horowitz, 1996 [115]
	1	Borum, 2001 [147]
	2	Haller, 2002 [70]
	7	Estes, 2003 [74]
	1	Bonkovsky, 2006 [135]
	1	Laird, 2008 [158]
	34	Sarma, 2008 [137]
	1	Linnebur, 2010, [107]
	1	Cohen, 2012 [177]
	1	Cortez, 2012 [81]
	1	Yang, 2012 [108]
	1	Dhanasekaran, 2013 [109]
	47	Navarro, 2013 [139]
	1	Papafragkakis, 2016 [110]

**Table 5:** Suspected toxic compounds implicated in liver injury from herbal TCM.

Chinese Name	Scientific Name	Tentative Hepatotoxic Components
Ai Ye	*Artemisia argyi*	Volatile oil
Bi Ma Zi	*Rhicinus communis*	Ricin, toxic proteins
Cang Shan	*Xanthium*	Glycosides (kaurene), diterpenoids
Chang Shan	*Dichor febrifuga Lour*	Alkaloids (dichroine)
He Huan Pi	*Albizia julibrissin*	Glycosides (saponine)
He Shou Wu	*Polygonum multiflorum*	Anthraquinones
Huang Yao Zi	*Discorea bulbifera L*	Glycosides (steroids, diosgenin), diterpenoids-lactones
Ku Lian Zi	*Melia azedarach*	Glycosides (tetranortriterpenoids)
Lei Gong Teng	*Tripterygium wilfordii hook F*	Glycosides (tripterygium), diterpenoid-lactones
Qian Li Guang	*Senecio scandens*	Pyrrolizidine alkaloids
Shan Lu	*Phytolacca acinosa Roxb*.	Alkaloids (phytolaccine)
Xiang Si Zi	*Abrus Precatorius*	Abrin

Data are adapted from reports of Ma et al. [181] and of Wu et al. [218].

**Table 6:** Clinical characteristics of the hepatic sinusoidal obstructive syndrome (HSOS) caused by *Gynura segetum* containing unsaturated PAs.

Conditions	Results
Cohort	*n* = 116
Gender	Males 57
	Females 56
	(NA 3)
Age	17–76 years
Ascites	115/116 cases
Hepatomegaly	104/113 cases
Jaundice	95/113 cases
ALT elevation	47/60 cases
	(NA 56 cases)
AST elevation	50/58 cases
	(NA 58 cases)
Outcome	Recovery 75 cases
	Chronicity 27 cases
	Death 11 cases
	(NA 3 cases)

Data from Gao et al., 2012 [122]. Abbreviations: ALT, Alanine aminotransferase; AST, Aspartate aminotransferase; NA, Not available; PAs, Pyrrolizidine alkaloids.

**Table 7:** Outcome of liver injury caused by selective TCM herbs.

TCM Product/TCM Herb	Fatality (*n*)	LTX (*n*)	Selected References with First Author
**Bai Xian Pi**		2	Sohn, 2008 [80]
**Ci Wu Jia**		2	Sohn, 2008 [80]
**Jiguja**		1	Sohn, 2008 [80]
**Lu Cha**		4	Mazzanti, 2015 [141]
**Ma Huang**		2	Estes, 2003 [74]
		1	Skoulidis, 2005 [148]
**Polygonum multiflorum**		3	Sohn, 2008 [80]
		1	Jung, 2011 [153]
	1	1	Wang, 2015 [164]
**San Chi**	1		Gao, 2012 [122]
**Sang Hwang**	11	1	Sohn, 2008 [80]
**Yin Chen Hao**		1	Sohn, 2008 [80]
**Korean case series of non HSOS**	3	3	Lee, 2015 [238]

LTX, Liver transplantation; HSOS, Hepatic sinusoidal obstruction syndrome; TCM, Traditional Chinese Medicine.

**Table 8:** Quality specifications for TCM herbs for safe use of consumers.

Herbal TCM product declaration of the manufacturer with address, phone and fax number, e-mail address
Expiration date of the herbal drug and herbal supplement
Batch number
Detailed recommendation for indication and contraindication
Advice for daily dose and maximum use duration
Correct labelling of all ingredients
Definition of plant family, subfamily, species, subspecies, and variety
Definition of plant part
Definition of used solvents and solubilizers
Exclusion of impurities, adulterants, and misidentifications
Minimum or lack of batch to batch variability
Minimum or lack of product to product variability
Current Good Agricultural Practices (cGAPs)
Current Good Manufacturing Practices (cGMPs)
Regulatory surveillance

Details are adapted from previous reports [50,60].

**Table 9:** Checklist of differential diagnoses in cases of liver injury due to TCM herbs.

Differential Diagnosis	Diagnostic Parameters	Diagnostic Exclusion Done for Patient’s Causality Assessment		
		Yes	No	Partial
Hepatitis A virus (HAV)	Anti-HAV-IgM	□	□	□
Hepatitis B virus (HBV)	HBV-DNA, anti-HBc-IgM	□	□	□
Hepatitis C virus (HCV)	HCV-RNA, anti-HCV	□	□	□
Hepatitis E virus (HEV)	HEV-RNA , titer change for anti-HEV-IgM/anti-HEV-IgG	□	□	□
Cytomegalovirus (CMV)	CMV-PCR, titer change for anti-CMV-IgM/anti-CMV-IgG	□	□	□
Epstein Barr virus (EBV)	EBV-PCR, titer change for anti-EBV-IgM/anti-EBV-IgG	□	□	□
Herpes simplex virus (HSV)	HSV-PCR, titer change for anti-HSV-IgM/anti-HSV-IgG	□	□	□
Varicella zoster virus (VZV)	VZV-PCR, titer change for anti-VZV-IgM/anti-VZV-IgG	□	□	□
Other virus infections according to the clinical context	Specific serology of Adenovirus, Coxsackie-B-Virus, Echovirus, Measles virus, Rubella virus, Flavivirus, Arenavirus, Filovirus, Parvovirus, HIV, and others	□	□	□
Other infectious diseases	Specific assessment of bacteria, fungi, parasites, worms, and others	□	□	□
Autoimmune hepatitis (AIH) type I	Gamma globulins, ANA, SMA, AAA, SLA/LP, Anti-LSP, Anti-ASGPR	□	□	□
Autoimmune hepatitis (AIH) type II	Gamma globulins, Anti-LKM-1 (CYP 2D6), Anti-LKM-2 (CYP 2C9), Anti-LKM-3	□	□	□
Primary biliary cholangitis (PBC)	AMA, Anti PDH-E2	□	□	□
Primary sclerosing cholangitis (PSC)	p-ANCA, MRC	□	□	□
Autoimmune cholangitis (AIC)	ANA, SMA	□	□	□
Overlap syndromes	See AIH, PBC, PSC, and AIC	□	□	□
Non alcoholic steatohepatitis (NASH)	BMI, insulin resistance, hepatomegaly, echogenicity of the liver	□	□	□
Alcoholic liver disease (ALD)	Patient’s history, clinical and laboratory assessment, other alcoholic disease(s)	□	□	□
Drug induced liver injury (DILI) or herb induced liver injury (HILI)	Patient’s history, clinical and laboratory assessment, sonography, use of the updated RUCAM	□	□	□
Cocaine, ecstasy and other amphetamines	Toxin screening	□	□	□
Rare intoxications	Toxin screening for household and occupational toxins	□	□	□
Hereditary hemochromatosis	Serum ferritin, total iron-binding capacity, genotyping for C2824 and H63D mutation, hepatic iron content	□	□	□
Wilson disease	Copper excretion (24 h urine), ceruloplasmin in serum, free copper in serum, Coombs-negative hemolytic anemia, hepatic copper content, Kayser-Fleischer-ring, neurologic-psychiatric work-up, genotyping	□	□	□
Porphyria	Porphobilinogen in urine, total porphyrines in urine	□	□	□
α_1-_Antitrypsin deficiency	α_1_—Antitrypsin in serum	□	□	□
Biliary diseases	Clinical and laboratory assessment, hepatobiliary sonography, MRC	□	□	□
Pancreatic diseases	Clinical and laboratory assessment, sonography, CT, MRT	□	□	□
Celiac disease	TTG antibodies, endomysium antibodies, duodenal biopsy	□	□	□
Anorexia nervosa	Clinical context	□	□	□
Parenteral nutrition	Clinical context	□	□	□
Cardiopulmonary diseases	Cardiopulmonary assessment of congestive heart disease, myocardial infarction, cardiomyopathy, cardiac valvular dysfunction, pulmonary embolism, pericardial diseases, arrhythmia, hemorrhagic shock, and various other conditions	□	□	□
Addison’s disease	Plasma cortisol	□	□	□
Thyroid diseases	TSH basal, T4, T3	□	□	□
Grand mal seizures	Clinical context of epileptic seizure (duration > 30 min)	□	□	□
Heat stroke	Shock, hyperthermia	□	□	□
Polytrauma	Shock, liver injury	□	□	□
Systemic diseases	Specific assessment of sarcoidosis, amyloidosis, metastatic tumor, sepsis, and others	□	□	□
Other diseases	Clinical context	□	□	□

**Table 10:** Core elements of RUCAM as compared to other causality methods

Items	RUCAM	MV	DILIN	Naranjo	WHO	AD HOC
Time frame of latency period (score)	+	+	0	0	0	0
Time frame of dechallenge (score)	+	+	0	0	0	0
Recurrent ALT or ALP increase (score)	+	0	0	0	0	0
Definition of risk factors (score)	+	0	0	0	0	0
All comedications (score)	+	0	0	+	0	0
Individual comedication (score)	+	0		0	0	0
Search for individual alternative causes (score)	+	+	0	0	0	0
Verified exclusion of specific alternative causes (score)	+	+	0	0	0	0
All specifically assessed HAV, HBV, HCV, HEV (score)	+	0	0	0	0	0
All specifically assessed CMV, EBV, HSV, VZV (score)	+	0	0	0	0	0
Evaluation of cardiac hepatopathy (score)	+	+	0	0	0	0
Liver and biliary tract imaging (score)	+	0	0	0	0	0
Color Doppler sonography of liver vessels (score)	+	0	0	0	0	0
Prior known hepatotoxicity (score)	+	+	0	+	0	0
Search for unintended reexposure (score)	+	+	0	+	0	0
Definition of unintended reexposure (score)	+	0	0	0	0	0
Qualified criteria of unintended reexposure (score)	+	0	0	0	0	0
Laboratory hepatotoxicity criteria	+	+	+	0	0	0
Laboratory hepatotoxicity pattern	+	+	+	0	0	0
Hepatotoxicity specific method	+	+	+	0	0	0
Structured, liver related method	+	+	0	0	0	0
Quantitative, liver related method	+	+	0	0	0	0
Validated method (gold standard)	+	0	0	0	0	0

Adapted from a previous report, which provides additional information as well as references for RUCAM and the other methods [51]. RUCAM, Roussel Uclaf Causality Assessment Method; MV, Maria & Victorino method; DILIN, Drug-Induced Liver Injury Network method, WHO method, WHO global introspection method. The symbol + shows that this specific item is published, and the symbol 0 indicate lacking publication. ALT: Alanine aminotransferase; ALP: Alkaline phosphatase; CMV: Cytomegalovirus; EBV: Epstein Barr virus; HAV: Hepatitis A virus; HBV: Hepatitis B virus; HCV: Hepatitis C virus; HEV: Hepatitis E virus; HSV: Herpes simplex virus; VZV: Varicella zoster virus.

**Table 11:** Listing of selected individual reports using RUCAM in suspected cases of liver injury caused by TCM herbs, updated and adapted from a previous report [51]. DILI, drug induced liver injury; HILI, herb induced liver injury; TCM, traditional Chinese medicine.

Cases	Products	Country/Region	Year	First Author
HILI	Ji Xue Cao	Argentina/South America	2005	Jorge [112]
HILI	Lu Cha (Green tea , *Camellia sinensis*)	France/Europe	2005	Gloro [278]
HILI	Bo He, Chuan Lian Zi, and various other herbal TCM	Korea/Asia	2006	Yuen [82]
HILI	Lu Cha	Spain/Europe	2006	Jimenez-Saenz [134]
HILI	*Polygonum multiflorum*	Columbia/South America	2006	Cárdenas [151]
HILI	Bai Xian Pi, Kudzu, Lu Cha, Yin Chen Hao	Korea/Asia	2008	Kang [111]
HILI	Bai Xian Pi, Ci Wu Jia, Shou Wu Pian, Yin Chen Hao	Korea/Asia	2008	Sohn [80]
HIL	*Polygonum multiflorum*	China/Asia	2009	Zhang [161]
HILI	Lu Cha	Italy/Europe	2009	Mazzanti [138]
HILI	*Corydalis speciosa*	Korea/Asia	2009	Kang [111]
HILI	Ge Gen	Korea/Asia	2009	Kim [104]
HILI	Ho Shou Wu	Korea/Asia	2010	Bae [105]
HILI	Aloe	Korea/Asia	2010	Yang [279]
HIL	*Polygonum multiflorum*	Korea/Asia	2011	Jung [153]
HILI	*Gynura segetum*	Hong Kong/Asia	2011	Lin [120]
HILI	Juguju	Korea/Asia	2012	Kim [113]
HILI	*Gynura segetum*	Hong Kong/Asia	2012	Gao [122]
HILI DILI	Multiple herbs synthetic drugs and	Korea/Asia	2012	Suk [182]
DILI HILI	Multiple TCM herbs and synthetic drugs	China/Asia	2014	Hao [186]
HILI	*Polygonum multiflorum*	China/Asia	2014	Dong [162]
HILI	Lu Cha	Italy/Europe	2015	Mazzanti [221]
HILI	*Polygonum multiflorum*	China/Asia	2015	Lei [163]
HILI	*Polygonum multiflorum*	China/Asia	2015	Wang [164]
HILI	*Polygonum multiforme*	China/Asia	2015	Zhu [187]
HILI	*Single TCM herbs*	Germany/Europe	2016	Douros [183]
HILI DILI	Multiple TCM herbs, synthetic drugs	Korea/Asia	2016	Woo [280]
HILI	Chinese skullcap, black catechu	USA	2016	Papafragkakis [110]
HILI	Various TCM herbs	Germany/Europe	2016	Melchart [14]

**Table 12:** Listing of selected international registries and regulatory agencies, and associated groups that applied RUCAM in suspected cases of liver injury due to TCM herbs.

Cases	Suspected Products	Country/Region	Year	First Author of the Group/Agency
HILI DILI	Various herbal TCM, synthetic drugs	Singapore/Asia	2006	Wai [283], National University of Singapore
HILI	Lu Cha	Sweden/Europe	2007	Björnsson [284], Swedish Adverse Drug Reactions Advisory Committee
HILI	Lu Cha, other TCM herbs	Spain/Europe	2008	García-Cortés [136], Spanish Liver Toxicity Registry
HILI DILI	Few TCM herbs, multiple drugs	Spain/Europe	2008	García-Cortés [285], Spanish Group for the Study of Drug-induced Liver Disease
HILI	Various TCM herbs	Hong Kong	2011	Chau [286], Hong Kong Herb-Induced Liver Injury Network (HK-HILIN), Hong Kong
HILI	*Polygonum multiflorum*	Korea/Asia	2011	Jung [153], School of Medicine, Changwon Gyeongsang National University School of Medicine, Jinju/Sungkyunkwan University

**Table 13:** Conditions of unintentional reexposure tests in cases of liver injury caused by TCM herbs.

Reexposure Test Result	Hepatocellular Injury		Cholestatic or Mixed Liver Injury	
	ALTb	ALTr	ALPb	ALPr
Positive	<5 *N*	≥2 ALTb	<2 N	≥2 ALPb
Negative	<5 *N*	<2 ALTb	<2 N	<2 ALPb
Negative	≥5 *N*	≥2 ALTb	≥2 N	≥2 ALPb
Negative	≥5 *N*	<2 ALTb	≥2 N	<2 ALPb
Uninterpretable	<5 *N*	n.a.	<2 N	n.a.
Uninterpretable	n.a.	≥2 ALTb	n.a.	≥2 ALPb
Uninterpretable	n.a.	n.a.	n.a.	n.a.

Conditions and criteria for an unintentional reexposure test are described in a previous report [51]. Accordingly, required data for the hepatocellular injury are the ALT levels just before reexposure, referred to as baseline ALT or ALTb, and the ALT levels during reexposure, referred to as ALTr. Response to reexposure is positive, if both criteria are met: first, ALTb is below 5 N with N as the upper limit of the normal value, and second, after reexposure, ALT should increase to at least twice the baseline ALT value (ALTr ≥2 ALTb). Other variations are evaluated as negative or uninterpretable results. For the cholestatic or mixed liver injury, corresponding values of ALP are to be used instead of ALT. Abbreviations: ALP, Alkaline phosphatase; ALT, Alanine aminotransferase; HILI, Herb induced liver injury; N, Upper limit of Normal; n.a., not available.

**Table 14:** Causality assessment by RUCAM and/or positive reexposure tests in cases with assumed liver injury due to selected herbal Traditional Chinese Medicine (TCM) products.

Herbal TCM	RUCAM Based Causality	Reexposure Based Causality
Aconitum carmichaeli	+	−
Bai Xian Pi	+	−
Bo He	+	−
Cassia obtusifolia	+	−
Ci Wu Jia	+	−
Chuan Lian Zi	+	−
Corydalis yanhusuo	+	−
Da Huang	+	−
Gan Cao	+	−
Ge Gen	+	−
Ho Shou Wu	+	−
Huang Qin	−	−
Hwang Geun Cho	−	+
Ji Gu Cao	−	+
Ji Xue Cao	−	−
Jin Bu Huan	+	+
Jue Ming Zi	+	−
Jiguja	+	−
Kudzu	−	−
Ling Yang Qing Fei Keli	+	−
Lu Cha	+	+
Ma Huang	−	+
Polygonum multiflorum	+	+
Psoralea corylifolia	+	−
Rhen Shen	+	−
Rheum officinale	+	−
Shou Wu Pian	+	+
Shan Chi	+	−
Shen Min	+	−
Syo Saiko To	+	+
Xiao Chai Hu Tang	−	+
Zexie	+	−
Zhen Chu Cao	+	−

RUCAM: Roussel Uclaf Causality Assessment Method. + represents causality was confirmed, - indicates that causality was not confirmed.

## References

[1] Wu W.Y., Yang W.Z., Hou J.J., Guo D.A. (2015). Current status and futures perspective in the globalization of Traditional Chinese Medicine. World J. Tradit. Chin. Med..

[2] Leonti M., Casu L. (2013). Traditional medicine and globalization: Current and future perspectives in ethnopharmacology. Front. Pharmacol..

[3] Ekor M. (2014). The growing use of herbal medicines: Issues relating to adverse reactions and challenges in monitoring safety. Front. Pharmacol..

[4] Chen X., Pei L., Lu J. (2013). Filling the gap between traditional Chinese medicine and modern medicine, are we heading to the right direction?. Complement. Ther. Med..

[5] Wang T. (2013). Development and expectation of modernization of herbal medicine. Zhongguo Zhong Xi Yi Jie He Za Zhi.

[6] NIH, National Center for Complementary and Alternative Medicine (NCCAM) (2016). Traditional Chinese Medicine: An Introduction. http://nccam.nih.gov/health/whatiscam/chinesemed.htm.

[7] (2016). National Institutes of Health (NIH) and LiverTox: Chinese and Other Asian Herbal Medicines. http://livertox.nih.gov/ChineseAndOtherAsianHerbalMedicines.htm.

[8] Liu S.H., Chuang W.C., Lam W., Jiang Z., Cheng Y.C. (2015). Safety surveillance of Traditional Chinese Medicine: Current and future. Drug Saf..

[9] Manheimer E., Wieland S., Kimbrough E., Cheng K., Berman B.M. (2009). Evidence from the Cochrane Collaboration for Traditional Chinese Medicine therapies. J. Altern. Complement. Med..

[10] Teschke R., Wolff A., Frenzel C., Eickhoff A., Schulze J. (2015). Herbal traditional Chinese medicine and its evidence base in gastrointestinal disorders. World J. Gastroenterol..

[11] Zhang G.P., Hou H.P., Ye Z.G. (2015). Toxicity classification and detoxification strategies of Chinese Materia Medica. World J. Tradit. Chin. Med..

[12] Zhang L., Yan J., Liu X., Ye Z., Yang X., Meyboom R., Chan K., Shaw D., Duez P. (2012). Pharmacovigilance practice and risk control of Traditional Chinese Medicine drugs in China: Current status and future perspective. J. Ethnopharmacol..

[13] Melchart D., Hager S., Dai J., Weidenhammer W. (2016). Quality control and complication screening programme of Chinese medicinal drugs at the first German hospital of Traditional Chinese Medicine—A retrospective analysis. Forsch. Komplementmed..

[14] Melchart D., Linde K., Weidenhammer W., Hager S., Shaw D., Bauer R. (1999). Liver enzyme elevations in patients treated with traditional Chinese medicine.

[15] Efferth T., Kaina B. (2011). Toxicities by herbal medicines with emphasis to Traditional Chinese medicine. Curr. Drug Metab..

[16] Chu H., Zhang A.H., Wang X.J. (2015). Metabolomics and its potential in drug discovery and development from TCM. World J. Tradit. Chin. Med..

[17] Pelkonen O., Xu O., Fan T.P. (2014). Why is research on herbal medicinal products important and how can we improve quality?. J. Tradit. Complement. Med..

[18] Efferth T., Romero M.R., Wolf D.G., Stamminger T., Marin J.J.G., Marschall M. (2008). The antiviral activities of Artemisinin and Artesunate. Clin. Infect. Dis..

[19] Guo D.A. (2015). Nobel Prize for Artemisinin inspires modern TCM Research. World J. Tradit. Chin. Med..

[20] Efferth T. (2015). Artemisinin-second career as anticancer drug?. World J. Tradit. Chin. Med..

[21] Tu Y., Ni M.Y., Zhong Y.R., Li L.N., Cui S.L., Zhang M.Q., Qang W.Z., Hi Z., Li X.T. (1982). Studies on the constituents of Artemisia annua Part II. Planta Medica.

[22] Tu Y. (1999). The development of new antimalarial drugs: Qinghaosu and dihydro-qinghaosu. Chin. Med. J..

[23] World Health Organization World Malaria Report 2014. World Health Organization, 2014. http://www.who.int/malaria/publications/world_malaria_report_2014/wmr-2014-no-profiles.pdf.

[24] Centers for Disease Control and Prevention (CDC) Anopheles mosquitoes. http://www.cdc.gov/malaria/about/biology/mosquitoes/.

[25] Teschke R., Zhang L. (2015). Chinese herbs and their molecules: Clinical and pathophysiological implications for the liver. J. Mol. Pathophysiol..

[26] Xue R., Fang Z., Zhang M., Yi Z., Wen C., Shi T. (2013). TCMID: Traditional medicine integrative database for herb molecular mechanism analysis. Nucleic Acids Res..

[27] Meshnick S.R. (2002). Artemisinin: Mechanisms of action, resistance, and action. Int. J. Parasitol..

[28] Bunchorntavakul C., Reddy K.R. (2013). Review article: Herbal and dietary supplement hepatotoxicity. Aliment. Pharmacol. Ther..

[29] Calitz C., du Plessis L., Gouws C., Steyn D., Steenekamp J., Muller C., Hamman S. (2015). Herbal hepatotoxicity: Current status, examples, and challenges. Expert Opin. Drug Metab. Toxicol..

[30] Harper D., Loewe M., Shaughnessy E. (1999). Warring States Natural Philosophy and Occult Thought. The Cambridge History of Ancient China: From the Origins of Civilization to 221 BC.

[31] Lu Z.J. (1985). The Yellow Emperor’s Inernal Classic, an ancient medical canon of traditional Chinese medicine. J. Tradit. Chin. Med..

[32] Du X. (1989). Study on the monographs about Jin Kui Yao Lue (synopsis of prescriptions of the Golden Chamber) in the past dynasties. J. Chin. Physician.

[33] Beijing Declaration on International Traditional Chinese Medicine Cooperation in Science and Technology. http://www.most.gov.cn/eng/pressroom/200712/t20071206_57649.htm.

[34] Hager S., Dai J., Fischer V., Lüthke F., Staudinger A. (2016). East Meets West: Synergy through Diversity. Forsch. Komplementmed..

[35] Zhang G.G., Lee W., Bausell B., Lao L., Handwerger B., Berman B. (2005). Variability in the traditional Chinese medicine (TCM) diagnoses and herbal prescription provided by three TCM practitioners for 40 patients with rheumatoid arthritis. J. Altern. Complement. Med..

[36] Yifan Y., Al-Khafaji M. (2010). The theory and concepts of Chinese herbal medicine. Chinese Herbal Medicines.

[37] Yang Y., Al-Khafaji M. (2010). The theory and concepts of Chinese herbal medicine. Chinese Herbal Medicines.

[38] Sun W., Yang L., Qiu Y., Ren J., Huang R., Fu J. (2011). Identify nature *N*-acylethanolamide-hydrolyzing acid amide (NAAA) inhibitor: Effect of angelicae pubescentis radix on anti-inflammation. China J. Chin. Mater. Med..

[39] Xu L.L., Wang L., Wang Y.Q. (2006). Effects of radix salvia militiorrhia, radix aconiti lateralis preparata and rhizoma anemarrhena on nitric oxide systems of endotoxemia mice. Chin. J. Integr. Med..

[40] Lipsky P.E., Tao X.L. (1997). A potential new treatment for rheumatoid arthritis: thunder god vine. Semin. Arthritis Rheum..

[41] Ernst E. (2006). Methodological aspects of Traditional Chinese Medicine (TCM). Ann. Acad. Med. Singap..

[42] Wang G., Mao B., Xiong Z.Y., Fan T., Chen X.D., Wang L., Liu G.J., Liu J., Guo J., Chang J. (2007). The quality of reporting of randomized controlled trials of traditional Chinese medicine: A survey of 13 randomly selected journals from mainland China. Clin. Ther..

[43] Tang J.L., Zhan S.Y., Ernst E. (1999). Review of randomised controlled trials of traditional Chinese medicine. BMJ.

[44] Tang J.L., Liu B.Y., Ma K.W. (2008). Traditional Chinese medicine. Lancet.

[45] Nortier J.L., Vanherweghem J.L. (2002). Renal interstitial fibrosis and urothelial carcinoma associated with the use of a Chinese herb (Aristolochia fangchi). Toxicology.

[46] Nortier J.L., Martinez M.C., Schmeiser H.H., Arlt V.M., Bieler C.A., Petein M., Depierreux M.F., de Pauw L., Abramowicz D., Vereerstraeten P. (2000). Urothelial carcinoma associated with the use of a Chinese herb (Aristolochia fangchi). N. Engl. J. Med..

[47] Vanherweghem L.J. (1998). Misuse of herbal remedies: The case of an outbreak of terminal renal failure in Belgium (Chinese herbs nephropathy). J. Altern. Complement. Med..

[48] Wagner H., Wagner H., Ulrich-Merzenich G. (2013). Introduction. Evidence and Rational Based Research on Chinese Drugs.

[49] WHO (World Health Organization) Traditional Medicine Strategy 2014–2023. http://apps.who.int/iris/bitstream/10665/92455/1/9789241506090_eng.pdf?ua=1.

[50] Frenzel C., Teschke R. (2016). Herbal hepatotoxicity: Clinical characteristics and listing compilation. Int. J. Mol. Sci..

[51] Danan G., Teschke R. (2016). RUCAM in drug and herb induced liver injury: The update. Int. J. Mol. Sci..

[52] Chan K., Shaw D., Simmonds M.S.J., Leon C.J., Xu Q., Lu A., Sutherland I., Ignatova S., Zhu Y.P., Verpoorte R. (2012). General practice in reviewing and publishing studies on herbal medicine, with special emphasis on traditional Chinese medicine and Chinese *materia medica*. J. Ethnopharmacol..

[53] Teschke R. (2014). Traditional Chinese Medicine induced liver injury. J. Clin. Transl. Hepatol..

[54] CAMbrella Report. http://cordis.europa.eu/result/rcn/57185_en.html.

[55] Teschke R., Wolff A., Frenzel C., Schulze J. (2014). Letter: Herbal hepatotoxicity—An update on traditional Chinese medicine preparations; authors’ reply. Aliment. Pharmacol. Ther..

[56] Tsai S.J., Ruan Y.X., Lee C.C., Lee M.S., Chiou W.Y., Lin H.Y., Hsu F.C., Su Y.C., HSU T.W., Hung S.K. (2014). Use of Chinese medicine among colorectal cancer patients: a nationwide population-based study. Afr. J. Tradit. Complement. Altern. Med..

[57] Efferth T., Wagner H., Ulrich-Merzenich G. (2013). Inhibition of ATP-binding cassette transporters by Chinese herbs and phytochemicals. Evidence and Rational Based Research on Chinese Drugs.

[58] Liu J., Wang S., Zhang Y., Fan H.T., Lin H.S. (2015). Traditional Chinese medicine and cancer: History, present situation, and development. Thoracic. Cancer.

[59] Zhou S., Koh H.L., Gao Y., Gong Z.Y., Lee E.J.D. (2004). Herbal bioactivation: The good, the bad, and the ugly. Life Sci..

[60] Teschke R., Eickhoff A. (2015). Herbal hepatotoxicity in traditional and modern medicine: Actual key issues and new encouraging steps. Front. Pharmacol..

[61] Avigan M.I., Mozersky R.P., Seeff L.B. (2016). Scientific and regulatory perspectives in herbal and dietary supplement associated hepatotoxicity in the United States. Int. J. Mol. Sci..

[62] Ernst E. (2002). Adulteration of Chinese herbal medicines with synthetic drugs: a systematic review. J. Intern. Med..

[63] Ernst E. (2002). Toxic heavy metals and undeclared drugs in Asian herbal medicines. Trends Pharmacol. Sci..

[64] Wu M.L., Deng J.F., Lin K.P., Tsai W.J. (2013). Lead, mercury, and arsenic poisoning due to topical use of traditional Chinese medicines. Am. J. Med..

[65] Ting A., Chow Y., Tan W. (2013). Microbial and heavy metal contamination in commonly consumed traditional Chinese herbal medicines. J. Tradit. Chin. Med..

[66] Bauer R., Gasser U., Oettmeier R., Rausch H. (2013). Quality of TCM drugs and TCM products—Current status in the European Union. Pharm. Read. Trib..

[67] Shaw D. (2010). Toxicological risks of Chinese herbs. Planta Med..

[68] Chan K. (2005). Chinese medicinal materials and their interface with Western medical concepts. J. Ethnopharmacol..

[69] (2005). Pharmacopoeia of the People’s Republic of China.

[70] Haller C.A., Dyer J.E., Ko R., Olson K.R. (2002). Making a diagnosis of herbal-related toxic hepatitis. West. J. Med..

[71] Kao W.F., Hung D.Z., Tsai W.J., Lin K.P., Deng J.F. (1992). Podophyllotoxin intoxication: Toxic effect of Bajiaolian in herbal therapeutics. Hum. Exp. Toxicol..

[72] Chou S.L., Chou M.Y., Kao W.F., Yen D.H., Yen L.Y., Huang C.I., Lee C.H. (2010). Bajiaolian poisoning—A poisoning with high misdiagnostic rate. Am. J. Emerg. Med..

[73] National Institutes of Health (NIH) and LiverTox: Drug record Ba Jiao Lian (*Dysosma pleianthum*). http://livertox.nih.gov/BaJiaoLian.htm.

[74] Estes J.D., Stolpman D., Olyaei A., Corless C.L., Ham J.M., Schwartz J.M., Orloff S. (2003). High prevalence of potentially hepatotoxic herbal supplement use in patients with fulminant hepatic failure. Arch. Surg..

[75] Jang J.S., Seo E.G., Han C., Chae H.B., Kim S.J., Lee J.D., Wang J.H. (2008). Four cases of toxic liver injury associated with Dictamnus dasycarpus. Korean J. Hepatol..

[76] Graham-Brown R. (1992). Toxicity of Chinese herbal remedies. Lancet.

[77] Kane J.A., Kane S.P., Jain S. (1995). Hepatitis induced by traditional Chinese herbs: Possible toxic components. Gut.

[78] Vautier G., Spiller R.C. (1995). Safety of complementary medicines should be monitored. BMJ.

[79] Kang S.H., Kim J.I., Jeong K.H., Ko K.H., Ko P.G., Hwang S.W., Kim E.M., Kim S.H., Lee H.Y., Lee B.S. (2008). Korean J. Hepatol..

[80] Sohn C.H., Cha M.I., Oh B.J., Yeo W.H., Lee J.H., Kim W., Lim K.S. (2008). Liver transplantation for acute toxic hepatitis due to herbal medicines and preparations. J. Korean Soc. Clin. Toxicol..

[81] Cortez E., Boulger C., Bernard A. (2012). Ban Tu Wan hepatotoxicity. BMJ Case Rep..

[82] Yuen M.F., Tam S., Fung J., Wong D.K.H., Wong B.C.Y., Lai C.L. (2006). Traditional Chinese Medicine causing hepatotoxicity in patients with chronic hepatitis B infection: A 1-year prospective study. Aliment. Pharmacol. Ther..

[83] Motoyama H., Enomoto M., Yasuda T., Fujii H., Kobayashi S., Iwai S., Morikawa H., Takeda T., Tamori A., Sakaguchi H. (2008). Drug-induced liver injury caused by a herbal medicine, bofu-tsu-sho-san. Nihon Shokakibyo Gakkai Zasshi.

[84] Sakamoto S., Takeshita S., Sassa S., Suzuki S., Ishikawa Y., Kudo H. (2005). Effects of colestimide and/or Bofu-tsusho-san on plasma and liver lipids in mice fed a high-fat diet. In Vivo.

[85] National Institutes of Health (NIH) and LiverTox: Drug record Ma Huang (*Ephedra sinica*). http://livertox.nih.gov/Ephedra.htm.

[86] Hwang S.H., Park J.A., Jang Y.S., Lee K.M., Lee D.S., Ahn B.M., Lee E.H. (2001). Case of acute cholestatic hepatitis caused by the seeds of Psoralea-corylifolia. Korean J. Hepatol..

[87] Nam S.W., Baek J.T., Lee D.S., Kang S.B., Ahn B.M., Chung K.W. (2005). A case of acute cholestatic hepatitis associated with the seeds of *Psoralea corylifolia* (Boh-Gol-Zhee). Clin. Toxicol..

[88] Cheung W.I., Tse M.L., Ngan T., Lin J., Lee W.K., Poon W.T., Mak T.W., Leung V.K.S., Chau T.N. (2009). Liver injury associated with the use of *Fructus Psoraleae* (Bol-gol-zhee or Bu-gu-zhi) and its related propriety medicine. Clin. Toxicol..

[89] Teschke R., Bahre R. (2009). Severe hepatotoxicity by Indian Ayurvedic herbal products: A structured causality assessment. Ann. Hepatol..

[90] Lee C.H., Wang J.D., Chen P.C. (2011). Risk of liver injury associated with Chinese herbal products containing *Radix bupleuri* in 639,779 patients with hepatitis B virus infection. Plos One.

[91] Kamiyama T., Nouchi T., Kojima S., Murata N., Ikeda T., Sato C. (1997). Autoimmune hepatitis triggered by administration of an herbal medicine. Am. J. Gastroenterol..

[92] Inoue H., Yamazaki S., Shimizu M., Uozki H., Goto T., Ohnishi S., Koike K. (2011). Liver injury induced by the Japanese herbal drug kamishoyosan. Gastroenterol. Hepatol..

[93] Adachi M., Saito H., Kobayashi H., Horie Y., Kato S., Yoshioka M., Ishii H. (2003). Hepatic injury in 12 patients taking the herbal loss aids Chaso and Onshido. Ann. Intern. Med..

[94] Lai V., Smith A., Thorburn D., Raman V.S. (2006). Severe hepatic injury and adulterated Chinese medicines. BMJ.

[95] Yuen Y.P., Lai C.K., Poon W.T., Ng S.W., Chan A.Y.W., Mak T.W.L. (2007). Adulteration of over-the-counter slimming products with pharmaceutical analogues—An emerging threat. Hong Kong Med. J..

[96] Kanda T., Yokosuka O., Tada M., Kurihara T., Yoshida S., Suzuki Y., Nagao K., Saisho H. (2003). *N*-nitroso-fenfluramine hepatotoxicity resembling chronic hepatitis. J. Gastroenterol. Hepatol..

[97] Lin T.J., Tsai M.S., Chiou N.M., Deng J.F., Chiu N.Y. (2002). Hepatotoxicity caused by Breynia officinalis. Vet. Hum. Toxicol..

[98] Lin T.J., Su C.C., Lan C.K., Jiang D.D., Tsai J.L., Tsai M.S. (2003). Acute poisonings with Breynia officinalis—An outbreak of hepatotoxicity. J. Toxicol. Clin. Toxicol..

[99] National Institutes of Health (NIH) and LiverTox: Drug record Chi R Yun (*Breynia officinalis*). http://livertox.nih.gov/ChiRYun.htm.

[100] Davies E.G., Pollock I., Steel H.M. (1990). Chinese herbs for eczema. Lancet.

[101] Sanders D., Kennedy N., McKendrick M.W. (1995). Monitoring the safety of herbal remedies: Herbal remedies have a heterogeneous nature. Br. Med. J..

[102] Yoshida E.M., McLean C.A., Cheng E.S., Blanc P.D., Somberg K.A., Ferrell L.D., Lake J.R. (1996). Chinese herbal medicine, fulminant hepatitis, and liver transplantation. Am. J. Gastroenterol..

[103] National Institutes of Health (NIH) and LiverTox: Drug record Sho-Saiko-To, Dai-Saiko-To, Xiao Chai Hu Tang (Herbal mixtures). http://livertox.nih.gov/ShoSaikoTo_DaiSaikoTo.htm.

[104] Kim S.Y., Yim H.J., Ahn J.H., Kim J.H., Kim J.N., Yoon I., Kim D.I., Lee H.S., Lee S.W., Choi J.H. (2009). Two cases of toxic hepatitis caused by arrowroot juice. Korean J. Hepatol..

[105] Bae S.H., Kim D.H., Bae Y.S., Lee K.J., Kim D.W., Yoon J.B., Hong J.H., Kim S.H. (2010). Toxic hepatitis associated with *Polygoni multiflori*. Korean J. Hepatol..

[106] Gono Y., Odaguchi H., Hayasaki T., Suzuki K., Oikawa T., Muranushi A., Akahoshi T., Hanawa T. (2010). Clinical analysis of cases with drug-induced liver injury for Kampo medicine. Kampo Med..

[107] Linnebur S.A., Rapacchietta O.C., Vejar M. (2010). Hepatotoxicity associated with Chinese skullcap contained in Move Free Advanced dietary supplement: Two case reports and review of the literature. Pharmacotherapy.

[108] Yang L., Aronsohn A., Hart J., Jensen D. (2012). Herbal hepatotoxicity from Chinese skullcap: A case report. World J. Hepatol..

[109] Dhanasekaran R., Owens V., Sanchez W. (2013). Chinese skullcap in Move Free arthritis supplement causes drug induced liver injury and pulmonary infiltrates. Case Rep. Hepatol..

[110] Papafragkakis C., Ona M.A., Reddy M., Anand S. (2016). Acute hepatitis after ingestion of a preparation of Chinese skullcap and black catechu for joint pain. Case Rep. Hepatol..

[111] Kang H.S., Choi H.S., Yun T.J., Lee K.G., Seo Y.S., Yeon J.E., Byun K.S., Um S.H., Kim C.D., Ryu H.S. (2009). A case of acute cholestatic hepatitis induced by *Corydalis speciosa Max*. Korean J. Hepatol..

[112] Jorge O.A., Jorge A.D. (2005). Hepatotoxicity associated with the ingestion of *Centella asiatica*. Rev. Esp. Enferm. Dig..

[113] Kim Y.J., Ryu S.L., Shim J.W., Kim D.S., Shim J.Y., Park M.S., Jung H.L. (2012). A pediatric case of toxic hepatitis induced by Hovenia dulcis. Pediatr. Gastroenterol. Hepatol. Nutr..

[114] Woolf G.M., Petrovic L.M., Rojter S.E., Wainwright S., Villamil F.G., Katkov W.N., Michieletti P., Wanless I.R., Stermitz F.R., Beck J.J. (1994). Acute hepatitis associated with the Chinese herbal product Jin Bu Huan. Ann. Intern. Med..

[115] Horowitz R.S., Feldhaus K., Dart R.C., Stermitz F.R., Beck J.J. (1996). The clinical spectrum of Jin Bu Huan toxicity. Arch. Intern. Med..

[116] Divinsky M. (2002). Case report: Jin Bu Huan—Not so benign herbal medicine. Can. Fam. Physician.

[117] Picciotti A., Campo N., Brizzolara R., Giusto R., Guido G., Sinelli N., Lapertosa G., Celle G. (1998). Chronic hepatitis induced by Jin Bu Huan. J. Hepatol..

[118] National Institutes of Health (NIH) and LiverTox: Drug record Jin Bu Huan (*Lycopodium serratum)*. http://livertox.nih.gov/JinBuHuan.htm.

[119] Wu G.L., Yu G.Y., Chen J. (2008). Clinical analysis of hepatic veno-occlusive disease induced by *Sedum aizoon*. Zhongguo Zhong Yao Za Zhi.

[120] Lin G., Wang J.Y., Li N., Li M., Gao H., Ji Y., Zhang F., Wang H., Zhou Y., Ye Y. (2011). Hepatic sinusoidal obstruction syndrome associated with consumption of Gynura segetum. J. Hepatol..

[121] Gao X.S., Xiao S.S., He J.F. (2006). Analysis of alkaloids in Sedum aizoon and establishment of hepatic veno-occlusive model in mice. Chin. J. Integr. Trad. West. Med. Dig..

[122] Gao H., Li N., Wang J.Y., Zhang S.C., Lin G. (2012). Definitive diagnosis of hepatic sinusoidal obstruction syndrome induced by pyrrolizidine alkaloids. J. Dig. Dis..

[123] Wang J.Y., Gao H. (2014). Tusanqi and hepatic sinusoidal obstruction syndrome. Dig. Dis..

[124] Dai H.F., Gao Y., Yang M., Yu C.H., Gu Z.Y., Chen W.X. (2006). Hepatic veno-occlusive disease induced by *Gynura segetum*: Report of two cases. Hepatobiliary Pancreat. Dis. Int..

[125] Kumana C.R., Ng M., Lin H.J., Ko W., Wu P.C., Todd D. (1985). Herbal tea induced hepatic veno-occlusive disease: Quantification of toxic alkaloid exposure in adults. Gut.

[126] Kumana C.R., Ng M., Lin H.J., Ko W., Wu P.C., Todd D. (1983). Hepatic veno-occlusive disease due to toxic alkaloid in herbal tea. Lancet.

[127] Culvenor C.C.J., Edgar J.A., Smith L.W., Kumana C.R., Lin H.J. (1986). *Heliotropium lasiocarpum* Fisch and Mey identified as cause of veno-occlusive disease due to herbal tea. Lancet.

[128] Chen M.Y., Cai J.T., Du Q. (2007). Hepatic veno-occlusive disease associated with the use of Gynura segetum. Eur. J. Intern. Med..

[129] Li C., Liang X.S., Li C.Z. (2010). Sinusoidal obstruction syndrome associated with the ingestion of gynura root. Clin. Toxicol..

[130] Fenkel J.M., Navarro V.J. (2011). Review: Herbal and dietary supplement-induced liver injury. Gastroenterol. Hepatol..

[131] Duenas Sadornil C., Fabregas Piugtio S., Durandez R. (2004). Hepatotoxicity due to *Camelia sinensis*. Med. Clin. (Barc.).

[132] Garcia-Moran S., Saez-Royuela F., Gento E., Lopez Morante A., Arias L. (2004). Acute hepatitis associated with *Camellia* tea and *Orthosiphon stamineus* ingestion. Gastroenterol. Hepatol..

[133] Peyrin-Biroulet L., Petitpain N., Kalt P., Ancel D., Petit-Laurent F., Trechot P., Barraud H., Bronowicki J.P. (2004). Probable hepatotoxicity from epigallocatecol gallate used for phytotherapy. Gastroenterol. Clin. Biol..

[134] Jimemez-Saenz M., del Martinez-Sanchez M.C. (2006). Acute hepatitis associated with the use of green tea infusions. J. Hepatol..

[135] Bonkovsky H.L. (2006). Hepatotoxicity associated with supplements containing Chinese green tea (*Camellia sinensis*). Ann. Intern. Med..

[136] García-Cortés M., Borraz Y., Lucena M.I., Peláez G., Salmerón J., Diago M., Martínez-Sierra M.C., Navarro J.M., Planas R., Soria M.J. (2008). Liver injury induced by “natural remedies”: An analysis of cases submitted to the Spanish Liver Toxicity Registry. Rev. Esp. Enferm. Dig..

[137] Sarma D.N., Barrett M.L., Chavez M.L., Gardiner P., Ko R., Mahady G.B., Marles R.J., Pellicore L.S., Giancaspro G.I., Low Dog T. (2008). Safety of green tea extract: A systematic review by the US Pharmacopeia. Drug Saf..

[138] Mazzanti G., Menniti-Ippolito F., Moro P.A., Cassetti F., Raschetti R., Santuccio C., Mastrangelo S. (2009). Hepatotoxicity from green tea: A review of the literature and two unpublished cases. Eur. J. Clin. Pharmacol..

[139] Navarro V.J., Bonkovsky H.L., Hwang S.I., Vega M., Barnhart H., Serrano J. (2013). Catechins in dietary supplements and hepatotoxicity. Dig. Dis. Sci..

[140] Teschke R., Zhang L., Melzer L., Schulze J., Eickhoff A. (2014). Green tea extract and the risk of drug-induced liver injury. Expert Opin. Drug Metab. Toxicol..

[141] Mazzanti G., Di Sotto A., Vitalone A. (2015). Hepatotoxicity of green tea: An update. Arch. Toxicol..

[142] Huseini A.B., Bekjarovski N. (2016). Hepatotoxicity after 51 days use of green tea. Sch. J. Med. Case Rep..

[143] Rossi S., Navarro V.J. (2014). Herbs and liver injury: A clinical perspective. Clin. Gastroenterol. Hepatol..

[144] National Institutes of Health (NIH) and LiverTox: Drug record Green tea (*Camellia sinensis*). http://livertox.nlm.nih.gov/GreenTea.htm.

[145] Nadir A., Agrawal S., King P.D., Marshall J.B. (1996). Acute hepatitis associated with the use of a Chinese herbal product, ma-huang. Am. J. Gastroenterol..

[146] Reuben A., Koch D.G., Lee W.M., the Acute Liver Failure Study Group (2010). Drug-induced acute liver failure: Results of a U.S. multicenter, prospective study. Hepatology.

[147] Borum M.L. (2001). Fulminant exacerbation of autoimmune hepatitis after the use of Ma Huang. Am. J. Gastroenterol..

[148] Skoulidis F., Alexander G.J., Davies S.E. (2005). Ma huang associated acute liver failure requiring liver transplantation. Eur. J. Gastroenterol. Hepatol..

[149] Joshi D., Cross T.J.S., Wong V.S. (2007). Acute drug induced hepatitis secondary to a weight loss product purchased over the internet. Nutr. J..

[150] National Institutes of Health (NIH) and LiverTox: Drug record Shou Wu Pian (Polygonum multiflorum). http://livertox.nih.gov/ShouWuPian.htm.

[151] Cárdenas A., Restrepo J.C., Sierra F., Correa G. (2006). Acute hepatitis due to shen-min: A herbal product derived from *Polygonum multiflorum*. J. Clin. Gastroenterol..

[152] Panis B., Wong D.R., Hooymans P.M., de Smet P.A.G.M., Rosias P.R. (2005). Recurrent toxic hepatitis in a Caucasian girl related to the use of Shou-Wu-Pian, a Chinese herbal preparation. J. Pediat. Gastroenterol. Nutr..

[153] Jung K.A., Min H.J., Yoo S.S., Kim H.J., Choi S.N., Ha C.Y., Kim H.J., Kim T.H., Jung W.T., Lee O.J. (2011). Drug-induced liver injury: Twenty five cases of acute hepatitis following ingestion of *Polygonum multiflorum* Thun. Gut Liver.

[154] Valente G., Sanges M., Campione S., Bellevicine C., de Franchis G., Sollazzo R., Matera D., Cimino L., Vecchione R., D’Arienzo A. (2010). Herbal hepatotoxicity: A case of difficult interpretation. Eur. Rev. Med. Pharmacol. Sci..

[155] But P.P.H., Tomlinson B., Lee K.L. (1996). Hepatitis related to the Chinese medicine Shou-wu-pian manufactured from Polygonum multiflorum. Vet. Hum. Toxicol..

[156] Park G.J.H., Mann S.P., Ngu M.C. (2001). Acute hepatitis induced by Shou-Wu-Pian, a herbal product derived from *Polygonum multiflorum*. J. Gastroenterol. Hepatol..

[157] Mazzanti G., Battinelli L., Daniele C., Mastroianni C.M., Lichtner M., Coletta S., Costantini S. (2004). New case of acute hepatitis following the consumption of Shou Wu Pian, a Chinese herbal product derived from *Polygonum multiflorum*. Ann. Intern. Med..

[158] Laird A.R., Ramchandani N., deGoma E.M., Avula B., Khan I.A., Gesundheit N. (2008). Acute hepatitis associated with the use of an herbal supplement (*Polygonum multiflorum*) mimicking iron-overload syndrome. Clin. Gastroenterol..

[159] Furukawa M., Kasajima S., Nakamura Y., Shouzushima M., Nagatani N., Takinishi A., Taguchi A., Fujita M., Niimi A., Misaka R. (2010). Toxic hepatitis induced by Show-Wu-Pian, a Chinese herbal preparation. Intern. Med..

[160] Banarova A., Koller T., Payer J. (2012). Toxic hepatitis induced by Polygonum multiflorum. Vnitr. Lek..

[161] Zhang L., Yang X., Sun Z., Qu Y. (2009). Retrospective study of adverse events of *Polygonum multiflorum* and risks control. Zhongguo Zhong Yao Za Zhi.

[162] Dong H., Slain D., Cheng J., Ma W., Liang W. (2014). Eighteen cases of liver injury following ingestion of *Polygonum multiflorum*. Complement. Ther. Med..

[163] Lei X., Chen J., Ren J., Li Y., Zhai J., Mu W., Zhang L., Zheng W., Tian G., Shang H. (2015). Liver damage associated with *Polygonum multiflorum* Thunb: A systematic review of case reports and case series. Evid. Based Complement. Altern. Med..

[164] Wang J., Ma Z., Niu M., Zhu Y., Liang Q., Zhao Y., Song J., Bai Z., Zhang Y., Zhang P. (2015). Evidence chain-based causality identification in herb-induced liver injury: Exemplification of a well-known liver-restorative herb Polygonum multiflorum. Front. Med..

[165] Li X., Qu C., He Q., Chen W., Zhang X., Liu X., Liu Y., Tang Y. (2015). Acute hepatitis induced by a Chinese herbal product Qibao Meiran Wan: A case study. Int. J. Clin. Exp. Med..

[166] Aiba T., Takahashi T., Suzuki K., Okoshi S., Nomoto M., Uno K., Aoyagi Y. (2007). Liver injury induced by a Japanese herbal medicine, sairei-to (TJ-114, Bupleurum and Hoelen combination, Chai-Ling-Tang). J. Gastroenterol. Hepatol..

[167] Tsuda T., Yashiro S., Gamo Y., Watanabe K., Hoshino T., Oikawa T., Hanawa T. (2010). Discrepancy between clinical course and drug-induced lymphocyte stimulation tests in a case of *saireito*-induced liver injury accompanied by Sjögren syndrome. J. Altern. Complement. Med..

[168] Larrey D., Vial T., Pauwels A., Castot A., Biour M., David M., Michel H. (1992). Hepatitis after germander (*Teucrium chamaedrys*) administration: another instance of herbal medicine hepatotoxicity. Ann. Intern. Med..

[169] Castot A., Larrey D. (1992). Hepatites observées au cours d’un traitement par un medicament ou d’une tisane contenant de la germander petit-chêne. Bilan des 26 cas rapportés aux centres régionaux de pharmacovigilance. Gastroenterol. Clin. Biol..

[170] Mostefa-Kara N., Pauwels A., Pines E., Biour M., Levy V.G. (1992). Fatal hepatitis after herbal tea (letter). Lancet.

[171] Diaz D., Ferroudji S., Heran B., Barneon G., Larrey D., Michel H. (1992). Hepatite aiguë à la germander petit-chêne. Gastroenterol. Clin. Biol..

[172] Ben Yahia M., Mavier P., Metreau J.M., Zafrani E.S., Fabre M., Gatineau-Saillant G., Dhumeaux D., Mallat A. (1993). Hepatite chronic active et cirrhose induites par la germander petit-chêne. Gastroenterol. Clin. Biol..

[173] Dao T., Peytier A., Galateau F., Valla A. (1993). Chronic hepatitis due to germander. Gastroenterol. Clin. Biol..

[174] LiverTox Germander. http://livertox.nih.gov/Germander.htm.

[175] Mattéi A., Rucay P., Samuel D., Feray C., Michel R., Bismuth H. (1995). Liver transplantation for acute liver failure after herbal medicine (*Teucrium polium*) administration (letter). J. Hepatol..

[176] Starakis I., Siagris D., Leonidou L., Mazakopakis E., Tsamandas A., Karatza C. (2006). Hepatitis caused by the herbal remedy*Teucrium polium* L. Eur. J. Gastroenterol. Hepatol..

[177] Cohen S.M., Heywood E., Pillai A., Ahn J. (2012). Hepatotoxicity associated with the use of White Flood, a nutritional supplement. Pract. Gastroenterol..

[178] Itoh S., Marutani K., Nishijima T., Matsuo S., Itabashi M. (1995). Liver injuries induced by herbal medicine, Syo-saiko-to (xiao-chai-hu-tang). Dig. Dis. Sci..

[179] Hsu L.M., Huang Y.S., Tsay S.H., Chang F.Y., Lee S.D. (2006). Acute hepatitis induced by Chinese hepatoprotective herb xiao-chai-hu-tang. J. Chin. Med. Assoc..

[180] Teschke R., Zhang L., Long H., Schwarzenboeck A., Schmidt-Taenzer W., Genthner A., Wolff A., Frenzel C., Schulze J., Eickhoff A. (2015). Traditional Chinese Medicine and herbal hepatotoxicity: A tabular compilation of reported cases. Ann. Hepatol..

[181] Ma X., Peng J.H., Hu Y.Y. (2014). Chinese herbal medicine-induced liver injury. J. Clin. Transl. Hepatol..

[182] Suk K.T., Kim D.J., Kim C.H., Park S.H., Yoon J.H., Kim Y.S., Baik G.H., Kim J.B., Kweon Y.O., Kim B.I. (2012). A prospective nationwide study of drug-induced liver injury in Korea. Am. J. Gastroenterol..

[183] Douros A., Bronder E., Andersohn F., Klimpel A., Kreutz R., Garbe E., Bolbrinker J. (2016). Herb-induced liver injury in the Berlin Case-Control Surveillance Study. Int. J. Mol. Sci..

[184] Zhou Y., Yang L., Liao Z., He X., Zhou Y. Guo H. (2013). Epidemiology of drug-induced liver injury in China: A systematic analysis of the Chinese literature including 21,789 patients. Eur. Gastroenterol. Hepatol..

[185] Teschke R., Wolff A., Frenzel C., Schulze J. (2014). Review article: Herbal hepatotoxicity—An update on Traditional Chinese Medicine preparations. Aliment. Pharmacol. Ther..

[186] Hao K., Yu Y., He C., Wang M., Wang S., Li X. (2014). RUCAM scale-based diagnosis, clinical features and prognosis of 140 cases of drug-induced liver injury. Zhonghua Gan Zang Bing Za Zhi.

[187] Zhu Y., Liu S.H., Wang J.B., Song H.B., Li Y.G., He T.T., Ma X., Wang Z.X., Wang L.P., Zhou K. (2015). Clinical analysis of drug-induced liver injury caused by Polygonum multiflorum and its preparations. Zhongguo Zhong Xi Yi Jie He Za Zhi.

[188] Zhu Y., Niu M., Chen J., Zou Z.S., Ma Z.J., Liu S.H., Wang R.L., He T.T., Song H.B., Wang Z.X. (2016). Comparison between Chinese herbal medicine and Western medicine-induced liver injury of 1985 patients. J. Gastroenterol. Hepatol..

[189] Zhu Y., Li Y.G., Wang J.B., Liu S.H., Wang L.F., Zhao Y.L., Bai Y.F., Wang Z.X., Li J.Y., Xiao X.H. (2015). Causes, features, and outcomes of drug-induced liver injury in 69 children from China. Gut Liver.

[190] Zhu Y., Li Y.G., Wang Y., Wang L.P., Wang J.B., Wang R.L., Wang L.F., Meng Y.K., Wang Z.X., Xiao X.H. (2016). Analysis of clinical characteristics in 595 patients with herb-induced liver injury. Zhongguo Zhong Xi Yi Jie He Za Zhi CJITWM.

[191] Patwardhan B., Warude D., Pushpangadan Bhatt N. (2005). Ayurveda and traditional Chinese medicine: A comparative overview. Evid. Based Complement. Altern. Med..

[192] Sarges P., Steinberg J.M., Lewis J.H. (2016). Drug-induced liver injury: Highlights from a review of the 2015 literature. Drug Saf..

[193] Urban T.J., Daly A.K., Aithal G.P. (2014). Genetic basis of drug-induced liver injury: Present and future. Semin. Liver Dis..

[194] Wu J.J., Ai C.Z., Liu Y., Zhang Y.Y., Jiang M., Fan X.R., Lv A.P., Yang L. (2012). Interactions between phytochemicals from Chinese Medicines and human cytochrome P450 enzymes. Curr. Drug Metab..

[195] Cho J., Yoon I.S. (2015). Pharmacogenetic interactions of herbs with cytochrome P450 and p-glycoprotein. Evid. Based Complement. Altern. Med..

[196] Ma K.F., Zhang X.G., Jia H.Y. (2014). CYP1A2 polymorphism in Chinese patients with acute liver injury induced by *Polygonum multiflorum*. Genet. Mol. Res..

[197] Zimmerman H.J. (1999). Hepatotoxicity.

[198] Teschke R., Danan G. (2016). Diagnosis and management of drug-induced liver injury (DILI) in patients with pre-existing liver disease. Drug Saf..

[199] Loeper J., Descatoire V., Letteron P., Moulis C., Degott C., Dansette P., Fau D., Pessayre D. (1994). Hepatotoxicity of germander in mice. Gastroenterology.

[200] Fau D., Lekehal M., Farrell G., Moreau A., Moulis C., Feldman G., Haouzi D., Pessayre D. (1997). Diterpenoids from germander, an herbal medicine, induce apoptosis in isolated rat hepatocytes. Gastroenterology.

[201] Larrey D., Faure S. (2011). Herbal medicine hepatotoxicity: A new step with development of specific biomarkers. J. Hepatol..

[202] Chen M., Borlak J., Tong W. (2013). High lipophilicity and high daily dose of oral medications are associated with significant risk for drug-induced liver injury. Hepatology.

[203] Yu K., Geng M., Zhang J., Wang B., Ilic K., Tong W. (2014). High daily dose and being a substrate of cytochrome P450 enzymes are two important predictors of drug induced liver injury. Drug Metab. Dis..

[204] Lammert C., Einarsson S., Saha C., Niklasson A., Bjornsson E., Chalasani N. (2008). Relationship between daily dose of oral medications and idiosyncratic drug-induced liver injury: Search for signals. Hepatology.

[205] Lammert C., Bjornsson E., Niklasson A., Chalasani N. (2010). Oral medications with significant hepatic metabolism at higher risk for hepatic adverse effects. Hepatology.

[206] Chen M., Suzuki A., Borlak J., Andrade R.J., Lucena M.I. (2015). Drug induced liver injury: Interactions between drug properties and host factors. J. Hepatol..

[207] Teschke R., Andrade R.J. (2015). Drug-induced liver injury: Expanding our knowledge by enlarging population analysis and appropriate prospective causality assessment. Gastroenterology.

[208] Teschke R., Wolff A., Frenzel C., Schulze J., Eickhoff A. (2012). Herbal hepatotoxicity: A tabular compilation of reported cases. Liver Int..

[209] Teschke R., Schwarzenboeck A., Eickhoff A., Frenzel C., Wolff A., Schulze J. (2013). Clinical and causality assessment in herbal hepatotoxicity. Expert Opin. Drug Saf..

[210] Teschke R., Schwarzenboeck A., Hennermann K.H. (2008). Kava hepatotoxicity: A clinical survey and critical analysis of 26 suspected cases. Eur. J. Gastroenterol. Hepatol..

[211] Pantano F., Tittarelli R., Mannochi G., Zaami S., Ricci S., Giorgetti R., Terranova D., Busardò F.P., Marinelli E. (2016). Hepatotoxicity induced by “the 3Ks”: Kava, Kratom and Khat. Int. J. Mol. Sci..

[212] Teschke R. (2010). Kava hepatotoxicity—A clinical review. Ann. Hepatol..

[213] Teschke R. (2010). Kava hepatotoxicity: Pathogenetic aspects and prospective considerations. Liver Int..

[214] Teschke R., Glass X., Schulze J. (2011). Herbal hepatotoxicity by Greater Celandine (*Chelidonium majus*): Causality assessment of 22 spontaneous reports. Regul. Toxicol. Pharmacol..

[215] Teschke R., Frenzel C., Glass X., Schulze J., Eickhoff A. (2012). Greater Celandine hepatotoxicity: A clinical review. Ann. Hepatol..

[216] Teschke R., Glass X., Schulze J., Eickhoff A. (2012). Suspected Greater Celandine hepatotoxicity: Liver specific causality evaluation of published case reports from Europe. Eur. J. Gastroenterol. Hepatol..

[217] Liu Y., Li Z., Liu X., Pan R. (2014). Review on the toxic effects of radix Bupleuri. Curr. Opin. Complement. Altern. Med..

[218] Wu X., Chen X., Huang Q., Fang D., Li G., Zhang G. (2012). Toxicity of raw and processed roots of *Polygonum multiflorum*. Fitoterapia.

[219] National Pharmacopoeia Committee (2010). Chinese Pharmacopoeia.

[220] Frank J., George T.W., Lodge J.K., Rodriguez-Mateos A.M., Spencer J.P.E., Minihane A.M., Rimbach G. (2009). Daily consumption of an aqueous green tea extract supplement does not impair liver function or alter cardiovascular disease risk biomarkers in healthy men. J. Nutr..

[221] Bun S.S., Bun H., Guédon D., Rosier C., Ollivier E. (2006). Effect of green tea extracts on liver functions in Wistar rats. Food Chem. Toxicol..

[222] Galati G., Lin A., Sultan A.M., O’Brien P.J. (2006). Cellular and in vivo hepatotoxicity caused by green tea phenolic acids and catechins. Free Radic. Biol. Med..

[223] Chan P.C., Ramot Y., Malarkey D.E., Blackshear P., Kissling G.E., Travlos G., Nyska A. (2010). Fourteen-week toxicity study of green tea extract in rats and mice. Toxicol. Pathol..

[224] Martin L.C. (2007). Tea. The Drink That Changed the World.

[225] Chow H.H.S., Hakim I.A., Vining D.R., Crowell J.A., Cordova C.A., Chew W.M., Xu M.J., Hsu C.H., Ranger-Moore J., Alberts D.S. (2006). Effects of repeated green tea catechins administration on human cytochrome P450 activity. Cancer Epidemiol. Biomark. Prev..

[226] Chacko S.M., Thambi P.T., Kuttan R., Nishigaki I. (2010). Beneficial effects of green tea: A literature review. Chin. Med..

[227] Gavilan J.C., Bermudez F.J., Salgado F., Pena D. (1999). Phytotherapy and hepatitis. Rev. Clin. Esp..

[228] Patel S.S., Beer S., Kearney D.L., Phillips G., Carter B.A. (2013). Green tea extract: A potential cause of acute liver failure. World J. Gastroenterol..

[229] Fu P.P., Xia Q., Lin G., Chou M.W. (2004). Pyrrolizidine alkaloids—Genotoxicity, metabolism enzymes, metabolic activation, and mechanisms. Drug Metab. Rev..

[230] Fu P.P., Yang Y.C., Xia Q., Chou M.W., Cui M.M., Lin G. (2002). Pyrrolizidine alkaloids—Tumorigenic components in Chinese herbal medicines and dietary supplements. J. Food Drug Anal..

[231] Roulet M., Laurini R., Rivier L., Calame A. (1988). Hepatic veno-occlusive disease in newborn infant of a woman drinking herbal tea. J. Pediatr..

[232] Committee on Herbal Medicinal Products (HMPC) (2014). Public statement on the use of herbal medicinal products containing toxic, unsaturated pyrrolizidine alkaloids (PAs). http://www.ema.europa.eu/docs/en_GB/document_library/Public_statement/2014/12/WC50017955.pdf.

[233] Allgaier C., Franz S. (2015). Risk assessment on the use of herbal medicinal products containing pyrrolizidine alkaloids. Regul. Toxicol. Pharmacol..

[234] Avula B., Wang Y.H., Wang M., Smillie T.J., Khan I.A. (2012). Simultaneous determination of sesquiterpenes and pyrrolizidine alkaloids from the rhizomes of *Petasites hybridus* (L.) G.M. et Sch. and dietary supplements using UPLC-UV and HPLC-TOF-MS methods. J. Pharm. Biomed. Anal..

[235] Schenk A., Drewe J., Siewert B. (2014). Determination of pyrrolizidine alkaloids in Petasites hybridus leaf native CO2-extract by using UHPLC-HRMS. Planta Med..

[236] Gao H., Ruan J.Q., Chen J., Li N., Ke C.Q., Ye Y., Lin G., Wang J.Y. (2015). Blood pyrrole-protein adducts as a diagnostic and prognostic index in pyrrolizidine alkaloid-hepatic sinusoidal obstruction syndrome. Drug Des. Develop. Ther..

[237] DeLeve L.D., Valla D.C., Garcia-Tsao G. (2009). Vascular disorders of the liver. Hepatology.

[238] Lee W.J., Kim H.W., Lee H.Y., Son C.G. (2015). Systematic review of herb-induced liver injury in Korea. Food Chem. Toxicol..

[239] Chalasani N.P., Hayashi P.H., Bonkovsky H.L., Navarro V.J., Lee W.M., Fontana R.J. (2014). ACG Clinical guideline: The diagnosis and management of idiosyncratic drug-induced liver injury. Am. J. Gastroenterol..

[240] Hayashi P.H., Fontana R.J., Chalasani N.P., Stolz A.A., Talwalkar J.A., Navarro V.J., Lee W.M., Davern T.J., Kleiner D.E., Gu J. (2015). For the US Drug-Induced Liver Injury Network Investigators. Under-reporting of poor adherence to monitoring guidelines for severe cases of Isoniazid hepatotoxicity. Clin. Gastroenterol. Hepatol..

[241] Teschke R., Schulze J., Eickhoff A., Wolff A., Frenzel C. (2015). Mysterious Hawaii liver disease case—Naproxen overdose as cause rather than OxyELITE Pro?. J. Liver Clin. Res..

[242] Teschke R., Schwarzenboeck A., Frenzel C., Schulze J., Eickhoff A., Wolff A. (2016). The mystery of the Hawaii liver disease cluster in summer 2013: A pragmatic and clinical approach to solve the problem. Ann. Hepatol..

[243] Yang M., Ruan J., Fu P.P., Lin G. (2016). Cytotoxicity of pyrrolizidine alkaloid in human hepatic parenchymal and sinusoidal endothelial cells: Firm evidence for the reactive metabolites mediated pyrrolizidine alkaloid-induced hepatotoxicity. Chem. Biol. Interact..

[244] Ruan J., Yang M., Fu P., Ye Y., Lin G. (2014). Metabolic activation of pyrrolizidine alkaloids: Insights into the structural and enzymatic basis. Chem. Res. Toxicol..

[245] DeLeve L.D., Wang X., Kuhlenkamp J.F., Kaplowitz N. (1996). Toxicity of azathioprine and monocrotoline in murine sinusoidal endothelial cells and hepatocytes: The role of glutathione and relevance to hepatic venoocclusive disease. Hepatology.

[246] Chojkier M. (2003). Hepatic sinusoidal-obstruction syndrome: Toxicity of pyrrolizidine alkaloids. J. Hepatol..

[247] Braet F., Wisse E. (2002). Structural and functional aspects of liver sinusoidal endothelial cell fenestrae: A review. Comp. Hepatol..

[248] DeLeve L.D., DeLeve L.D., Garcia-Tsao G. (2011). Vascular liver disease and the liver sinusoidal endothelial cell. Vascular Liver Disease: Mechanisms and Management.

[249] Senior J.R. (2014). New biomarker for drug induced liver injury: Are they really better? What do they diagnose?. Liver Int..

[250] Lewis J.H. (2015). The art and science of diagnosing and managing drug-induced liver injury in 2015 and beyond. Clin. Gastroenterol. Hepatol..

[251] McGill M.R., Jaeschke H. (2015). MicroRNAs as signaling mediators and biomarkers of drug- and chemical-induced liver injury. J. Clin. Med..

[252] Zheng J., Ji C., Lu X., Tong W., Fan X., Gao Y. (2015). Integrated expression profiles of mRNA and microRNA in the liver of Fructus Meliae Toosendan water extract injured mice. Front. Pharmacol..

[253] Enache L.S., Enache E.L., Ramière C., Diaz O., Bancu L., Sin A., André P. (2014). Circulating RNA molecules as biomarkers in liver disease. Int. J. Mol. Sci..

[254] Li L.M., Wang D., Zen K. (2014). MicroRNAs in drug-induced liver injury. Clin. Transl. Hepatol..

[255] Aithal G.P. (2015). Pharmacogenetic testing in idiosyncratic drug induced liver injury: Current role in clinical practice. Liver Int..

[256] Daly A.K., Donaldson P.T., Bhatnagar P., Shen Y., Pe’er I., Floratos A., Daly M.J., Goldstein D.B., John S., Nelson M.R., Graham J. (2009). For the DILIGEN Study & International SAE Consortium. *HLA-B*5701* genotype is a major determinant of drug-induced liver injury due to flucloxacillin. Nat. Genet..

[257] Lewis P.J.S., Dear J., Platt V., Simpson K.J., Craig D.G.N., Antoine D.J., French N.S., Dhaun N., Webb D.J., Costello E.M. (2011). Circulating microRNAs as potential markers of human drug-induced liver injury. Hepatology.

[258] Yang X., Salminen W.F., Schnackenberg L.K. (2012). Current and emerging biomarkers of hepatotoxicity. Curr. Biomark. Find.

[259] Thulin P., Nordahl G., Gry M., Yimer G., Aklillu E., Makonnen E., Aderaye G., Lindquist L., Mattsson C.M., Ekblom B. (2014). Keratin-18 and microRNA-122 complement alanine aminotransferase as novel safety biomarkers for drug-induced liver injury in two human cohorts. Liver Int..

[260] Su Y.W., Chen X., Jiang Z.Z., Wang T., Wang C., Zhang Y., Wen J., Xue M., Zhu D., Zhang Y. (2012). A panel of serum microRNAs as specific biomarkers for diagnosis of compound- and herb-induced liver injury in rats.

[261] Watkins P.B., Merz M., Avigan M.I., Kaplowitz N., Regev A., Senior J.R. (2014). The clinical liver safety assessment best practices workshop: Rationale, goals, accomplishments and the future. Drug Saf..

[262] Senior J.R. (2014). Evolution of the Food and Drug Administration approach to liver safety assessment for new drugs: Current status and challenges. Drug Saf..

[263] Merz M., Lee K.R., Kullak-Ublick G.A., Brueckner A., Watkins P.B. (2014). Methodology to assess clinical liver safety data. Drug Saf..

[264] Regev A., Seeff L.B., Merz M., Ormarsdottir S., Aithal G.P., Gallivan J., Watkins P.B. (2014). Causality assessment for suspected DILI during clinical phases of drug development. Drug Saf..

[265] Teschke R. (2010). Black cohosh and suspected hepatotoxicity—Inconsistencies, confounding variables, and prospective use of a diagnostic causality algorithm: A critical review. Menopause.

[266] Teschke R., Schulze J. (2010). Risk of kava hepatotoxicity and the FDA consumer advisory. J. Am. Med. Assoc..

[267] Teschke R., Qiu S.X., Xuan T.D., Lebot V. (2011). Kava and kava hepatotoxicity: Requirements for novel experimental, ethnobotanical, and clinical studies based on a review of the evidence. Phytother. Res..

[268] Teschke R., Lebot V. (2011). Proposal for a Kava Quality Standardization Code. Food Chem. Toxicol..

[269] Teschke R., Sarris J., Lebot V. (2011). Kava hepatotoxicity solution: A six point plan for new kava standardization. Phytomedicine.

[270] Teschke R. (2011). Special report: Kava and the risk of liver toxicity: Past, current, and future. Am. Herb. Prod. Assoc..

[271] Teschke R., Sarris J., Lebot V. (2011). Contaminant hepatotoxins as culprits for hepatotoxicity—Fact or fiction?. Phytother. Res..

[272] Teschke R., Sarris J., Schweitzer I. (2012). Kava hepatotoxicity in traditional and modern use: The presumed Pacific kava paradox hypothesis revisited. Br. J. Clin. Pharmacol..

[273] Larrey D., Pageaux G. (1995). Hepatotoxicity of herbal remedies and mushrooms. Semin. Liver Dis..

[274] Teschke R., Eickhoff A., Schwarzenboeck A., Schmidt-Taenzer W., Genthner A., Frenzel C., Wolff A., Schulze J. (2015). Clinical review: Herbal hepatotoxicity and the call for systematic data documentation of individual cases. J. Liver Clin. Res..

[275] Danan G., Bénichou C. (1993). Causality assessment of adverse reactions to drugs-I. A novel method based on the conclusions of international consensus meetings: Application to drug-induced liver injuries. J. Clin. Epidemiol..

[276] Bénichou C., Danan G., Flahault A. (1993). Causality assessment of adverse reactions to drugs-II. An original model for validation of drug causality assessment methods: Case reports with positive rechallenge. J. Clin. Epidemiol..

[277] Yuan D., Yang X., Guo J.C. (2016). A great honor and a hugh challenge for China: You-you TU getting the Nobel Prize in Physiology or Medicine. J. Zhejiang Univ.-Sci. B (Biomed. & Biotechnol.).

[278] Gloro R., Hourmand-Ollivier I., Mosquet B., Mosquet L., Rousselot P., Salamé E., Piquet M.A., Dao T. (2005). Fulminant hepatitis during self-medication with hydroalcoholic extract of green tea. Eur. J. Gastroenterol. Hepatol..

[279] Yang H.N., Kim D.J., Kim Y.M., Kim B.H., Sohn K.M., Choi M.J., Choi Y.H. (2010). Aloe-induced toxic hepatitis. J. Korean Med. Sci..

[280] Woo H.J., Kim H.Y., Choi E.S., Cho Y., Kim Y., Lee J.H., Jang E. (2015). Drug-induced liver injury: A 2-year retrospective study of 1169 hospitalized patients in a single medical center. Phytomedicine.

[281] Heidemann L.A., Navarro V.J., Ahmad J., Hayashi P.H., Stolz A., Kleiner D.E., Fontana R. (2016). Severe acute hepatocellular injury attributed to OxyELITE Pro: A case series. Dig. Dis. Sci..

[282] Teschke R., Eickhoff A. (2016). The Honolulu Liver disease cluster at the Medical Center: Its mysteries and challenges. Int. Mol. Sci..

[283] Wai CT. (2006). Presentation of drug-induced liver injury in Singapore. Singap. Med. J..

[284] Björnsson E., Olsson R. (2007). Serious adverse liver reactions associated with herbal weight loss supplements. J. Hepatol..

[285] García-Cortés M., Lucena M.I., Pachkoria K., Borraz Y., Hidalgo R., Andrade R.J. (2008). Evaluation of Naranjo Adverse Drug Reactions Probability Scale in causality assessment of drug-induced liver injury. Aliment. Pharmacol. Ther..

[286] Chau T.N., Cheung W.I., Ngan T., Lin J., Lee K.W.S., Poon W.T., Leung V.K.S., Mak T., Tse M.L., The Hong Kong Herb-Induced Liver Injury Network (HK-HILIN) (2011). Causality assessment of herb-induced liver injury using multidisciplinary approach and the Roussel Uclaf Causality Assessment Method (RUCAM). Clin. Toxicol..

[287] Teschke R., Frenzel C., Schulze J., Schwarzenboeck A., Eickhoff A. (2013). Herbalife hepatotoxicity: Evaluation of cases with positive reexposure tests. World J. Hepatol..

[288] Zambrone F.A.D., Correa C., Sampaio do Amaral L.M. (2015). A critical analysis of the hepatotoxicity cases described in the literature related to Herbalife^®^ products. Braz. J. Pharm. Sci..

[289] Mahady G.B., Low Dog T., Barrett M.L., Chavez M.L., Gardiner P., Ko R., Marles R.J., Pellicore L.S., Giancaspro G.I., Sarma D.N. (2008). United States Pharmacopeia review of the black cohosh case reports of hepatotoxicity. Menopause.

[290] Teschke R., Schulze J. (2012). Suspected herbal hepatotoxicity: Requirements for appropriate causality assessment by the US Pharmacopeia. Drug Saf..

[291] Liss G., Lewis J.H. (2009). Drug-induced liver injury: What was new in 2008?. Expert Opin. Drug Metab. Toxicol..

[292] Teschke R., Genthner A., Wolff A., Frenzel C., Schulze J., Eickhoff A. (2014). Herbal hepatotoxicity: Analysis of cases with initially reported positive reexposure tests. Dig. Liver Dis..

[293] Hillman L., Gottfried M., Whitsett M., Rakela J., Schilsky M., Lee W.M., Ganger D. (2016). Clinical features and outcomes of complementary and alternative medicine induced acute liver failure and injury.

[294] Mohabbat O., Younos M.S., Merzad A.A., Srivastava R.N., Sediq G.G., Aram G.N. (1976). An outbreak of hepatic veno-occlusive disease in north-western Afghanistan. Lancet.

[295] Kakar F., Akbarian Z., Leslie T., Mustafa M.L., Watson J., van Egmond H.P., Omar M.F., Mofleh J. (2010). An outbreak of hepatic veno-occlusive disease in western Afghanistan associated with exposure to wheat flour contaminated with pyrrolizidine alkaloids. J. Toxicol..

[296] Tandon R.K., Tandon B.N., Tandon H.D. (1976). Study of an epidemic of venoocclusive disease in India. Gut.

[297] Tandon B.N., Tandon H.D., Tandon R.K., Narndranathan M., Joshi Y.K. (1976). An epidemic of veno-occlusive disease of the liver in central India. Lancet.

[298] Abdualmjid R.J., Sergi C. (2013). Hepatotoxic botanicals—An evidence-based systematic review. J. Pharm. Pharm. Sci..

